# Effects of microbiota-directed foods in gnotobiotic animals and undernourished children

**DOI:** 10.1126/science.aau4732

**Published:** 2019-07-12

**Authors:** Jeanette L. Gehrig, Siddarth Venkatesh, Hao-Wei Chang, Matthew C. Hibberd, Vanderlene L. Kung, Jiye Cheng, Robert Y. Chen, Sathish Subramanian, Carrie A. Cowardin, Martin F. Meier, David O’Donnell, Michael Talcott, Larry D. Spears, Clay F. Semenkovich, Bernard Henrissat, Richard J. Giannone, Robert L. Hettich, Olga Ilkayeva, Michael Muehlbauer, Christopher B. Newgard, Christopher Sawyer, Richard D. Head, Dmitry A. Rodionov, Aleksandr A. Arzamasov, Semen A. Leyn, Andrei L. Osterman, Md Iqbal Hossain, Munirul Islam, Nuzhat Choudhury, Shafiqul Alam Sarker, Sayeeda Huq, Imteaz Mahmud, Ishita Mostafa, Mustafa Mahfuz, Michael J. Barratt, Tahmeed Ahmed, Jeffrey I. Gordon

**Affiliations:** 1 Edison Family Center for Genome Sciences and Systems Biology, Washington University School of Medicine, St. Louis, MO 63110, USA; 2 Center for Gut Microbiome and Nutrition Research, Washington University School of Medicine, St. Louis, MO 63110, USA; 3 Department of Pathology and Immunology, Washington University School of Medicine, St. Louis, MO 63110, USA; 4 Division of Comparative Medicine, Washington University, St. Louis, MO 63110, USA; 5 Department of Medicine, Washington University School of Medicine, St. Louis, MO 63110, USA; 6 Architecture et Fonction des Macromolécules Biologiques, Centre National de la Recherche Scientifique and Aix-Marseille Université, 13288 Marseille cedex 9, France; 7 Department of Biological Sciences, King Abdulaziz University, Jeddah, Saudi Arabia; 8 Chemical Sciences Division, Oak Ridge National Laboratory, Oak Ridge, TN 37830, USA; 9 Sarah W. Stedman Nutrition and Metabolism Center, Duke University Medical Center, Durham, NC 27710, USA; 10 Duke Molecular Physiology Institute, Duke University Medical Center, Durham, NC 27710, USA; 11 Department of Pharmacology and Cancer Biology, Duke University Medical Center, Durham, NC 27710, USA; 12 Department of Medicine, Duke University Medical Center, Durham, NC 27710, USA; 13 Department of Genetics, Washington University School of Medicine, St. Louis, MO 63110, USA; 14 Genome Technology Access Center, Washington University School of Medicine, St. Louis, MO 63110, USA; 15 A. A. Kharkevich Institute for Information Transmission Problems, Russian Academy of Sciences, Moscow 127994, Russia; 16 Infectious and Inflammatory Disease Center, Sanford Burnham Prebys Medical Discovery Institute, La Jolla, CA 92037, USA; 17 International Centre for Diarrhoeal Disease Research, Bangladesh (icddr, b), Dhaka 1212, Bangladesh

## Abstract

To examine the contributions of impaired gut microbial community development to childhood undernutrition, we combined metabolomic and proteomic analyses of plasma samples with metagenomic analyses of fecal samples to characterize the biological state of Bangladeshi children with severe acute malnutrition (SAM) as they transitioned, after standard treatment, to moderate acute malnutrition (MAM) with persistent microbiota immaturity. Host and microbial effects of microbiota-directed complementary food (MDCF) prototypes targeting weaning-phase bacterial taxa underrepresented in SAM and MAM microbiota were characterized in gnotobiotic mice and gnotobiotic piglets colonized with age- and growth-discriminatory bacteria. A randomized, double-blind controlled feeding study identified a lead MDCF that changes the abundances of targeted bacteria and increases plasma biomarkers and mediators of growth, bone formation, neurodevelopment, and immune function in children with MAM.

**INTRODUCTION:**

There is a dimension to post-natal human development that involves assembly of microbial communities in different body habitats, including the gut. Children with acute malnutrition have impaired development of their gut microbiota, leaving them with communities that appear younger (more immature) than those of chronologically age-matched healthy individuals. Current therapeutic foods given to children with acute malnutrition have not been formulated based on knowledge of how they affect the developmental biology of the gut microbiota. Moreover, they are largely ineffective in ameliorating the long-term sequelae of malnutrition that include persistent stunting, neurodevelopmental abnormalities, and immune dysfunction.

**RATIONALE:**

Repairing microbiota immaturity and determining the degree to which such repair restores healthy growth requires identification of microbial targets that are not only biomarkers of community assembly but also mediators of various aspects of growth. Identifying ingredients in complementary foods, consumed during the transition from exclusive milk feeding to a fully weaned state, that increase the representation and expressed beneficial functions of growth-promoting bacterial taxa in the developing microbiota could provide an effective, affordable, culturally acceptable, and sustainable approach to treatment.

**RESULTS:**

Metabolomic and proteomic analyses of serially collected plasma samples were combined with metagenomic analyses of serially collected fecal samples from Bangladeshi children with severe acute malnutrition (SAM) treated with standard therapy. The results provided a readout of their biological features as they transitioned from SAM to a state of persistent moderate acute malnutrition (MAM) with accompanying persistent microbiota immaturity. Significant correlations were identified between levels of plasma proteins, anthropometry, plasma metabolites, and the representation of bacteria in their microbiota. Gnotobiotic mice were subsequently colonized with a defined consortium of bacterial strains that represent various phases of microbiota development in healthy Bangladeshi children. Administration of different combinations of Bangladeshi complementary food ingredients to colonized mice and germ-free controls revealed diet-dependent increases in the abundance and changes in the metabolic activities of targeted weaning-phase strains as well as diet- and colonization-dependent augmentation of growth-promoting host signaling pathways. Host and microbial effects of microbiota-directed complementary food (MDCF) prototypes were subsequently examined in gnotobiotic mice colonized with immature microbiota from children with post-SAM MAM and in gnotobiotic piglets colonized with a defined consortium of targeted age- and growth-discriminatory taxa. A randomized, double-blind study of standard therapy versus various MDCF prototypes emerging from these preclinical models, conducted in Bangladeshi children with MAM, identified a lead MDCF that increased levels of biomarkers and mediators of growth, bone formation, neurodevelopment, and immune function toward a state resembling healthy children. Using an approach inspired by statistical methods applied to financial markets, we show in the accompanying paper by Raman *et al*. that this lead MDCF was most effective in repairing the microbiota.

**CONCLUSION:**

These findings demonstrate the translatability of results obtained from pre-clinical gnotobiotic animal models to humans, directly support the hypothesis that healthy microbiota development is causally linked to healthy growth, illustrate an approach for treating childhood undernutrition, and with the capacity to deliberately reconfigure immature microbiota, suggest a means to decipher how elements of the gut microbial community operate to regulate various host systems involved in healthy growth.

**Figure:**
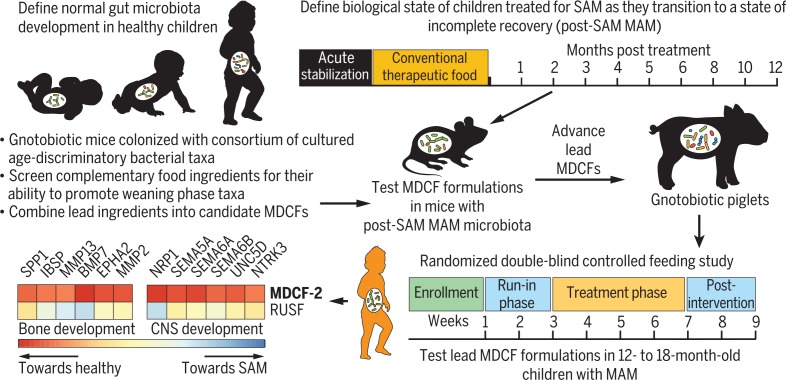
**Overview of therapeutic food discovery and testing**. The approach used for integrating preclinical gnotobiotic animal models with human studies to understand the contributions of perturbed gut microbiota development to childhood malnutrition and to identify MDCFs.

Evidence is accumulating that disruption of “normal” gut community (microbiota) development may contribute to the pathogenesis of undernutrition. Using culture-independent surveys, bacterial membership has been defined in fecal samples collected monthly during the first 2 postnatal years from healthy members of a birth cohort living in an urban slum (Mirpur) in Dhaka, Bangladesh (*1*, *2*). Applying machine learning [Random Forests (RF)] to the resulting 16S ribosomal DNA (rDNA) data-set yielded a “sparse” model composed of the most age-discriminatory bacterial strains; changes in the relative abundances of these organisms described a program of normal microbiota development (*2*). This RF-derived model was subsequently used to characterize fecal samples collected from Bangladeshi children with severe acute malnutrition [SAM; defined as a weight-for-height z-score (WHZ) >3 standard deviations below the median for a World Health Organization (WHO) reference healthy growth cohort (*3*)]. The results revealed gut communities that resembled those of healthy children who were chronologically younger. This microbiota “immaturity” was more pronounced in children with SAM as compared with those with moderate acute malnutrition (MAM; WHZ score between –2 and –3) and was not repaired in a clinical study that tested the effects of two therapeutic foods (*2*).

Impaired microbiota development has also been documented in undernourished Malawian children (*4*). To examine the functional importance of this impairment, microbial communities from healthy and stunted or underweight 6- and 18-month-old Malawian children were transplanted into groups of recently weaned germ-free mice fed a diet representative of that consumed by the human donor population. The results disclosed that compared with mice colonized with normally maturing microbiota from the healthy donors, animals harboring immature microbiota exhibited reduced rates of lean body mass gain, alterations in bone growth, and metabolic abnormalities (*4*). These studies provided preclinical evidence for a causal link between microbiota immaturity and undernutrition; they also revealed that a subset of the age-discriminatory strains is growth-discriminatory. Moreover, a cultured consortium of these age- and growth-discriminatory taxa ameliorated the impaired growth phenotype transmitted to recipient gnotobiotic mice by an immature microbiota (*4*).

One question arising from these observations is, how do we design optimal foods that steer a microbiota into an age-appropriate and healthy state? Breastfeeding plays a major role in reducing childhood malnutrition. As such, WHO and the United Nations Children’s Fund (UNICEF) recommend exclusive breastfeeding for the first 6 months of postnatal life and continued breast-feeding after the introduction of complementary foods up to 24 months of age (*5*). Suboptimal complementary feeding practices are important contributors to malnutrition in children less than 2 years of age (*6*). However, current complementary feeding recommendations are not based on knowledge of how foods affect the developmental biology of the gut microbiota during the weaning process. Together, these observations raise the question: Do certain complementary food ingredients or combinations of ingredients have the ability to selectively increase the representation and expressed beneficial functions of age- and growth-discriminatory strains deficient in SAM- or MAM-associated microbiota? If the answer is yes, then prescribed feeding of these ingredients could help “repair” or prevent development of microbiota immaturity in children, with potentially long-lived, health-promoting effects.

Here, we describe a process for identifying microbiota-directed complementary foods (MDCFs) designed to treat children with acute malnutrition. We first characterized gut microbial community and host responses over the course of 12 months in Bangladeshi children who were treated for SAM with one of three conventional therapeutic foods. Measurement of the levels of 1305 human plasma proteins—including regulators and effectors of physiologic, metabolic, and immune functions—combined with mass spec-trometric profiling of plasma metabolites and culture-independent analyses of serially collected fecal samples provided a “readout” of the biological features of these children as they progressed from SAM to a state of incomplete recovery (post-SAM MAM) with persistent microbiota immaturity. This readout included correlations between plasma proteins, anthropometry, plasma metabolites, and the representation of age-discriminatory members of their microbiota. We then screened complementary foods in gnotobiotic mice colonized with a consortium of bacterial strains that had been cultured from children living in Mirpur to identify ingredients that promote the representation of constituent age-discriminatory strains that are underrepresented in the setting of acute malnutrition. Subsequently, a representative microbiota from a child with post-SAM MAM was transplanted into gnotobiotic mice. Recipient animals were fed a diet resembling that consumed by children in Mirpur but supplemented with ingredients identified in the screen, in order to establish whether one or more of these MDCF formulations could repair a microbiota from a subject who had already received conventional therapy. Lead formulations were subsequently tested in gnotobiotic piglets colonized with a defined consortium of age- and growth-discriminatory strains to test their biological effects in a host species that is physiologically and metabolically more similar to humans than mice. Last, three MDCF prototypes were administered to children with MAM, and their effects on the microbiota and host biological state were determined.

## Effects of conventional therapeutic foods on the biological state of children with SAM

**Fig. 1:**
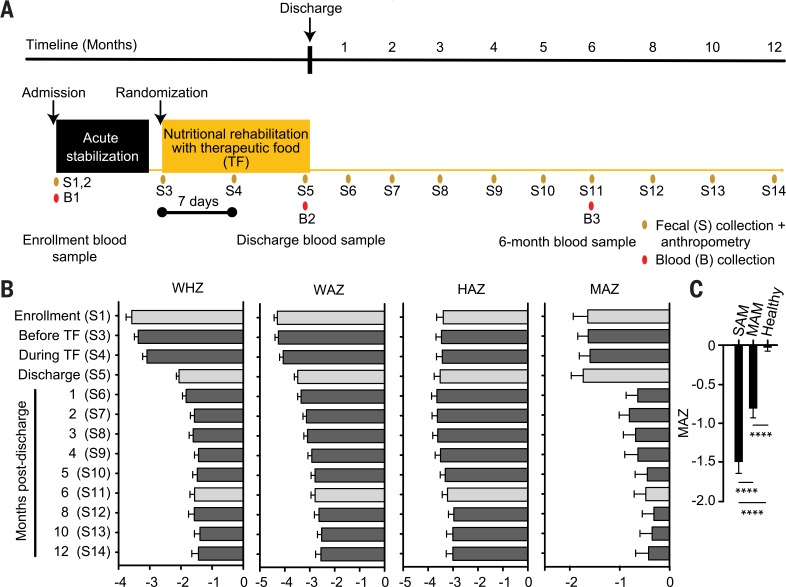
**Longitudinal study of Bangladeshi children with SAM treated with therapeutic foods.** (**A**) Study design. (**B**) Anthropometry and MAZ scores. Gray bars represent the three time points at which blood samples were collected. (**C**) Summary of MAZ scores for children with SAM (WHZ < –3; *n* = 96 fecal samples) and subsequently (post-SAM) MAM (WHZ > –3 and <–2; *n* = 151 fecal samples), plus healthy children aged 6 to 24 months living in the same area in which the SAM study was conducted (*n* = 450 fecal samples). Mean values for WHZ, WAZ, HAZ, and MAZ ± SEM are plotted on the x axes of (B) and (C). ****P < 0.0001 (one-way ANOVA followed by Tukey’s multiple comparisons test).

A total of 343 Bangladeshi children aged 6 to 36 months with SAM were enrolled in a multicenter, randomized, double-blind “noninferiority” study designed to compare two locally produced therapeutic foods (supplementary materials, materials and methods) with a commercially available, ready-to-use therapeutic food (RUTF) (*7*)(study design is provided in Fig. 1A, and the compositions of these therapeutic foods are available in table S1A). Children received standard management for SAM during the acute stabilization phase of in-hospital treatment, including a short course of antibiotics. Eligible children were then randomized to one of the three therapeutic food arms (~200 kcal/kg/day, mean duration 16.1 ± 10.3 days) (table S1B). Children were discharged after meeting criteria described in the supplementary materials, materials and methods. In a subset of 54 children, fecal samples were collected at enrollment [age 15.2 ± 5.1 months (mean ± SD)] before randomization, twice during treatment with a therapeutic food, and at regular intervals up to 12 months after discharge (Fig. 1A; clinical metadata is available in table S1B). Blood samples (plasma) were also obtained at enrollment, discharge, and 6 months after discharge for targeted mass spectrometry (MS)–based metabolic profiling; a sufficient quantity of blood was obtained from eight children at all three time points for aptamer-based proteomics analysis (*8*–*10*). Of these children, 44% had MAM at 12 months of followup. None of the therapeutic foods produced a significant effect on their severe stunting [height-for-age *z*-score (HAZ)] (Fig. 1B and table S1B).

### Metabolic phenotypes

**Fig. 2:**
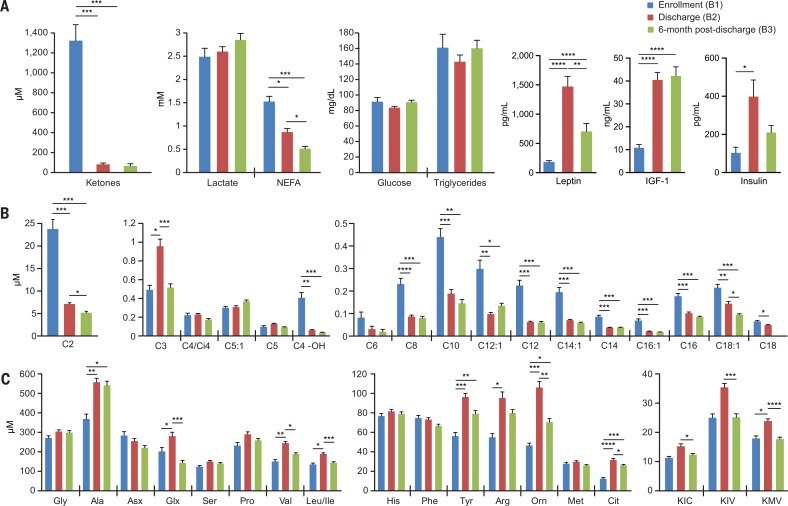
**Metabolic features of children with SAM before and after treatment.** (**A** to **C**) Levels of (A) standard clinical metabolites and selected hormones, (B) acylcarnitines, and (C) amino acids and ketoacids in plasma collected from children at enrollment (Fig. 1A, B1 blood sample), discharge (Fig. 1A, B2 sample), and 6 months after discharge (Fig. 1A, B3 sample). Abbreviations for branched chain ketoacids in (C) are KIC, a-ketoisocaproate; KIV, a-ketoisovalerate; and KMV, a-keto-b-methylvalerate. Mean values ± SEM are plotted. *P < 0.05; **P < 0.01; ***P < 0.001; ****P < 0.0001 (paired t test followed by FDR correction).

Targeted MS of plasma samples obtained at enrollment revealed high levels of ketones, nonesterified fatty acids (NEFA), and medium to long even-chain acylcarnitines (Fig. 2, A and B, and table S2), which is consistent with the known acute malnutrition-induced lipolytic response that raises circulating fatty acids and activates fatty acid oxidation (*11*). By discharge, this metabolic feature had normalized, whereas levels of a number of amino acids had increased significantly, including the gluconeogenic amino acid alanine; the branched-chain amino acids leucine, isoleucine, and valine; plus products of branched-chain amino acid metabolism [C3 (propionyl)-carnitine and their ketoacids] (Fig. 2, A to C). These findings suggest that the increased protein provided by the therapeutic foods prompted a switch from fatty acid to amino acid oxidation, leading to repletion of fat depots, increases in plasma leptin (Fig. 2A), and weight gain (table S1B). However, 6 months after treatment, multiple plasma amino acids and their metabolites had declined to levels comparable with those at admission, whereas fatty acids and fatty acid– derived metabolites remained at similar concentrations to those observed at discharge (Fig. 2, A to C). Insulin-like growth factor 1 (IGF-1) levels did not change significantly during this period (Fig. 2A), potentially explaining the absence of a signature of pronounced lipolysis that had been observed at enrollment. Although the suppression of lipolysis at 6 months after discharge suggests a sustained effect of nutritional resuscitation, the fall in essential amino acids and the lower level of IGF-1 compared with that found in similarly aged healthy children from the same community (44.5 versus 69.4 ng/mL; *P* = 0.02, *t* test) may contribute to the observed failure to achieve catch-up growth.

### The plasma proteome

Significant correlations between levels of plasma proteins, anthropometric indices, plasma metabolites, and host signaling pathways regulating key facets of growth are described in the supplementary text, results (table S3, A and B)—for example, components of the growth hormone (GH)–IGF axis, including soluble growth hormone receptor (also known as growth hormone binding protein), multiple IGF binding proteins (IGFBPs), and regulators of IGFBP turnover (the metalloprotease pappalysin-1 and its inhibitor stanniocalcin-1).

### The gut microbiome

A sparse RF-derived model of normal gut micro-biota development comprising 30 bacterial taxa [operational taxonomic units (OTUs)] and obtained from 25 healthy members of a birth cohort living in Mirpur (table S4, A to C) was applied to bacterial V4-16S rDNA datasets generated from fecal samples serially collected from the children in the SAM study (*n* = 539 samples). This model allowed us to define microbiota-for-age *z*-scores (MAZ) as a function of treatment arm and time [9.3 ± 3.7 samples/child (mean ± SD)]. The MAZ score measures the deviation in development of a child’s microbiota from that of chronologically age-matched reference healthy children on the basis of the representation of the ensemble of age-discriminatory strains contained in the RF-derived model (*2*). Significant microbiota immaturity was apparent in the SAM and post-SAM MAM groups (Fig. 1C and table S5A). Moreover, MAZ-scores in this SAM cohort were significantly correlated with WHZ, HAZ, and WAZ [Pearson correlation coefficient (*r*)= 0.16, *P* = 0.0004; *r* =0.13, *P* =0.003;and *r* =0.10, *P* = 0.02, respectively]. The MAZ score was not different at discharge but improved 1 month later (*P* = 0.0051 versus admission, Mann-Whitney test). This improvement could reflect increased dietary diversity, reduced antibiotic usage (table S1B), and/or other factors associated with returning to the home environment. MAZ-scores did not change significantly thereafter (Fig. 1B).

A number of the age-discriminatory strains were significantly correlated with anthropometric indices as well as with plasma proteins involved in biological processes that mediate growth. We also identified significant negative correlations between these taxa and mediators of systemic inflammation and anorexia/cachexia. *Bifidobacterium longum* (OTU 559527) had the greatest number of significant correlations (114) [table S3C; further discussion is available in supplementary text, results].

The effects of the therapeutic food interventions on the representation of metabolic pathways in the gut microbiome were defined by shotgun sequencing of 331 fecal DNA samples obtained from 30 members of the Mirpur birth cohort with consistently healthy anthropometry and 15 of the 54 children enrolled in the SAM study (table S5B); these 15 children were selected according to their age (12 to 18 months) and that we had corresponding plasma metabolomic and proteomic datasets for at least two of the three time points sampled. The abundances of microbial genes that mapped to pathways in the microbial communities SEED (mcSEED) database (*12*)—related to metabolism of amino acids, carbohydrates, fermentation products, and B vitamins and related cofactors—were first defined in healthy children sampled monthly from birth to 2 years of age. A set of age-discriminatory metabolic pathways (mcSEED “subsystems” or pathway modules) was identified. The resulting sparse RF-derived model (fig. S1, A and B, and materials and methods) allowed us to assign a state of development (functional age or “maturity”) to the fecal microbiomes of the 15 children treated for SAM. Relative functional maturity was significantly correlated with MAZ, WHZ, and WAZ scores during the course of the trial (Pearson *r* and *P* values are MAZ, *r* =0.55, *P* < 0.0001; WHZ, *r* =0.30, *P* = 0.0011; WAZ, *r* = 0.23, *P* =0.013). At enrollment, and just before administration of therapeutic foods, children with SAM had more immature microbiomes [one-way analysis of variance (ANOVA) *P* = 0.0002; Dunnett’s multiple comparisons test for healthy versus SAM adjusted *P* values at the two time points, 0.027 and 0.0001, respectively]. There was a statistically significant improvement in functional maturity from initiation of therapeutic food treatment to discharge, and at 1 and 6 months after discharge (Tukey’s multiple comparisons test; adjusted *P* values = 0.039, 0.0028, and 0.025, respectively). However, this improvement was not sustained at later time points (fig. S1D). Comparing the relative abundances of the 30 most age-discriminatory pathways at six time points revealed that the SAM microbiome had significantly reduced representation of (i) amino acid metabolic pathways, including those involved in isoleucine, leucine, valine biosynthesis, and uptake; (ii) several carbohydrate utilization pathways (arabinose and arabinosides, rhamnose and rhamnogalacturonan, and sialic acid); and (iii) multiple pathways involved in B-vitamin metabolism, including “niacin/NADP (nicotinamide adenine dinucleo-tide phosphate) biosynthesis” (fig. S1E and table S5C). The observed underrepresentation of age-discriminatory OTUs and metabolic pathways in the gut communities of children with post-SAM MAM provided the rationale for developing a pipeline to test complementary food ingredients for their ability to repair this immaturity.

## Screening complementary food ingredients

Nine age-discriminatory bacterial strains were cultured from the fecal microbiota of three healthy children, aged 6 to 23 months, who lived in Mirpur, and genomes of these isolates were sequenced (table S6, A and C). Seven of the nine isolates had V4-16*S* rDNA sequences that corresponded to age-discriminatory OTUs whose representation is associated with the period of complementary food consumption (“weaning-phase” OTUs) (fig. S2A), whereas two, *Bifidobacterium longum* subsp. *infantis* and *Bifidobacterium breve*, are most prominent during the period of exclusive, predominant milk feeding (fig. S2A) (*13*). OTUs representing seven of the nine cultured strains were significantly depleted in the fecal microbiota of Bangladeshi children with SAM before treatment (table S7 and fig. S3). Seven additional age-discriminatory strains were cultured from the immature fecal microbiota of a 24-month-old child with SAM enrolled in the same study as the subcohort shown in Fig. 1 (table S6, A and C). Together, the consortium of 16 strains represented OTUs that directly matched 65.6 ± 22.8% (mean ± SD) of V4-16S rDNA sequencing reads generated from 1039 fecal samples collected from 53 healthy members of the Mirpur birth cohort during their first 2 postnatal years, and74.2±25.2%ofthe reads produced from fecal samples obtained from 38 children with SAM (table S7).

To identify complementary foods that selectively increase the representation of weaning-phase age-discriminatory strains deficient in immature SAM-associated microbiota, we colonized 5-week-old, germ-free C57Bl/6J mice with the consortium of cultured, sequenced bacterial strains. After colonization, an 8-week period of diet “oscillations” was initiated (fig. S2B). We incorporated 12 complementary food ingredients commonly consumed in Mirpur (*6*) into 14 different diets using a random sampling strategy (table S8, A to E, and materials and methods). The composition of these complementary food combinations (CFCs) and their order of administration to mice were based on considerations described in the legend to fig. S2, B and C.

Spearman’s rank correlation coefficients were calculated between the relative abundances of the 14 bacterial strains that colonized mice and levels of complementary food ingredients in the 14 CFCs tested (fig. S2D and table S9A). Chickpea and banana had strong positive correlations with the greatest number of strains representing weaning-phase age-discriminatory OTUs. Tilapia had a narrower range of significant positive effects (fig. S2D). Chickpea, banana, and tilapia also had significant negative correlations with levels of the preweaning, milk-adapted *B. longum* subsp. *infantis* isolate. A sobering observation was that a number of complementary food ingredients typically represented in diets consumed by 18-month-old children living in Mirpur had significant negative correlations with six of the weaning-phase age-discriminatory strains, including rice, milk powder, potato, spinach, and sweet pumpkin (fig. S2D). Rice gruel with milk is the most common first complementary food given to Bangladeshi children (*14*). Moreover, egg, which is included in a number of regimens for nutritional rehabilitation of children with acute malnutrition (*15*), was negatively correlated with the abundance of two weaning-phase strains, *Dorea formicigenerans* and *Blautia luti.*

## Testing an initial MDCF prototype

Khichuri-halwa (KH) is a therapeutic food commonly administered together with milk-suji (MS) to Mirpur children with SAM. A previous study documented the inability of this intervention to repair gut microbiota immaturity (*2*). We prepared a diet that mimicked MS and KH (MS/KH) (table S8, D and E); 7 of its 16 ingredients are commonly consumed complementary foods that had little, if any, effect on the representation of weaning-phase age-discriminatory strains (rice, red lentils, potato, pumpkin, spinach, whole-wheat flour, and powdered milk) (fig. S2D). The effects of MS/KH on members of the 14-member consortium and the host were compared with those produced by an initial MDCF prototype that contained chickpeas, banana, and tilapia (table S9B). Five-week-old germ-free C57Bl/6J mice colonized with the consortium were monotonously fed either of the two diets ad libitum for 25 days.

### Microbial community responses

**Fig. 3:**
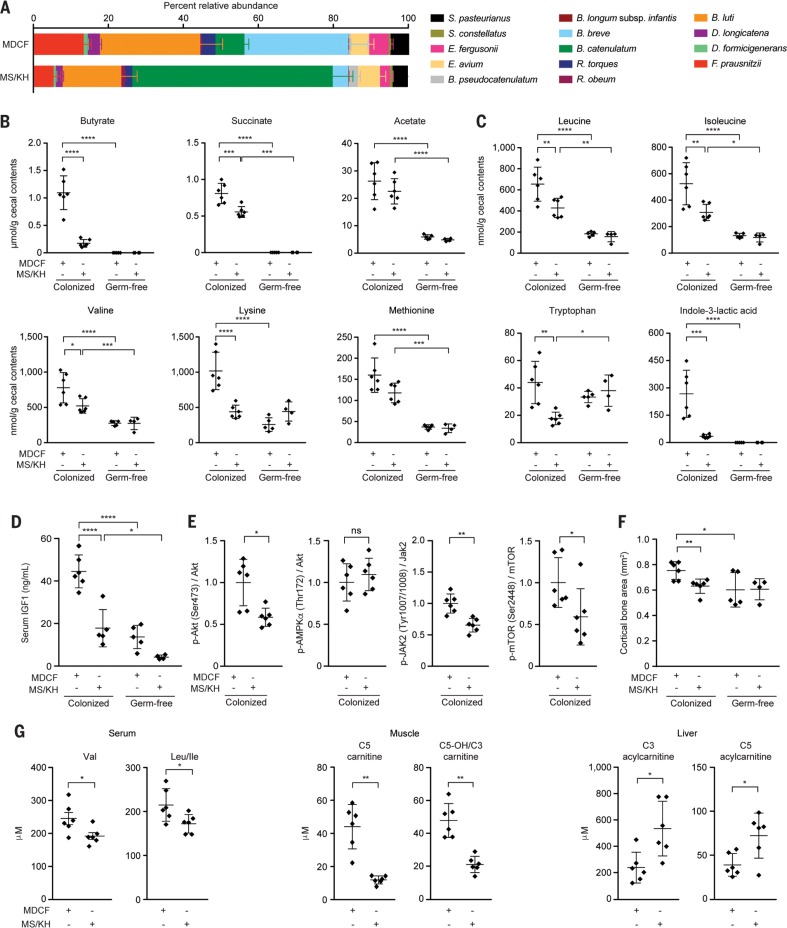
**Comparison of microbial community and host effects of an initial MDCF prototype versus MS/KH.** Separate groups of germ-free mice or animals colonized with the defined consortium of 14 bacterial strains were fed the two diets, monotonously, for 25 days, after which time they were euthanized, and cecal contents were analyzed. (**A**) The relative abundances of strains in the cecal microbiota of colonized mice. Mean values ± SD shown. (**B** and **C**)Diet- and colonization-dependent effects on (B) cecal levels of short chain fatty acids and (C) essential amino acids plus the tryptophan metabolite, indole 3-lactic acid. Each dot represents a sample from a mouse in the indicated treatment group. Mean values ± SD are shown. ***P < 0.001; ****P <0.0001[2-way ANOVA followed by Tukey’s multiple comparisons test for (A) to (C)]. (**D**) Diet- and colonization-dependent effects on serum IGF-1 levels. (**E**) Effects of diet on levels of liver proteins involved in IGF-1 signaling and IGF-1 production. Levels of phosphorylated proteins were normalized to the total amount of the corresponding nonphosphorylated protein or to glyceraldehyde-3-phosphate dehydrogenase (GAPDH). (**F**) Effect of diet and colonization status on the cortical thickness of femoral bone. (**G**) Effects of diet in colonized gnotobiotic mice on branched-chain amino acids in serum and acylcarnitines in muscle and liver. [C5-OH/C3 are isobars that are not resolved through flow injection MS/MS. C5-OH is a mix of 3-hydroxy-2-methylbutyryl carnitine (derived from the classical isoleucine catabolic intermediate 3-hydroxy-2-methylbutyryl CoA) and 3-hydroxyisovaleryl carnitine (a noncanonical leucine metabolite)]. For (D) to (G), mean values ± SD are shown. ns, not significant. *P <0.05; **P < 0.01; ****P < 0.0001 for (D) to (G) (Mann-Whitney test).

Community profiling by means of short read shotgun sequencing (COPRO-seq) of cecal DNA revealed that compared with MS/KH, consumption of the MDCF prototype resulted in significantly higher relative abundances of a number of weaning-phase age-discriminatory taxa, including *Faecalibacterium prausnitzii*, *Dorea longicatena*, and *B. luti* (*P* <0.01;Mann-Whitney test) (Fig. 3A and table S9B). This prototype did not promote the fitness of the SAM donor-derived strains, with the exception of *Escherichia fergusonii*.

We used targeted MS to quantify cecal levels of carbohydrates, short-chain fatty acids, plus amino acids and their catabolites (table S10, A to D). Germ-free animals served as reference controls to define levels of cecal nutrients that, by inference, would be available for bacterial utilization in the different diet contexts. There were several noteworthy findings: (i) Levels of butyrate and succinate were significantly higher in colonized animals consuming MDCF compared with MS/KH (Fig. 3B and table S10B). (ii) There were no statistically significant diet-associated differences in levels of any of the amino acids measured in germ-free animals, but when compared with their colonized MS/KH–fed counterparts, colonized MDCF-consuming animals had significantly elevated cecal levels of six amino acids classified as essential in humans (the three branched-chain amino acids leucine, isoleucine, and valine plus phenylalanine and tryptophan) (Fig. 3C and table S10C). And (iii) two tryptophan-derived microbial metabolites that play important roles in suppressing inflammation and are neuroprotective, 3-hydroxyanthranillic acid (3-HAA) and indole-3-lactic acid (*16*–*21*), were significantly elevated in colonized animals fed MDCF compared with their MS/KH–treated counterparts (table S10D).

Findings from RNA-sequencing (RNA-seq) analysis of the transcriptional responses of community members to the two diets based on Kyoto Encyclopedia of Genes and Genomes (KEGG)– and mcSEED-derived annotations of the 40,735 predicted protein-coding genes present in consortium members are described in tables S9C and S11, A to C, and supplementary text, results, and in silico predictions of their ability to produce, use, and/or share nutrients are provided in table S6, D and E. For example, community-level analysis revealed specific members manifested MDCF-associated increases in expression of genes involved in biosynthesis of the essential amino acids, including branched-chain amino acids (*Ruminococcus obeum* and *Ruminococcus torques*), and generation of aromatic amino acid metabolites (*R. obeum*, *R. torques*, and *F. prausnitzii*)(table S11C, ii).

### Host effects

Serum levels of IGF-1 were significantly higher in colonized mice that consumed the initial MDCF prototype compared with those that consumed MS/KH. This effect was diet- and colonization-dependent, with germ-free animals exhibiting significantly lower levels of IGF-1 in both diet contexts (Fig. 3D). Serum insulin levels were also higher in colonized animals that consumed MDCF compared with MS/KH [800.7 ± 302.9 ng/mL (mean ± SD) versus 518.7 ± 135.1 ng/mL, respectively; *P* =0.06;unpaired *t* test].

IGF-1 binding to its receptor tyrosine kinase, IGF-1R, affects a variety of signal transduction pathways, including one involving the serine/threonine kinase Akt/PKB, phosphatidylinositol-3 kinase (PI3K), and the mammalian target of rapamycin (mTOR). Absorption of several amino acids from the gut—notably, branched-chain amino acids and tryptophan—leads to activation of mTOR (*22*). Colonized animals fed MDCF had significantly higher levels of hepatic phosphoSer473-Akt, which is consistent with activation of Akt by IGF-1 signaling through the PI-3K pathway (Fig. 3E). Levels of phospho–AMPK (5′ adeno-sine monophosphate-activated protein kinase) were not significantly affected by diet (Fig. 3E), suggesting that Akt phosphorylation is not caused indirectly by altered hepatic energy status. Phosphorylation of hepatic Jak 2 (Tyr1007/1008) and mTOR (Ser2448), which are involved in IGF-1 production, was significantly increased in colonized mice consuming MDCF (Fig. 3E), whereas phosphorylation of STAT5, also implicated in IGF-1 production, was not significantly altered.

Previous studies of adult germ-free mice reported increases in serum IGF-1 after their colonization with gut microbiota from conventionally raised mice; increased IGF-1 levels were also associated with increased bone formation (*23*, *24*). Micro-computed tomography (mCT) of mouse femurs revealed a significant increase in femoral cortical bone area in MDCF-fed animals; the effect was both diet- and microbiota-dependent (Fig. 3F).

We used targeted MS to quantify levels of amino acids, acyl–coenzyme As (acylCoAs), acylcarnitines, and organic acids in serum, liver, and gastrocnemius muscle (table S12). Products of nonoxidative metabolism of glucose and pyruvate (lactate from glycolysis and alanine from transamination of pyruvate, respectively) were significantly lower in mice fed MDCF compared with mice fed MS/KH; this was true for alanine in serum, skeletal muscle, and liver and for lactate in liver (table S12, A to C and H). Oxidative metabolism of glucose is associated with nutritionally replete, anabolic conditions. These findings are consistent with the observed elevations of the anabolic hormone IGF-1 in MDCF-fed compared with MS/KH–fed mice. MDCF-fed mice had significantly higher circulating levels of branched-chain amino acids than those of their MS/KH–fed counterparts (Fig. 3G and table S12, A to C). Skeletal muscle C5 carnitine and the closely related metabolite C5-OH/C3 carnitine were significantly higher in animals consuming MDCF (Fig. 3G and table S12F). In liver, C3 and C5 acylcarnitines were significantly lower in MDCF-treated mice (Fig. 3G and table S12E), suggesting that the more nutritionally replete state associated with MDCF may act to limit branched-chain amino acid oxidation in this tissue.

## Testing additional MDCF prototypes in gnotobiotic mice

Incorporating tilapia into MDCF prototypes poses several problems: Its organoleptic properties are not desirable, and its cost is higher than commonly consumed plant-based sources of protein. To identify alternatives to tilapia, we selected an additional 16 plant-derived complementary food ingredients with varied levels and quality of protein (*25*) that are culturally acceptable, affordable, and readily available in Bangladesh (fig. S4A and table S13, A and B). Their effects were tested in gnotobiotic mice colonized with a defined, expanded consortium of 18 age- and growth-discriminatory bacterial strains (table S6A). We generated 48 mouse diets by supplementing a prototypic base diet representative of that consumed by 18-month-old children living in Mirpur (Mirpur-18), with each of the individual ingredients incorporated at three different concentrations (fig. S4A and table S13A). The results revealed that in this defined community context, peanut flour had the greatest effect on the largest number of targeted weaning-phase age-discriminatory taxa, followed by chickpea flour (fig. S4B and table S13C). Soy flour, which promoted the representation of two of these taxa, had the second-highest percentage protein after peanut flour (fig. S4A), and its protein quality was among the highest of the ingredients tested (table S13B). On the basis of these observations, we chose soy and peanut flours as replacements for tilapia in subsequent MDCF formulations.

**Fig. 4:**
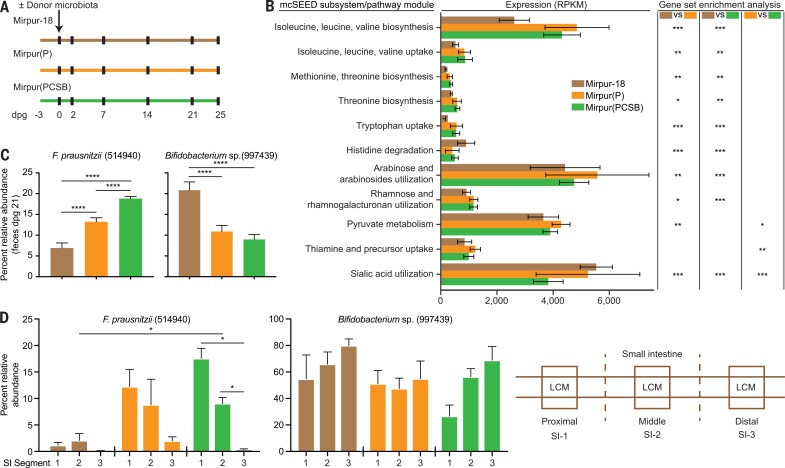
**Effects of Mirpur-18 diet supplementation on a post-SAM MAM donor microbiota transplanted into gnotobiotic mice.** (**A**) Experimental design. dpg, days post gavage of the donor microbiota; Mirpur(P), Mirpur-18 supplemented with peanut flour; Mirpur(PCSB), Mirpur-18 supplemented with peanut flour, chickpea flour, soy flour, and banana. (**B**) Expression of microbial mcSEED metabolic pathway/modules in the ceca of gnotobiotic mouse recipients of the post-SAM MAM donor gut community as a function of diet treatment. *P < 0.05; **P < 0.001; ***P < 0.0001 (statistical comparisons indicate results of gene set enrichment analysis expression on a per-gene basis across the indicated mcSEED subsystem/pathway module; all *P* values are FDR-adjusted). (**C**) Effects of supplementing Mirpur-18 with one or all four complementary food ingredients on the relative abundances of a weaning-phase– and a milk-phase–associated taxon in feces obtained at dpg 21 (one-way ANOVA followed by Tukey’s multiple comparisons test). (**D**) Relative abundances of the two taxa in mucosa harvested by means of LCM from the proximal, middle, and distal thirds of the small intestine. (Right) Schematic of locations in the small intestine where LCM was performed. The same color code for diets is used in (A) to (D). *P < 0.05; **P < 0.01; ****P < 0.0001 (Mann-Whitney test).

We reasoned that by transplanting a representative immature intact microbiota into young, germ-free mice, we could investigate whether gut health (defined by relative abundances of community members, expression of microbial genes in mcSEED metabolic pathways, and biomarkers and mediators of gut barrier function) was improved by supplementing the Mirpur-18 diet with one or more complementary food ingredients that target weaning-phase age-discriminatory taxa. Fifteen fecal samples from 12 different children, obtained during or after treatment for SAM, were screened in gnotobiotic mice to identify samples containing the greatest number of transmissible weaning-phase age-discriminatory taxa and to assess their response to supplementation of Mirpur-18 (table S14A). We selected a sample obtained from a donor (PS.064) who had post-SAM MAM; in addition to the successful transmission of targeted taxa, 88.7 ± 1.3% (mean ± SD) of the recipient animals’ gut communities consisted of OTUs that were detected at >0.1% relative abundance in the donor sample (table S14B). Three groups of mice were colonized with this microbiota and monotonously fed one of three diets: unsupplemented Mirpur-18, Mirpur-18 supplemented with peanut flour [Mirpur(P)], or Mirpur-18 supplemented with four of the lead ingredients [Mirpur(PCSB), with peanut flour, chickpea flour, soy flour, and banana] (Fig. 4A and table S15A). Three control groups were maintained as germ-free; each group was fed one of the three diets.

We characterized the effects of diet supplementation on cecal and serum levels of metabolites as well as on expression of genes in various microbial metabolic pathways (tables S15, B, D, and E, and S16 and supplementary text, results).

Eighteen mcSEED pathway modules involved in amino acid metabolism were significantly up-regulated in the cecal microbiomes of mice consuming Mirpur(PCSB) or Mirpur(P) compared with those consuming Mirpur-18, with the most up-regulated being “isoleucine, leucine, valine biosynthesis” [other age-discriminatory mcSEED pathway modules that showed significantly lower abundances in the fecal microbiomes of Bangladeshi children with SAM and whose expression was increased by Mirpur(PCSB) or Mirpur(P) in gnotobiotic mice are provided in Fig. 4B and fig. S1E]. Serum levels of a product of branched-chain amino acid metabolism, C5:1-acylcarnitine, were significantly higher in mice consuming Mirpur(PCSB) compared with un-supplemented Mirpur-18 (0.148 ± 0.015 versus 0.086 ± 0.0098 mM, respectively; *P* = 0.014, un-paired *t* test). Findings from mass spectrometric analysis of cecal contents, isolation, and comparative genomic analysis of an *F. prausnitzii* strain prominently represented in the transplanted community, and characterization of the in vivo transcriptional responses of this strain to the different diets, are described in table S15F and supplementary text results.

### Gut mucosal barrier function

Epithelium and overlying mucus from the proximal, middle, and distal thirds of the small intestine were recovered with laser capture microdissection (LCM) (Fig. 4D). Listed in table S15C are the 30 most abundant OTUs identified by means of V4-16*S* rDNA analysis of LCM mucosal DNA obtained from the different small intestinal segments within a given diet group and between similarly positioned segments across the different diet treatments. For example, Mirpur(PCSB) produced a statistically significant increase in the relative abundance of *F. prausnitzii* in the proximal two-thirds of the small intestine, without significantly affecting the proportional representation of a milk-associated age-discriminatory *Bifidobacteria* OTU (Fig. 4, C and D).

Gene expression was characterized in the jejunal mucosa (Fig. 4D, SI-2 segment) recovered by LCM from mice belonging to all six treatment groups. Significant differences in expression were categorized based on enriched Gene Ontology (GO) terms for “Molecular Function.” In colonized mice, Mirpur(P) and Mirpur(PCSB) significantly up-regulated genes assigned to “cadherin binding” (GO: 0045296) and “cell adhesion molecule binding” (GO: 0050839) compared with Mirpur-18 (table S17A). The diet effect was colonization-dependent; there were no significant differences in expression of these genes or these GO categories in germ-free mice consuming supplemented versus unsupplemented diets (table S17). (Further discussion is available in the supplementary text, results, and histochemical and immunohistochemical analyses of tissue sections prepared along the length of the small intestines of these mice are provided in fig. S6). On the basis of its effects on microbial community organismal composition, gene expression, and gut barrier function, we deemed Mipur-18 supplemented with the four lead complementary foods [Mirpur(PCSB)] superior to that supplemented with just peanut flour [Mirpur(P)].

## Characterizing MDCF prototypes in gnotobiotic piglets

We examined the effects of MDCF prototypes in a second host species whose physiology and metabolism are more similar to that of humans. Gnotobiotic piglets provide an attractive model for these purposes; piglets manifest rapid growth rates in the weeks after birth (*26*), and methods for conducting experiments with gnotobiotic piglets have been described (*27*).

On the basis of the results from the gnotobiotic mouse studies, we designed two MDCF prototypes. One prototype was formulated to be analogous to Mirpur-18, which contains milk powder; this prototype was supplemented with peanut flour, chickpea flour, soy flour, and banana [MDCF(PCSB)]. The other diet lacked milk powder and was supplemented with just chickpea flour and soy flour [MDCF(CS)]. The two MDCFs were isocaloric, matched in lipid levels and total protein content (with equivalent representation of amino acids), and also met current ready-to-use therapeutic food guidelines for children with respect to macro- and micronutrient content (table S18A) (28).

**Fig. 5:**
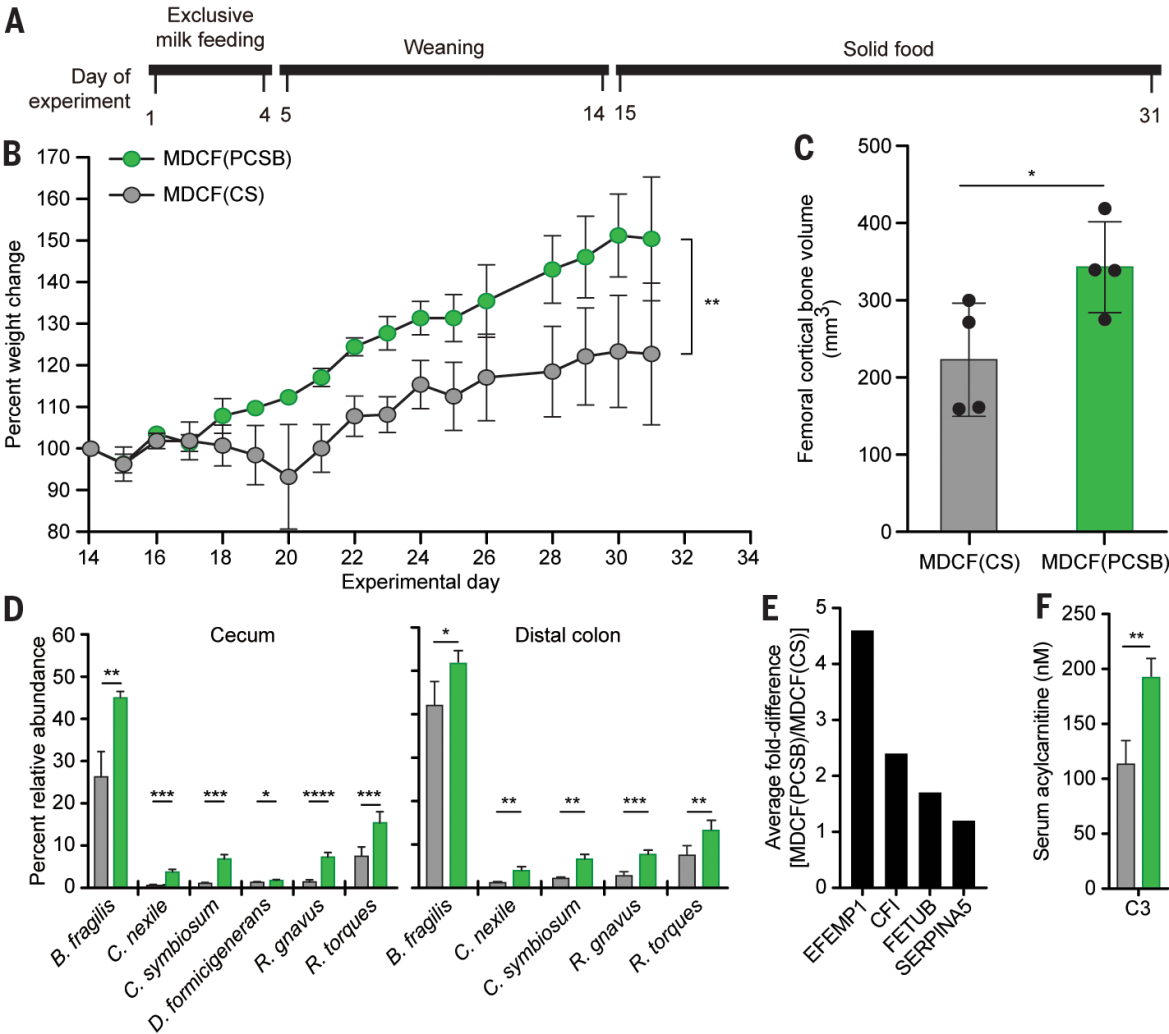
**Effects of two different MDCF prototypes in gnotobiotic piglets.** (**A**) Experimental design. (**B**) Weight gain in piglets weaned onto isocaloric MDCF prototypes containing either peanut flour, chickpea flour, soy flour, and banana [MDCF(PCSB)] or chickpea and soy flours [MDCF(CS)]. (**C**) mCT of femoral bone obtained at euthanasia. (**D**) Effects of the MDCFs on the relative abundances of community members in cecal and distal colonic contents. (**E**) Examples of serum proteins with significantly different post-treatment levels between the two diet groups. (**F**) Effect of diet on serum C3 acylcarnitine levels. Mean values ± SD are plotted. *P < 0.05; **P < 0.01; ***P < 0.005, ****P < 0.001 [two-way ANOVA in (B), unpaired t test in (C), (D), and (F)]. The color code provided in (B) also applies to (C), (D), and (F).

Four-day-old germ-free piglets fed a sow milk– based formula were colonized with a 14-member consortium of bacterial strains that consisted of the same nine Bangladeshi age-discriminatory strains used for the diet oscillation experiments described in fig. S2, plus five weaning-phase age-discriminatory strains cultured from Malawian children (table S6B). In an earlier study, several members of this consortium (*B. longum*, *F. prausnitzii*, *Clostridium*, *Ruminococcus gnavus*, and *D. formicigenerans*) had been classified as growth-discriminatory by means of a RF-based analysis of their representation in gnotobiotic mouse recipients of healthy and undernourished donor microbiota and the animals’ weight and lean body mass gain phenotypes (*4*). After gavage, the two groups of piglets were weaned over the course of 10 days (supplementary materials, materials and methods) onto one or the other irradiated MDCF prototypes, which they consumed ad libitum for the remainder of the experiment (*n* = 4 piglets/treatment arm) (Fig. 5A). Animals were euthanized on day 31 after a 6-hour fast.

Piglets fed MDCF(PCSB) exhibited significantly greater weight gain than those receiving MDCF(CS) (Fig. 5B). Micro-computed tomography of their femurs revealed that they also had significantly greater cortical bone volume (Fig. 5C). COPRO-seq analysis disclosed that pig-lets fed MDCF(PCSB) had significantly higher relative abundances of *C. symbiosum*, *R. gnavus*, *D. formicigenerans*, *R. torques*, and *Bacteroides fragilis* in their cecum and distal colon compared with those of piglets consuming MDCF(CS) (Fig. 5D and table S18B); all are weaning-phase age-discriminatory strains, and the former three were, as noted above, also defined as growth-discriminatory. Conversely, the relative abundances of three members of Bifidobacteria (including two milk-associated age-discriminatory strains, *B. breve* and *B. longum* subsp. *infantis*)were significantly higher in the ceca and distal colons of piglets fed MDCF(CS) (table S18B). These findings led us to conclude that MDCF(PCSB) promoted a more weaning-phase–like (mature) community configuration than MDCF(CS). (genome annotations, microbial RNA-seq, and targeted MS analyses of cecal metabolites are provided in tables S18, C and D, and S19A and supplementary text, results).

The effects of the two MDCF prototypes on host biology were defined by means of MS-based serum metabolomic and proteomic analyses (tables S19 and S20). Notable findings included significant increases in levels of tryptophan, methionine, and C3-acylcarnitine with MDCF(PCSB) as well as changes produced in the serum proteome that are shared with children in the SAM trial (Fig. 5, E and F, and supplementary text, results).

## Testing MDCFs in Bangladeshi children with MAM

**Fig. 6:**
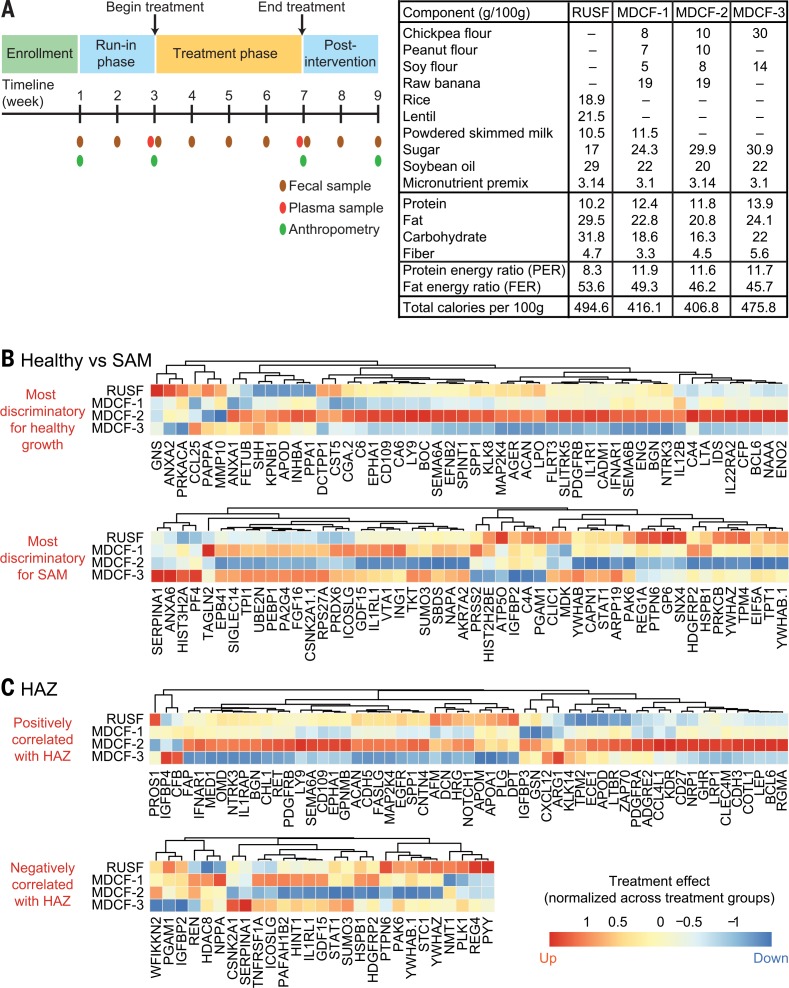
**Comparing the effects of MDCF formulations on the health status of Bangladeshi children with MAM.** (**A**) Study design and composition of diets. Total carbohydrate includes all components except added sugar. (**B**) Quantitative proteomic analysis of the average fold-change, per treatment group, in the abundances of the 50 plasma proteins most discriminatory for healthy growth and the 50 plasma proteins most discriminatory for SAM (protein abundance is column-normalized across treatment groups). (**C**) Average fold-change in abundances of plasma proteins that significantly positively or negatively correlate with HAZ [absolute value of Pearson correlation > 0.25, FDR-corrected *P* value < 0.05; abundance is column-normalized as in (B)].

To assess the degree to which results obtained from the gnotobiotic mouse and piglet models translate to humans, we performed a pilot randomized, double-blind controlled feeding study of the effects of three MDCF formulations. The formulations (MDCF-1, -2, and -3) were designed to be similar in protein energy ratio and fat energy ratio and provide 250 kcal/day (divided over two servings). MDCF-2 contained all four lead ingredients (chickpea flour, soy flour, peanut flour, and banana) at higher concentrations than in MDCF-1. MDCF-3 contained two lead ingredients (chickpea flour and soy flour). A rice- and lentil-based ready-to-use supplementary food (RUSF), included as a control arm, lacked all four ingredients but was otherwise similar in energy density, protein energy ratio, fat energy ratio, and macro- and micronutrient content to those of the MDCFs (Fig. 6A). Milk powder was included in MDCF-1 and RUSF. All formulations were supplemented with a micronutrient mixture designed to provide 70% of the recommended daily allowances for 12- to 18-month-old children. The formulations were produced locally and tested for organoleptic acceptability before initiating the trial (table S21A).

Children from Mirpur with MAM and no prior history of SAM were enrolled (mean age at enrollment, 15.2 ± 2.1 months, mean WHZ –2.3 ± 0.3). Participants were randomized into one of the four treatment groups (14 to 17 children per group) and received 4 weeks of twice-daily feeding under supervision at the study center, preceded and followed by 2 weeks of observation and sample collection. Mothers were encouraged to continue their normal breastfeeding pactices throughout the study (Fig. 6A). There were no significant differences in the mean daily amount of each MDCF or RUSF consumed per child or in the mean incidence of morbidity across the four treatment groups (table S21, B to D). All three MDCFs and the RUSF control improved WHZ scores [–1.9 ± 0.5 (mean ± SD) at the completion of intervention compared with –2.2 ± 0.4 at the start of intervention; *n* =63 children, *P* = 2.06 × 10^−^11 for all groups combined, paired *t* test]. There were no statistically significant differences between the four interventions in the change in WHZ (*P* = 0.31, oneway ANOVA). Despite the small group size and the short length of the study, there were significant differences in treatment effects on another anthropometric indicator, with MDCF-2 producing a significantly greater increase in mid-upper arm circumference (MUAC) than MDCF-3 (oneway ANOVA, *P* = 0.022; with Tukey’s multiple comparisons test, *P* = 0.017) (table S21E).

### Effects on biological state

To contextualize the biological effects of the dietary interventions, we performed quantitative proteomics (SOMAscan) on plasma collected from 21 12- to 24-month-old Mirpur children with healthy growth phenotypes (mean age, 19.2 ± 5.1 months; WHZ, 0.08 ± 0.58; HAZ, –0.41 ± 0.56, WAZ, –0.12 ± 0.60) and 30 children with SAM before treatment (Fig. 1A, B1 sample; WHZ < –3; mean age 15.2 ± 5.1 months) [metadata associated with the healthy, SAM, and MAM (MDCF trial) cohorts are provided in table S22]. We rank-ordered all detected proteins according to fold differences in their abundances in plasma collected from healthy children compared with children with untreated SAM. The top 50 most differentially abundant proteins (*P* <10^−^7;Rpackage “limma”) that were significantly higher in healthy children were designated “healthy growth-discriminatory,” whereas the top 50 differentially abundant proteins that were significantly higher in children with SAM were designated “SAM-discriminatory” (table S23A). We next compared the mean difference for each protein in the pre- versus post-intervention plasma samples for all children in each MDCF/RUSF treatment group. Proteins were then ranked according to the fold differences of the pre- versus post-treatment levels in each of the four groups (table S23B), and these treatment effects were mapped onto the 50 most healthy growth-discriminatory and 50 most SAM-discriminatory proteins. Strikingly, MDCF-2 elicited a biological response characterized by a marked shift in the plasma proteome toward that of healthy children and away from that of children with SAM; MDCF-2 increased the abundance of proteins that are higher in plasma from healthy children and reduced the levels of proteins elevated in SAM plasma samples (Fig. 6B).

Aggregating proteomic datasets from the combined cohort of 113 children with SAM, MAM, and healthy growth phenotypes for whom plasma samples were available, we identified a total of 27 plasma proteins that were significantly positively correlated with HAZ and 57 plasma proteins that were significantly negatively correlated with HAZ [absolute value of Pearson correlation > 0.25, false discovery rate (FDR)– corrected *P* value < 0.05]. Among the treatments, MDCF-2 was distinctive in its ability to increase the abundances of a broad range of proteins positively correlated with HAZ, including the major IGF-1 binding protein IGFBP-3, growth hormone receptor (GHR), and leptin (LEP) (Fig. 6C). Growth differentiation factor 15 (GDF15) was reduced after 4 weeks of dietary supplementation with MDCF-2 (Fig. 6C). This transforming growth factor–b superfamily member, which was negatively correlated with HAZ, has been implicated in the anorexia and muscle wasting associated with cancer and with chronic heart failure in children; it was elevated in children with SAM and positively correlated with their lipolytic biomarkers NEFA and ketones (supplementary text, results). Peptide YY, an enteroendocrine cell product elevated in SAM plasma that reduces appetite and negatively correlated with HAZ, was also decreased by MDCF-2.

**Fig. 7:**
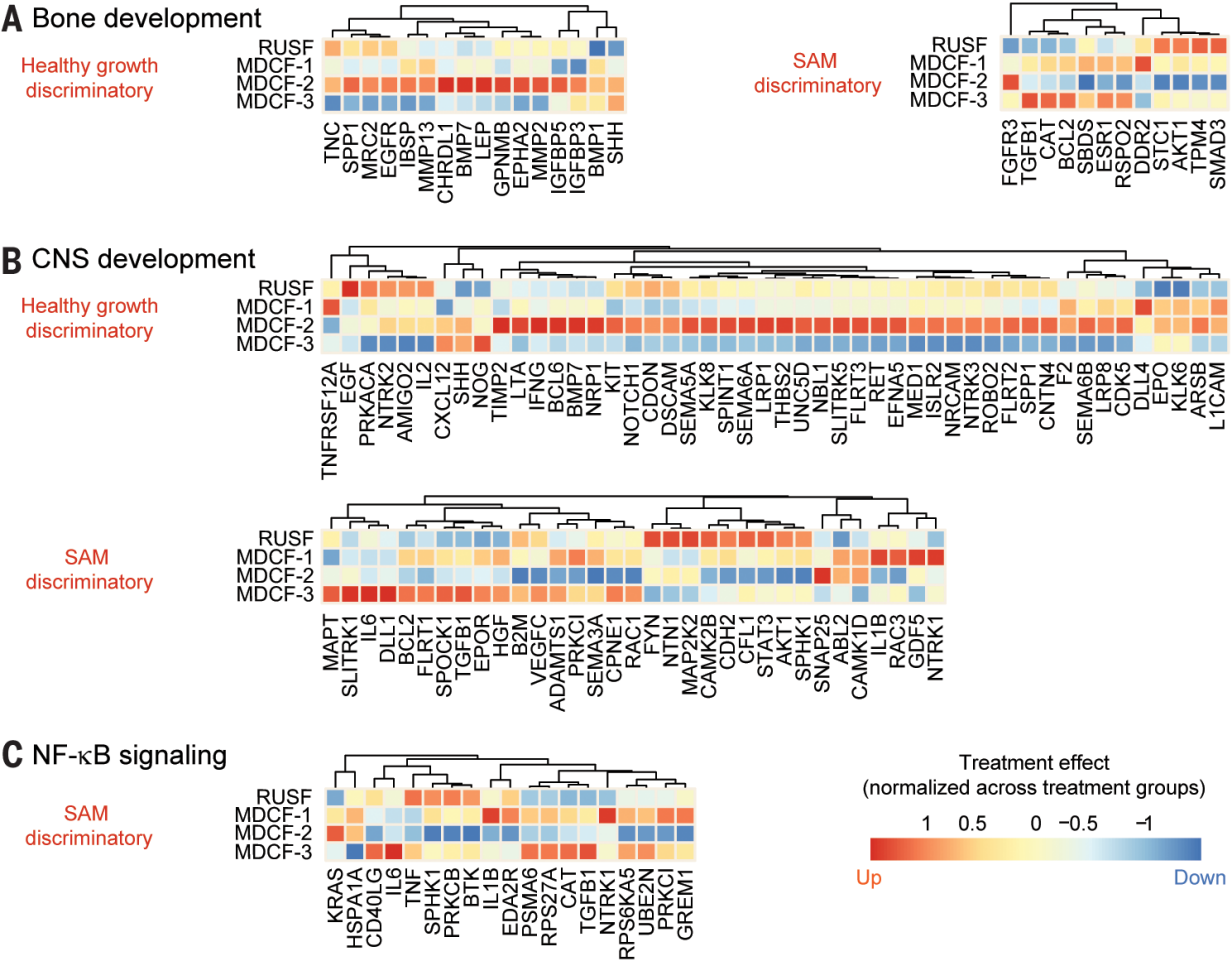
**The effects of different MDCF formulations on biomarkers and mediators of bone and CNS development, plus NF-kB signaling**. (**A** to **C**) Average fold-change (normalized across treatment groups) in the abundances of plasma proteins belonging to GO categories related to (A) bone, (B) CNS development, and (C) agonists and components of the NF-ĸB signaling pathway. Proteins in the GO category that were significantly higher in the plasma of healthy compared with SAM children (fold-difference >30%; FDR-adjusted *P* value < 0.05) are labeled “healthy growth-discriminatory,” whereas those higher in SAM compared with healthy children (fold-difference >30%; FDR-adjusted *P* value < 0.05) are labeled “SAM-discriminatory.” Levels of multiple “healthy growth-discriminatory” proteins associated with (A) GO processes “osteoblast differentiation” and “ossification”, and (B) the GO process “CNS development” are enhanced by MDCF-2 treatment while (C) NF-kB signaling is suppressed.

We identified GO terms that were enriched among the group of treatment-responsive proteins and ranked them according to the *P* value of their enrichment (table S23C). Proteins belonging to GO terms significantly higher in healthy compared with SAM plasma samples were deemed “healthy growth-discriminatory,” whereas those that were significantly higher in SAM were deemed “SAM-discriminatory” (fold-difference >30%; FDR-adjusted *P* value <0.05). This analysis revealed multiple healthy growth-discriminatory proteins associated with GO processes “osteoblast differentiation” and “ossification” that were increased by supplementation with MDCF-2 (Fig. 7A and table S23C). Examples include key markers or mediators of osteoblast differentiation [osteopontin (SPP1), bone sialoprotein 2 (IBSP), and bone morphogenetic protein 7 (BMP7)] as well as matrix metalloproteases (MMP-2 and MMP-13) involved in terminal differentiation of osteoblasts into osteocytes and bone mineralization.

A number of plasma proteins categorized under the GO process “CNS development,” including those involved in axon guidance and neuronal differentiation, were also affected by MDCF-2 supplementation. Levels of the SAM-discriminatory semaphorin SEMA3A, a potent inhibitor of axonal growth, decreased with this treatment, whereas healthy growth-discriminatory semaphorins (SEMA5A, SEMA6A, and SEMA6B) increased (Fig. 7B). Other healthy growth-discriminatory proteins whose abundances increased with MDCF-2 included receptors for neurotrophin (NTRK2 and NTRK3) plus various axonal guidance proteins [netrin (UNC5D), ephrin A5 (EFNA5), roundabout homolog 2 (ROBO2), and SLIT and NTRK-like protein 5 (SLITRK5)] (Fig. 7B). Expression of a number of neurotrophic proteins belonging to these families has been reported to be influenced by nutrient availability in primates (*29*).

Compared with healthy children, the plasma proteome of children with SAM was characterized by elevated levels of acute phase proteins [such as C-reactive protein (CRP) or interleukin-6 (IL-6)] and inflammatory mediators, including several agonists and components of the nuclear factor–kB (NF-kB) signaling pathway (Fig. 7C). These components include the pro-inflammatory cytokines IL-1b, tumor necrosis factor–a (TNF-a), and CD40L, plus ubiquitin-conjugating enzyme E2 N (UBE2N), which is involved in induction of NF-kB– and mitogen-activated protein kinase (MAPK)–responsive inflammatory genes (*30*). MDCF-2 supplementation was associated with reductions in the levels of all of these SAM-associated proteins (Fig. 7C).

### Effects on the microbiota

Our analysis of fecal microbiota samples revealed no significant change in the representation of enteropathogens within and across the four treatment groups (fig. S7A and table S21F). MDCF-2–induced changes in biological state were accompanied by increases in the relative abundances of several weaning-phase taxa, including OTUs assigned to *F. prausnitzii* (OTU 851865) and a *Clostridiales* sp. (OTU 338992) that are closely related to taxa ranked first and second in feature importance in the sparse Bangladeshi RF-derived model of gut microbiota maturation (fig. S7, B and C). MDCF-2 supplementation was associated with a significant decrease in *B. longum* (OTU 559527) (fig. S7B), which is ranked third in feature importance in the RF-derived model and discriminatory for a young, milk-oriented microbiota. None of the other members of the 30 OTU model showed significant changes. By contrast, MDCF-1 did not produce significant increases in any of the taxa in the model. The other two formulations were each associated with a significant change in just one member [an increase in the relative abundance of an early age-discriminatory OTU (*Streptococcus*;ranked30th)with MDCF-3 supplementation, and a decrease in another OTU (*Enterococcus faecalis*; ranked 29th) with RUSF supplementation] (table S4B).

MAZ scores were not significantly different between groups at enrollment, nor were they significantly improved by any of the formulations. Interpretation of this finding was confounded by unexpectedly high baseline microbiota maturity scores in this group of children with MAM [MAZ, –0.01 ± 1.12 (mean ± SD)] (table S22) compared with a small, previously characterized Mirpur cohort with untreated MAM (*2*). Hence, we developed an additional measure of microbiota repair (*31*). This involved a statistical analysis of covariance among bacterial taxa in the fecal microbiota of anthropometrically healthy members of a Mirpur birth cohort who had been sampled monthly over a 5-year period. Using approaches developed in the fields of econophysics and protein evolution to characterize the underlying organization of interacting systems with seemingly intractable complexity, such as financial markets, we found that the gut community in healthy children could be decomposed into a sparse unit of 15 covarying bacterial taxa termed an “ecogroup” (*31*). These ecogroup taxa include a number of age-discriminatory strains in the Bangladeshi RF-derived model (such as *B. longum*, *F. prausnitzii*, and *Prevotella copri*). We used the ecogroup to show that in addition to its effects on host biological state, MDCF-2 was also the most effective of the four treatments in reconfiguring the gut bacterial community to a mature state similar to that characteristic of healthy Bangladeshi children.

## Conclusions

We have integrated preclinical gnotobiotic animal models with human studies to understand the contributions of perturbed gut microbiota development to childhood undernutrition and to identify new microbiota-directed therapeutic approaches. We identified a set of proteins that distinguish the plasma proteomes of healthy children from those with SAM. Using these data, we have developed a supplemental food prototype, MDCF-2, that shifted the plasma proteome of children with MAM toward that of healthy individuals, including proteins involved in linear growth, bone development, neurodevelopment, and immune function. MDCF-2 is a tool for investigating, in larger studies across different populations with varying degrees of undernutrition, how repair of gut microbiota immaturity affects various facets of child growth.

## Overview of methods

### Human studies

Children aged 6 to 59 months with SAM (*n* = 343 participants) were enrolled in a study entitled “Development and field testing of ready-to-use therapeutic foods (RUTF) made of local ingredients in Bangladesh for the treatment of children with severe acute malnutrition.” The study was approved by the Ethical Review Committee at icddr, b (ClinicalTrials.gov identifier NCT01889329). Written informed consent was obtained from their parent or guardian. A subset of 54 children were included in a substudy that involved regular fecal sampling and three blood draws for up to 1 year after discharge. Children aged 12 to 18 months with MAM who were no longer exclusively breastfed (*n* =63 participants) were enrolled in a double-blind, randomized, four-group, parallel assignment interventional trial study (ClinicalTrials.gov identifier NCT03084731) approved by the Ethical Review Committee at icddr, b. Fecal and plasma samples were collected as described in the supplementary materials, materials and methods, and stored at –80°C. Samples were shipped to Washington University with associated clinical metadata and maintained in a dedicated bio-specimen repository with approval from the Washington University Human Research Protection Office.

#### Analysis of plasma samples

Methods for targeted MS-based metabolomics are described in the supplementary materials. The SOMAscan 1.3K Proteomic Assay plasma/serum kit (SomaLogic, Boulder, Colorado,) was used to measure 1305 proteins. The R package “limma” (Bioconductor) was used to analyze differential protein abundances (*32*). Spearman correlation analyses were performed between measured proteins and anthropometric scores, plasma metabolites, and the abundances of bacterial taxa in fecal samples. Plasma proteins in children with healthy growth phenotypes or SAM (before treatment) were rank-ordered according to the fold-difference in their levels between these two groups. The top 50 most differentially abundant proteins in healthy compared with SAM were designated as healthy growth-discriminatory proteins, and the top 50 most differentially abundant in SAM compared with healthy were designated as SAM-discriminatory proteins. The average fold-change for these healthy growth and SAM-discriminatory proteins was then calculated for each treatment arm in the MDCF trial (before versus after MDCF/RUSF treatment) and normalized to the mean fold-change across all four arms. Limma was used to calculate statistical significance. All 1305 proteins were mapped to all GO “Biological Processes” in the GO database (www.geneontology.org). SetRank, a gene set enrichment analysis (GSEA) algorithm (*33*), was used to identify GO Biological Processes that were significantly enriched for proteins that exhibited changes in abundance from before to after treatment with MDCF/RUSF. Enrichment was calculated by using the setRank Analysis function in the SetRank R library (parameters were use.ranks = TRUE; setPCutoff = 0.01; and fdrCutoff = 0.05). The average fold-change for each protein in the statistically significant Biological Process category was calculated for each treatment arm and normalized to the mean fold-change across all four arms. We defined proteins within the GO Biological Process as “healthy growth-discriminatory” if they were at least 30% more abundant in healthy individuals compared with those with SAM and “SAM-discriminatory” if they were at least 30% more abundant in children with SAM compared with those who were classified as healthy.

#### Characterizing human fecal microbial communities

Methods for V4-16*S* rRNA gene sequencing and data analysis, calculation of MAZ scores and functional microbiome maturity, and quantification of enteropathogen burden by means of multiplex quantitative polymerase chain reaction (qPCR) are described in the supplementary materials.

### Animal studies

#### Gnotobiotic mice

All mouse experiments were performed by using protocols approved by the Washington University Animal Studies Committee. Mice were housed in plastic flexible film gnotobiotic isolators under a 12-hour light cycle. Male germ-free C57BL/6 mice were initially weaned onto an autoclaved, low-fat, high-plant polysaccharide chow that was administered ad libitum (diet 2018S, Envigo). Animals were maintained on this diet until 3 days before the beginning of experiments involving tests of the effects of complementary food ingredients. Defined consortia of sequenced bacterial strains cultured from Bangladeshi children, or intact uncultured microbiota from donors with post-SAM MAM, were introduced by means of gavage into recipient mice at 5 weeks of age. Methods for identifying and characterizing the effects MDCF prototypes—including (i) design and preparation of diets; (ii) culturing of age-discriminatory and SAM-associated bacterial strains; (iii) shotgun sequencing of DNA isolated from serially collected fecal samples; (iv) microbial RNA-seq of cecal contents; (v) targeted MS of cecal contents, liver, gastrocnemius muscle, and serum samples for measurement of amino acids, acylcarnitines, organic acids, and acylCoAs; (vi) Western blot analysis of IGF-1 pathway components in liver; (vii) mCT of bone; and (viii) characterizing the effects of a transplanted fecal microbiome from a donor with post-SAM MAM in recipient gnotobiotic mice as a function of diet treatment by histochemical and immunohisto-chemical analysis of their intestinal segments, LCM of their small intestinal epithelium, and RNA-seq analysis of gene expression in LCM mucosa—are all described in the supplementary materials.

#### Gnotobiotic piglets

Experiments were performed under the supervision of a veterinarian by using protocols approved by the Washington University Animal Studies Committee and that followed American Veterinary Medical Association guidelines for euthanasia. The protocol for generating germ-free piglets; preparing diets, feeding, colonization, and husbandry of piglets; measurement of weight gain; mCT of femurs; and liquid chromatography– MS (LC-MS)/MS–based serum proteomics are all described in the supplementary materials.

## Supplementary Material

Effects of microbiota-directed foods in gnotobiotic animals and undernourished children

Effects of microbiota-directed foods in gnotobiotic animals and undernourished children

## Data and materials availability

V4-16*S* rDNA sequences in raw format prior to post-processing and data analysis, COPRO-seq, microbial RNA-seq, and proteomics datasets, plus shotgun sequencing datasets produced from human fecal DNA, cecal contents of gnotobiotic mice with a post-SAM MAM human donor community and cultured bacterial strains, have been deposited at the European Nucleotide Archive (ENA) under study accession no. PRJEB26419. SOMAscan-generated human plasma proteomic datasets have been deposited in the Gene Expression Omnibus (GEO) database under accession no. GSE119641. All raw mass spectra for quantification of serum proteins in gnotobiotic piglets have been deposited in the MassIVE and ProteomeXchange data repositories under accession nos. MSV000082286 (MassIVE) and PXD009534 (ProteomeXchange), with data files available at ftp://massive.ucsd.edu/MSV000082286. Fecal and plasma specimens from the SAM and MDCF studies used for the analyses described in this study were provided to Washington University under materials transfer agreements with icddr, b.

## SUPPLEMENTARY MATERIALS

science.sciencemag.org/content/365/6449/eaau4732/suppl/DC1

Figs. S1 to S7

References (*34*–*124*)
