# Supplementary material for: Effects of microbiota-directed foods in gnotobiotic animals and undernourished children
Source: Science. 2019 Jul 12;365(6449):eaau4732. doi: 10.1126/science.aau4732 (PMC6683325; doi:10.1126/science.aau4732)
Supplement: Effects of microbiota-directed foods in gnotobiotic animals and undernourished children [file Science-365-eaau4732-s001.pdf]

science.sciencemag.org/content/365/6449/eaau4732/suppl/DC1

Supplementary Material for

Effects of microbiota-directed foods in gnotobiotic animals and undernourished children

Jeanette L. Gehrig, Siddarth Venkatesh, Hao-Wei Chang, Matthew C. Hibberd, Vanderlene L. Kung, Jiye Cheng, Robert Y. Chen, Sathish Subramanian, Carrie A. Cowardin, Martin F. Meier, David O'Donnell, Michael Talcott, Larry D. Spears, Clay F. Semenkovich, Bernard Henrissat, Richard J. Giannone, Robert L. Hettich, Olga Ilkayeva, Michael Muehlbauer, Christopher B. Newgard, Christopher Sawyer, Richard D. Head, Dmitry A. Rodionov, Aleksandr A. Arzamasov, Semen A. Leyn, Andrei L. Osterman, Md Iqbal Hossain, Munirul Islam, Nuzhat Choudhury, Shafiqul Alam Sarker, Sayeeda Huq, Imteaz Mahmud, Ishita Mostafa, Mustafa Mahfuz, Michael J. Barratt, Tahmeed Ahmed, Jeffrey I. Gordon*

*Corresponding author. Email: jgordon@wustl.edu

Published 12 July 2019, *Science* **365**, eaau4732 (2019)
DOI: 10.1126/science.aau4732

This PDF file includes:

Materials and Methods
Supplementary Text
Figs. S1 to S7
References

Other Supplementary Material for this manuscript includes the following:
(available at science.sciencemag.org/content/365/6449/eaau4732/suppl/DC1)

Tables S1 to S23 as a separate Excel file

Supplementary Materials for

Effects of microbiota-directed foods in gnotobiotic animals and undernourished children

Jeanette L. Gehrig*, Siddarth Venkatesh*, Hao-Wei Chang*, Matthew C. Hibberd, Vanderlene L. Kung, Jiye Cheng, Robert Y. Chen, Sathish Subramanian, Carrie A. Cowardin, Martin F. Meier, David O'Donnell, Michael Talcott, Larry D. Spears, Clay F. Semenkovich, Bernard Henrissat, Richard J. Giannone, Robert L. Hettich, Olga Ilkayeva, Michael Muehlbauer, Christopher B. Newgard, Christopher Sawyer, Richard D. Head, Dmitry A. Rodionov, Aleksandr A. Arzamasov, Semen A. Leyn, Andrei L. Osterman, Md Iqbal Hossain, Munirul Islam, Nuzhat Choudhury, Shafiqul Alam Sarker, Sayeeda Huq, Imteaz Mahmud, Ishita Mostafa, Mustafa Mahfuz, Michael J. Barratt, Tahmeed Ahmed, Jeffrey I. Gordon[+]

*contributed equally
[+]Corresponding author. Email: jgordon@wustl.edu

This PDF file includes:

Materials and Methods
Supplementary Results
Figs. S1 to S7
Tables S1 to S23
References 34 to 124

SUPPLEMENTARY MATERIALS AND METHODS

HUMAN STUDIES

SAM trial: study population and study protocol

The human study entitled 'Development and field testing of ready-to-use therapeutic foods (RUTF) made of local ingredients in Bangladesh for the treatment of children with severe acute malnutrition' was approved by the Ethical Review Committee at icddr,b and conducted between April 2013 and December 2015 (ClinicalTrials.gov identifier: NCT01889329). The goal was to determine whether therapeutic food prototypes made from locally available food ingredients were non-inferior to a standard, commercially available RUTF used for treating children with SAM (Plumpy'Nut; Nutriset). A total of 343 children, aged 6 to 59 months, were enrolled with SAM [defined as WHZ <-3, bipedal edema, and/or a mid-upper arm circumference (MUAC) <11.5 cm]. Written informed consent was obtained from their parent or guardian. Children from urban or peri-urban areas of Dhaka were recruited at Dhaka Hospital of icddr,b, and from two clinics [Terre des Hommes (TDH) Specialized Nutritional Unit, Kurigram and Radda Maternal Child Health and Family Planning Center (MCH-FP), Mirpur].

Rehydration and antibiotic therapy were initiated for children presenting without signs or symptoms of infection other than those of diarrhea (*34*). Antibiotic treatment began with intramuscular or intravenous ampicillin 100 mg/kg/d with doses every 6 h, and gentamicin 5 mg/kg/d with doses every 12 h. If there was no evidence of septicemia after 48 h, ampicillin and gentamicin were discontinued and amoxicillin was administered orally (100 mg/kg) every 8 h for 3 more days. Children with pneumonia were treated with intravenous chloramphenicol (100 mg/kg) every 6 h for 24 h and then orally for a total of 7 days. If septicemia was suspected, ampicillin was given (200 mg/kg/day) and gentamicin was continued for 7–10 days (*34*). After

acute stabilization, entailing rehydration and a short course of antibiotics (*34*), children were transferred for follow-up treatment to the Nutrition Rehabilitation Unit (NRU) of Dhaka Hospital, TDH or Radda MCH-FP.

An appetite test was performed prior to randomization to one of the three therapeutic food arms; a rice-lentil formulation, a chickpea-containing formulation (both locally produced) (*35*), or Plumpy'Nut. Therapeutic food was provided each morning at a dose of ~200 kcal/kg/d. Children who were breast-fed continued breastfeeding. Subjects were discharged from the study upon fulfillment of the following graduation criteria: (i) edema-free WHZ ≥ -2 for those admitted with WHZ <-3 and/or edema, or (ii) MUAC ≥ 115 mm with edema-free weight gain of 15% for those admitted with a MUAC < 115 mm. Before discharge, children were treated with an anti-helminthic per national guidelines (200 mg albendazole for those aged 12-23 months; 400 mg for those greater than 24 months), and their parents received nutritional counseling.

As described in the main text and **Fig. 1A**, a subset of 54 children at the Dhaka Hospital site were enrolled in a sub-study that included regular fecal sampling and three blood draws for up to 1 year after discharge from the NRU (n = 20, Plumpy'Nut arm; n = 19, rice-lentil based therapeutic food arm; n = 15, chickpea-containing therapeutic food arm). Fecal samples were collected and within 10 minutes were transferred to liquid nitrogen pre-charged dry shippers (Taylor Wharton, CX-100) for transport back to the lab where they were stored at -80°C. EDTA plasma was prepared from blood using standard procedures and placed in a -80 ^{o}C freezer. Coded biospecimens were shipped to Washington University on dry ice where they were stored at -80°C, along with associated clinical metadata, in a dedicated biospecimen repository with approval from the Washington University Human Research Protection Office.

MDCF trial: study population and protocol

Sixty-three 12-18-month-old children diagnosed with MAM (WHZ <-2) who were no longer exclusively breastfed were enrolled in a double-blind, randomized, four group, parallel assignment interventional trial study (ClinicalTrials.gov identifier NCT03084731) conducted in Dhaka, Bangladesh and approved by the Ethical Review Committee at icddr,b. The study was designed to test the effects of three locally produced MDCF prototypes described in **Fig. 6A** and a locally produced rice-lentil-based ready-to-use supplemental food (RUSF). Linear programming was used to design the MDCF prototypes, with a target energy density of 250 kcal/50 g, and a caloric distribution of 45–55 percent from fat and 8–12 percent from protein. All diets were supplemented with a multiple micronutrient premix that provided 70% of the recommended daily allowance (RDA) for vitamins A, C, D and E, all B vitamins, calcium, copper, iron, magnesium, manganese, phosphorous, potassium and zinc. Diets were produced daily at the Food Processing Laboratory of icddr,b using a standardized production procedure to insure consistent quality of MDCFs across batches.

After obtaining informed consent, socio-demographic data were collected for all participants, and children were randomized into one of four treatment groups (n=14-17 per group). After 2 weeks on their home diet, with weekly fecal sample collection and anthropometry, children and their mothers attended a local community health clinic every morning and afternoon where each child was provided 25 g per feeding session of their assigned MDCF/RUSF. Mothers were asked not to give any food or breast milk during the 2 hours preceding the prescribed feeding session. The quantity of food consumed by each child was recorded at each visit. Mothers were instructed to continue normal breastfeeding and home-based complementary feeding practices. A blood sample was collected and EDTA-plasma was prepared from each child at the beginning of the intervention phase (week 3) and again at the end of the 4-week intervention (week 7) for targeted MS-based metabolomic and proteomic analyses. Fecal samples, together with anthropometric and morbidity data, were collected weekly from each child, including during a 2-week post-

intervention period. A separate reference cohort of thirty 12- to 24-month-old healthy children (WHZ and HAZ scores > -1) were also consented to provide a single blood and fecal sample to compare with samples collected from the children with MAM enrolled in the intervention study. All biospecimens were rapidly cryopreserved after collection (see above), coded and stored at -80°C prior to transfer to Washington University, with approval from the Washington University Human Research Protection Office.

Targeted MS-based metabolomics analysis of plasma samples
Clinical chemistry analytes, including plasma levels of glucose, lactate, triglycerides, total ketones, and non-esterified fatty acids (NEFA), were measured using a UniCel DxC600 clinical analyzer (Beckman). Reagents for the first three analytes were provided by Beckman (Brea, CA) while those for ketones and NEFA were obtained from Wako (Mountain View, CA). Amino acids, acylcarnitines, organic acids, and acylCoAs were analyzed using stable isotope dilution techniques. Amino acids and acylcarnitines were measured by flow injection tandem MS with specific internal standards (*36,37*); data were acquired using a Waters AcquityTM UPLC system equipped with a triple quadrupole detector and a data system controlled by MassLynx 4.1 OS (Waters, Milford, MA). Organic acids were quantified using Trace Ultra GC coupled to ISQ MS operating under Xcalibur 2.2 (Thermo Fisher Scientific) (*38*). AcylCoAs were extracted, purified and measured by flow injection analysis using positive electrospray ionization on a Xevo TQ-S triple quadrupole MS (Waters) (*39*); heptadecanoyl CoA was employed as an internal standard (*40*).

Quantitative proteomics
Plasma levels of leptin and insulin were quantified by using the MILLIPLEX MAP Human Bone Magnetic Bead Panel (MilliporeSigma). The human IGF-1 Quantikine ELISA (R&D Systems) was employed to measure IGF-1.

The SOMAscan 1.3K Proteomic Assay plasma/serum kit (SomaLogic, Boulder, CO, USA) was used to measure 1,305 proteins in plasma samples (50 μL aliquots). Following the manufacturer's protocol and utilizing SOMAmer reagents immobilized on streptavidin beads, proteins from plasma samples were tagged with NHS-biotin reagent, captured as a SOMAmer reagent/protein complex, cleaved, denatured, eluted and hybridized to a custom Agilent DNA microarray. Microarrays were scanned with an Agilent SureScan scanner at 5 μm resolution, and the Cy3 fluorescence readout was quantified. Raw signal values were processed using Somalogic's SOMAscan standardization procedures, including hybridization normalization, plate scaling, median scaling, and final SOMAmer calibration. The R package 'limma' (Bioconductor) was used to analyze differential protein abundances. In limma, signal data are subject to linear model fitting and empirical Bayesian statistics for group comparisons (*32*). Spearman correlation analyses were performed between measured SOMAscan analytes (proteins) and anthropometric scores, plasma metabolites, as well as the abundances of bacterial OTUs in fecal samples.

Proteins measured in the plasma of children with healthy growth phenotypes or with SAM (prior to treatment) were rank-ordered according to the fold-difference in their levels between these two groups. As noted in the main text, the top 50 most differentially abundant proteins in healthy compared to SAM were designated as healthy growth-discriminatory proteins, and the top 50 most differentially abundant in SAM compared to healthy were designated as SAM-discriminatory proteins. The average fold-change for these healthy growth- and SAM-discriminatory proteins was then calculated for each treatment arm in the MDCF trial (pre- versus post- MDCF/RUSF treatment) and normalized to the mean fold-change across all four arms (column normalization in **Fig. 6B**). Limma was used to calculate statistical significance.

Proteins with an absolute Pearson's r > 0.25 and FDR corrected p-value < 0.05 for HAZ were identified. The average fold-change in abundance for these 'HAZ-discriminatory proteins' was calculated for each treatment arm in the MDCF trial (pre- versus post-treatment) and normalized to the mean fold-change across all four arms (column normalization in **Fig. 6C**).

Proteins measured by the SOMAscan were mapped to all Gene Ontology (GO) 'Biological Processes' in the GO database (www.geneontology.org). SetRank, a gene set enrichment analysis (GSEA) algorithm (*33*), was employed to identify GO 'Biological Processes' that were significantly enriched for proteins that exhibited changes in abundance from pre- to post-treatment with MDCF/RUSF. Enrichment was calculated using the setRankAnalysis function in the SetRank R library (parameters: use.ranks = TRUE; setPCutoff = 0.01; and fdrCutoff = 0.05). The average fold-change for each protein in the statistically significant Biological Process category was calculated for each treatment arm and normalized to the mean fold-change across all four arms (**Fig. 7A-C**). We defined proteins within the GO Biological Process as 'healthy growth-discriminatory' if they were at least 30% more abundant in healthy individuals compared to those with SAM, and 'SAM-discriminatory' if they were at least 30% more abundant in children with SAM compared to those who were classified as healthy.

Characterizing human fecal microbial communities as a function of host nutritional status

V4-16S rRNA gene sequencing and data analysis - Frozen fecal samples were pulverized in liquid nitrogen. DNA was extracted from an aliquot of the pulverized material (~50 mg) by bead-beating with 500 μL of 0.1 mm diameter zirconia/silica beads in a solution consisting of 500 μL phenol:chloroform:isoamyl alcohol (25:24:1), 210 μL 20% SDS, and 500 μL buffer A (200 mM NaCl, 200 mM Trizma base, 20 mM EDTA). DNA was purified (Qiaquick columns, Qiagen), eluted in 70 μLTris-EDTA (TE) buffer, and quantified (Quant-iT dsDNA broad range kit; Invitrogen). Each DNA sample was adjusted to a concentration of 1 ng/μL and subjected to PCR using barcoded primers directed against variable region 4 of the bacterial 16S rRNA gene and the following cycling conditions: denaturation (94 ^{o}C for 2 minutes) followed by 26 cycles of 94 ^{o}C for 15 seconds, 50 ^{o}C for 30 seconds and 68 ^{o}C for 30 seconds, followed by incubation at 68 ^{o}C for 2 minutes (*2*). Amplicons were quantified, pooled and sequenced (Illumina MiSeq instrument, paired-end 250 nt reads). Paired-end reads (trimmed to 200 nt) were merged (FLASH, version 1.2.6), demultiplexed, clustered into 97% ID OTUs and aligned against the GreenGenes 2013 reference database using QIIME version 1.9.0 (*41*). Taxonomy was assigned to 97% ID OTUs with RDP 2.4, as described previously (*42*). The resulting OTU table was filtered to include only OTUs with ≥0.1% relative abundance in at least two samples.

As recent studies have produced newer methods for processing 16S rDNA data, a sensitivity analysis was performed comparing OTU assignments derived from QIIME with ASVs generated from DADA2. This analysis, described in (*31*), confirmed the concordance between the QIIME and DADA2 outputs.

Microbiota-for-age Z-scores – MAZ scores (*2*) were calculated using the sparse RF-derived Bangladeshi model of normal gut microbiota development, and the median and standard deviation of the predicted microbiota ages of the reference cohort of chronologically age-matched healthy Mirpur infants/children (binned by month).

Functional microbiome maturity - DNA was extracted from frozen fecal samples, quantified (Qubit), and each preparation was normalized to a concentration of 0.75 ng/μL. Libraries were generated from each DNA sample using the Nextera XT kit (Illumina) with the reaction volume scaled down 10-fold to 2.5 μL (*43*). Samples were pooled and sequenced (Illumina NextSeq instrument; paired-end 150 nt reads). A defined consortium of 16 human gut bacterial strains was included in each sequencing run as a reference control. Reads were quality filtered with Sickle (*44*) and Nextera adapter sequences were trimmed using cutadapt (*45*). Bowtie2 and the hG19

build of the *H. sapiens* genome were employed to identify and remove host sequences prior to further processing. Reads were subsequently assembled using IDBA-UD (*46*) and initially annotated with Prokka (*47*). Paired-end sequencing reads generated from each sample were mapped to contigs that had been assembled from that sample. Duplicate reads (optical- and PCR-generated) were identified and removed from mapped data using the Picard MarkDuplicates tool (v 2.9.3). Counts were aggregated for each gene (featureCounts; Subread v. 1.5.3 package) (*48*) and normalized (reads per kilobase per million, RPKM) in R (v. 3.4.1) (*49*).

Functional profiles for each fecal microbiome sample were generated by assigning microbiome-encoded proteins to a collection of 58 mcSEED subsystems/pathway modules that capture core metabolism of 75 nutrients/metabolites in four major categories (19 amino acids, 10 vitamins, 40 sugars, and 6 fermentation products) projected over 2,313 annotated reference bacterial genomes. The meta-proteomes from all samples were clustered at 90% identity [MMSeqs2 (*50*); --min-seq-id 0.9]. One representative protein sequence was randomly selected from each cluster. Clustering and representative protein sequence selection was performed in the same manner for all proteins in the 58 mcSEED subsystems/pathway modules. Representative proteins from the fecal meta-proteomes were queried against representative proteins from these mcSEED subsystems/pathway modules using DIAMOND (*51*) with the threshold for best hits set to ≥80% identity. As a result of this mapping, all members of a given cluster of microbiome-encoded proteins were assigned the best-hit annotation of the representative mcSEED protein.

A sparse RF-derived model was built using the aggregated mcSEED subsystem/pathway module abundances for all fecal samples collected from 10 healthy Bangladeshi children who had been sampled monthly from birth to 2 years of age. Applying this model to a separate test set of 20 healthy children sampled at 6, 12, 18, and 24 months of age gave a prediction of functional microbiome age. A smoothing spline function was fit between the predicted functional microbiome age and chronologic age of each individual at the time of fecal sample collection for these 20 healthy children. Limiting the model to the 30 subsystems/pathway modules with the highest feature importance scores did not significantly impact its accuracy. The resulting sparse RF-derived model explained 69.1% of the variance associated with age. The model was applied to a separate test set of 20 healthy Bangladeshi individuals sampled at 6, 12, 18, and 24 months of age. The correlation (Pearson's r) between chronological age and functional microbiome age in this test set was 0.66 ($P = 4.4 \times 10^{-6}$), with a mean absolute error (MAE) of 3.9 months and root mean square error (RMSE) of 5.1 months (29.2% of the mean).

The sparse RF-derived model was then applied to the mcSEED subsystem/pathway module abundance profiles of fecal samples obtained from children with SAM prior to, during and after treatment. Relative functional maturity for each sample was calculated by subtracting the functional microbiome age of that sample from the spline fit functional microbiome age of samples obtained from healthy children of similar chronologic age.

Quantifying enteropathogen burden by multiplex qPCR – Nucleic acids were isolated from fecal samples and adjusted to 2ng/µL. Levels of 18 bacterial and viral pathogens and parasites were determined by using a microfluidic-based digital PCR system with 96.96 Dynamic Arrays (Fluidigm Corp. San Francisco, CA). TaqMan primers and probes (*52*) were used to construct the 24 different assays employed for this analysis. cDNAs were prepared from 50 ng of total RNA using Life Technologies High Capacity cDNA Reverse Transcription Kit (Applied Biosystems, Foster City, CA). The resulting products were subjected to Specific Target Amplification (STA) using TaqMan PreAmp Mastermix (Applied Biosystems), 50 nM of each primer, and the following cycling conditions; 10 minutes at 95 °C followed by 14 cycles of 95 °C for 15 seconds and then 60 °C for 1 minute. At the conclusion of this step, the reaction mixture was diluted 1:4 in low EDTA DNA suspension buffer (10 mM Tris, 0.1 mM EDTA, pH 8.0) combined with TaqMan Universal PCR Master Mix (Applied Biosystems) and 20X Gene Expression Sample

Loading Reagent (Fluidigm). Assay mixtures containing 9 μM of each primer and 2 μM of the probe in Dynamic Array Assay Loading Reagent (Fluidigm) (*52*) were loaded into appropriate inlets on the primed 96.96 Dynamic Array chip before it was placed on the NanoFlex-4 Integrated Fluidic Circuit Controller for distribution of the sample and assay mixture. The loaded Dynamic Array was then inserted into the BioMark™ Reverse-Transcription-PCR System. The qPCR program consisted of the following steps: 50 ^{o}C for 2 minutes, 95 ^{o}C for 2 minutes and 40 cycles of 95 ^{o}C for 15 seconds and 60 ^{o}C for 1 minute. Enteropathogen abundance was calculated by comparing cycle threshold to standards of known concentration, yielding absolute measurements of pg genomic DNA (bacterial enteropathogens and parasites), copy number (RNA viruses) and mass of viral DNA per lysate mass (Adenovirus).

GNOTOBIOTIC MOUSE STUDIES

All mouse experiments were performed using protocols approved by Washington University Animal Studies Committee. Mice were housed in plastic flexible film gnotobiotic isolators (Class Biologically Clean Ltd., Madison, WI) at 23°C under a strict 12-hour light cycle (lights on a 0600h). Male germ-free C57BL/6 mice were initially weaned onto an autoclaved, low-fat, high-plant polysaccharide chow (2018S, Envigo) that was administered *ad libitum*. Animals were maintained on this diet until 3 days prior to the beginning of experiments involving tests of the effects of complementary food ingredients. Using a disposable sterile gavage needle, defined consortia of sequenced age-discriminatory bacterial strains cultured from Bangladeshi children, or intact uncultured microbiota from donors with post-SAM MAM were introduced into recipient mice at 5 weeks of age. All animals were euthanized by cervical dislocation without prior fasting.

Screen of CFCs described in fig. S2 and the monotonous feeding experiments involving the initial MDCF prototype and MS/KH described in Fig. 3.

Design and preparation of diets **-** Bangladeshi diets were constructed using extensive knowledge of Bangladeshi complementary feeding practices, including quantitative 24 hour dietary recall surveys conducted at the Mirpur site as part of the MAL-ED study [see (*53*) for a description of methods]. All diets were prepared by Dyets, Inc. (Bethlehem, PA). The compositions and quantities of each ingredient used to prepare each diet are provided in **table S8B,D**.

To prepare the Mirpur-18 diet (**table S8D**), rice (parboiled, long grain) and red lentils (masoor dal) were each cooked separately with an equal weight of water at 100°C in a steam-jacketed kettle until 'par-cooked' (grains cooked, but still firm) and then set aside. Fresh market white potatoes, spinach and yellow onions were washed, chopped in a vertical cutter mixer and cooked in the kettle without added water at 70°C until soft. Sweet pumpkin (Calabaza variety) was cut and boiled in the steam-jacketed kettle until soft, and then strained. At this point, all of the cooked ingredients were combined, whole bovine milk powder (Franklin Farms East, Bethlehem, PA), soybean oil, salt, turmeric and garlic were added and the resulting diet was mixed extensively.

To prepare the CFC diets, rice, red lentils, potato, spinach and sweet pumpkin were cooked as described above for Mirpur-18. Canned garbanzo chickpeas and ground peanuts were roasted separately in a small amount of water for 8-10 minutes and blended into a paste prior to use. Tilapia (frozen fillets) was placed in the kettle with a small amount of water to steam until cooked thoroughly (~20 minutes). Eggs were scrambled. Banana, whole milk powder and whole-wheat flour (*atta*) were not cooked. Individual CFC diets were prepared by combining their various components in the quantities listed in **table S8B** and mixed thoroughly using a planetary mixer prior to pelleting.

To generate Khichuri-Halwa, Khichuri (**table S8D**) was prepared by first cooking rice and red lentils in a steam kettle (Groen) at 100 ^{o}C with an equal weight of water until the grains were

cooked but still firm. White potato, spinach and yellow onions were washed and chopped in a vertical cutter mixer and cooked with spices in the steam kettle without added water at 70 °C until soft. Sweet pumpkin was cut and boiled in the kettle until soft, and then strained. Cooked ingredients were then combined on a weight basis in the proportions shown in **table S8D**. To prepare Halwa (**table S8D**), jaggery was added to the steam kettle with water and heated (70 °C) until it was fully dissolved, after which time cooked lentils were added. This hot mix was added to the bowl of a planetary mixer in which *atta* flour (pre-roasted for 5 minutes with a small amount of water) and soybean oil had been pre-mixed. The Halwa was blended extensively into a uniform thick paste. Milk suji was prepared by combining whole bovine milk powder, rice powder, sugar and soya oil along with minerals in the amounts listed in **table S8D**. The individual components (milk suji, Khichuri, and Halwa) were then combined at a ratio of 28:36:36 (to simulate the relative contributions of these components to dietary intake during the nutritional rehabilitation phase of treatment of children with SAM at Dhaka Hospital) and homogenized.

Once all diets had been prepared, they were spread on trays, dried overnight at 30 °C, and pelleted by extrusion (½" diameter; California Pellet Mill, CL5). Dried pellets were weighed into ~250g portions, placed in a paper bag with an inner wax lining, which in turn was placed in a plastic bag. The material was vacuumed sealed and sterilized by gamma irradiation (30-50 kGy; Sterigenics, Rockaway, NJ). Sterility was assessed by culturing irradiated pellets in Brain Heart Infusion (BHI) broth, Nutrient broth, and Sabouraud-dextran broth (all from Difco) for one week at 37 °C under aerobic conditions, and in Tryptic Soy broth (Difco) under anaerobic conditions (atmosphere of 75% N_2, 20% CO_2 and 5% H_2; vinyl anaerobic chambers from Coy Laboratory Products, Grass Lake, MI). Additionally, cultures of all diets were plated on BHI agar supplemented with 10% horse blood (Difco). The irradiated diet pellets were subjected to nutritional analysis (Nestlé Purina Analytical Laboratories; St. Louis, MO) (see **table S8E** for results). All diets were stored at -20 °C prior to use.

Isolation and culturing of age-discriminatory and SAM-associated bacterial strains - Bacterial strains were cultured from fecal samples collected from a 24-month-old child enrolled in the SAM study described above, and from three donors aged 6-24 months that exhibited healthy growth as defined by serial anthropometry. These latter three children were members of two previously completed Bangladeshi birth cohort studies; (i) 'Field Studies of Amebiasis in Bangladesh' (ClinicalTrials.gov identifier: NCT02734264) and (ii) 'Interactions of Enteric Infections and Malnutrition and the Consequences for Child Health and Development' (abbreviated 'Malnutrition and Enteric Disease Study' (MAL-ED) ClinicalTrials.gov identifier: NCT02441426)] (*53*, *54*). Protocols for these two studies were approved by the Ethical Review Committee at icddr,b. Informed consent was obtained from the mother/guardian of each child. Collection and use of biospecimens from each of the human studies was approved by the Washington University Human Research Protection Office (HRPO).

Collections of cultured anaerobic bacterial strains were generated from frozen fecal samples according to previously published methods (*55*, *56*). All procedures were performed under anaerobic conditions in a Coy chamber. Fecal samples (~0.1 g aliquots) were weighed, brought into the Coy chamber, diluted 1:10 (wt/vol) with reduced PBS (PBS/ 0.05% L-cysteine-HCl) in 50 mL conical plastic tubes containing 5 mL of 2 mm-diameter glass beads (VWR, catalog number 26396-506). Tubes were gently vortexed and the resulting slurry was passed through a 100 μm-pore diameter nylon cell strainer (BD Falcon). The clarified fecal sample was then combined with an equal volume of a solution of PBS/0.05% L-cysteine-HCl/30% glycerol and aliquoted into 1.8 mL glass vials (E-Z vials, Wheaton). Tubes were crimped with covers containing a PTFE/grey butyl liner (Wheaton) and stored at -80 °C.

Frozen stocks were brought into the Coy chamber, thawed and serially diluted over a 1000-fold range with PBS/0.05% L-cysteine-HCl. 100 μL of each dilution were spread on agar plates

containing MegaMedium and 0.05% L-cysteine-HCl (*55*, *57*). Plates were incubated at 37 °C under anaerobic conditions for 48 h. Single colonies were handpicked into 96-deep-well plates (Thermo Fisher Scientific) containing 600 μL of MegaMedium broth. Deep-well plates were subsequently incubated at 37 °C under anaerobic conditions for 48 h, at which time a 50 μL aliquot from each deep well was robotically transferred into a well of a 96-well shallow plate containing an equal volume of PBS/0.05% L-cysteine-HCl/30% glycerol (n=2 replicate stock plates; stored at -80 °C). The deep well plate with the remaining 500 μL in each well was removed from the Coy chamber and subjected to centrifugation (3,220 x g for 20 min at 4 °C). Using a liquid handling robot, the resulting supernatant was removed and DNA was extracted from cell pellets with phenol:chloroform. V4-16S rDNA amplicons were generated by PCR and sequenced (Illumina MiSeq; paired-end 250 nt reads).

Isolates whose V4-16S rDNA sequences shared ≥97% sequence identity with age-discriminatory 97%ID OTUs and/or were enriched in the microbiota of children with SAM were selected for an additional round of colony purification. Full-length 16S rRNA gene amplicons were generated from these isolates using primers 8F and 1391R (*58*). Isolates sharing ≥99% nucleotide sequence identity in their full length 16S rRNA genes, and ≥96% nucleotide sequence identity throughout their genomes [NUCmer; (*59*); (see next paragraph)], were defined as unique strains. Taxonomy was assigned based on the full-length 16S rDNA sequences (RDP version 2.4 classifier; **table S6A**). Purified, sequenced strains were each grown to mid-log phase in MegaMedium; stocks were then prepared (15% glycerol/MegaMedium) in crimped vials and stored at -80 °C.

Bacterial genome sequencing, assembly, annotation, in silico reconstructions and phenotype predictions - Barcoded, paired-end genomic libraries were prepared for each bacterial isolate DNA sample, and the libraries were sequenced in multiplex (Illumina MiSeq instrument; paired-end 150 nt or 250 nt reads). Reads were de-multiplexed and assembled using SPAdes (*60*) (**table S6A,C**). Contigs with greater than 10X coverage were initially annotated using Prokka (*47*). Genes in each genome were also annotated at various levels by mapping protein sequences to the Prokaryotic Peptide Sequence database of the Kyoto Encyclopedia of Genes and Genomes (KEGG, 4 March 2017 release; $1x10^{-5}$ E-value threshold for BLASTP searches; *61*).

Subsystems-based, context-driven functional assignments of genes, curation and metabolic reconstructions were performed in the web-based mcSEED (microbial communities SEED) environment, a private clone of the publicly available SEED platform (*62*, *63*). The mcSEED platform currently includes: (i) ~6,000 bacterial genomes carefully selected for phylogenetic diversity, including a subset of 2,300 reference mammalian gut microbial genomes representing 690 species (*64*) and (ii) a collection of curated metabolic subsystems. These subsystems include a subset of 58 biosynthetic, salvage and utilization pathway modules for amino acids, B-vitamins and related cofactors, carbohydrates, central carbon metabolism and fermentation, projected over ~200 genomes representing the cultured strains described in this report and their nearest phylogenetic neighbors. *In silico* reconstructions of selected metabolic pathways were based on functional gene annotation and prediction using homology-based methods supplemented by three genome context techniques: (i) clustering of functionally-related genes on the chromosome (operons) compared to closely-related annotated genomes, (ii) predicted co-regulation of genes by a common regulator (regulons) and (iii) co-occurrence of genes in a set of related genomes. Context-based techniques are particularly helpful in disambiguating paralogs with related but distinct functions (characteristic for sugar utilization pathways, most notably transporters and transcriptional regulators), filling in gaps (“missing genes”) in known pathway variants, including functional assignments (predictions) of previously uncharacterized protein families (e.g., non-orthologous gene replacements), and inferring alternative biochemical routes. Initial training sets of transcription factor binding sites (TFBS) and co-regulated genes were taken from the

RegPrecise database of bacterial regulons (*65*). RNA regulatory elements (riboswitches) were determined using RibEx (*66*). Note that mcSEED pathways may be more granular than a subsystem (e.g. uptake of a nutrient versus its metabolism). mcSEED subsystems/pathway modules are presented as lists of assigned genes and their annotations in **table S11A**. Predictions are presented here in the form of a Binary Phenotype Matrix with **table S6D-G** showing the supporting evidence (presence/absence of genes in a pathway). Information from the Carbohydrate Active Enzyme (CAZy) database (http://www.cazy.org) was integrated into the annotations to expand subsystem/pathway module coverage for utilization of complex carbohydrates.

COmmunity PROfiling by Sequencing (COPRO-Seq) – The effects of diet on the configuration of the defined consortium of cultured strains was defined by COPRO-Seq (*67*). Briefly, DNA was isolated by subjecting fecal pellets or cecal contents collected from gnotobiotic mice, to bead-beating for 3 minutes in a mixture containing 500 µL Buffer A (200 mM NaCl, 200 mM Tris, 20 mM EDTA), 210 µL 20% SDS, 500 µL phenol:chloroform:isoamyl alcohol (25:24:1, pH 7.9), and 250 µL of 0.1 mm diameter zirconium beads (BioSpec Products). Bead-beating was performed in 2 mL screw cap tubes (Axygen) using a Mini-Beadbeater-8 (Biospec). The aqueous phase was collected after centrifugation at 4 °C for 5 min at 8,000 x g. Nucleic acids were purified with QIAquick columns (Qiagen) and eluted with nuclease-free water (Ambion).

COPRO-Seq libraries were prepared by first sonicating 100 µL of a 5 ng/µL solution of DNA from each sample [Bioruptor Pico (Diagenode, New Jersey, USA); 10 cycles of 30 seconds on / 30 seconds off at 4 °C]. Fragmented DNA was concentrated in MinElute 96 UF PCR Purification plates (Qiagen). Fragments were blunt-ended, an "A"-tail was added, and the reaction products were ligated to Illumina paired-end sequencing adapters containing sample-specific, 8 bp in-line barcodes. Size selection was performed (1% agarose gels); 250-350 bp fragments were excised from the gel and the DNA was purified by MinElute Gel Extraction (Qiagen). Adapter-linked fragments were enriched by a 20-cycle PCR using Illumina PCR Primers PE 1.0 and 2.0 followed by treatment with the MinElute PCR Purification kit. Barcoded libraries were quantified (Qubit dsDNA HS kit), pooled and subjected to multiplex sequencing [Illumina NextSeq instrument, unidirectional 75 nt reads; $5.4\times10^{6} \pm 4.7\times10^{6}$ reads/sample (mean ± SD); n = 162 samples]. Reads were demultiplexed and mapped to the reference genomes of community members, plus six "distractor" genomes (*Lactobacillus ruminis* ATCC 27782, *Megasphaera elsdenii* DSM 20460, *Olsenella uli* DSM 7084, *Pasteurella multocida* subsp. *multocida* str. 3480, *Prevotella dentalis* DSM 3688, and *Staphylococcus saprophyticus* subsp. *saprophyticus* ATCC 15305). The proportion of reads mapping to "distractor" genomes in each sample was used to set a conservative cutoff threshold (mean + 2 SD), indicating the presence/absence of an organism in the community on a per-sample basis. Normalized counts for each bacterial strain in each sample were used to produce a relative abundance table.

Microbial RNA-Seq – The effects of MS/KH and the initial MDCF prototype containing three selected complementary foods (chickpea, banana and tilapia) were tested in monotonous feeding experiments involving mice that had been colonized at 5 weeks of age with the defined consortium of 14 bacterial strains (n = 3 cages of dually-housed animals/treatment group). Methods used for isolation of RNA from cecal contents, processing of transcripts for microbial RNA-Seq, sequencing [Illumina NextSeq instrument; unidirectional 75 nt reads; $1.2\times10^{7} \pm 2.7\times10^{6}$ reads/sample (mean ± SD); n = 24 samples], and analysis of the resulting datasets are described in a previous publication (*68*). Briefly, sequence data were mapped to the genomes of community members. Raw counts were subsetted, normalized and analyzed by two complementary strategies. To analyze data at the community level ('top-down' view of the meta-transcriptome), or to obtain a strain-level view of transcriptional responses ('bottom-up' analysis), raw read count data for each sample were filtered at a low abundance threshold of three

reads. An additional filter for consistent representation in biological replicates was then applied (present in ≥66% of samples in a given treatment group, or present in all samples in one group and in none of the other). The resulting dataset was then imported into R and differential expression analysis was performed using DESeq2 (*69*).

KEGG-annotated gene lists for each organism (or the community in aggregate) were processed into gene sets in R (v3.4.1; (*49*)), and subsequently used for complementary pathway enrichment analyses with the R packages clusterProfiler [v3.4.4; (*70*)) and GAGE (v2.26.1; (*71*)]. For hypergeometric enrichment tests, lists of differentially expressed genes were supplied to the clusterProfiler 'enricher' function along with corresponding gene set information. DESeq2-normalized counts were supplied along with corresponding gene set information to GAGE, with settings to order genes by the non-parametric Wilcoxon Rank Sum statistic ("rank.test = T, saaTest = gs.tTest") and to allow genes displaying both increased and decreased expression in each tested level of the KEGG hierarchy to be considered ("same.dir = F"). P-values were adjusted to control false discovery rate (Benjamini-Hochberg method).

Targeted Mass Spectrometry – Aliquots of cecal contents taken from the same animals used to compare microbial gene expression in mice monotonously fed MS/KH and the initial MDCF prototype, plus comparably fed germ-free controls were subjected to targeted mass spectrometry (n = 4 treatment groups; 3 cages of dually-housed mice/group). Quantification of targeted metabolites was performed by using external standards. For cecal amino acids, monosaccharides and disaccharides, flash frozen cecal contents were homogenized in 20 vol/wt of HPLC grade water. Homogenates were centrifuged (4,000 x g for 10 minutes at 4 °C). A 200 µL aliquot of each supernatant was combined with ice-cold methanol (400 µL). The mixture was vortexed, centrifuged (8,000 x g at 4 °C), and a 500 µL aliquot of the resulting supernatant was evaporated to dryness. Dried samples were derivatized by adding methoxylamine (80 µL of a 15 mg/mL stock solution prepared in pyridine) to methoximate reactive carbonyls (incubation for 16 h at 37 °C), followed by replacement of exchangeable protons with trimethylsilyl groups using *N*-methyl-*N*-(trimethylsilyl) trifluoroacetamide (MSTFA) together with a 1% v/v catalytic admixture of trimethylchlorosilane (1 h incubation at 70 °C). Heptane (160 µL) was added and a 1 µL aliquot of each derivatized sample was injected into an Agilent 7890B/5977B GC/MS system.

Tryptophan and its metabolites were quantified using an ion pair-based reverse phase (IP-RP) chromatographic method. Chromatographic separation was achieved using an Agilent ZORBAX Extend C18 RRHD 2.1 x150 mm, 1.8 µm column with the ion-pairing agent tributylamine added to the mobile phases. A Model 1290 Infinity II UHPLC quaternary pump was coupled to an Agilent 6470 Triple Quadrupole LC/MS system equipped with a Jet Stream electrospray ionization source. dMRM (dynamic multiple reaction monitoring) parameters including precursor, product ions and retention times were determined using chemical standards. MassHunter Optimizer Software was used to determine optimal collision energies and fragmentor voltages for each metabolite. The protocol for GC-MS of short chain fatty acids is described in a previous publication (*56*).

To measure amino acids, acylcarnitines, organic acids and acylCoAs in liver, gastrocnemius muscle and serum, samples were weighed while frozen and homogenized in 50% acetonitrile containing 0.3% formic acid (50 mg wet weight tissue/mL solution) using a high-speed homogenizer (IKA, catalog number EW-04739-21) set at maximum speed for 30 seconds. Amino acid and acylcarnitine measurements were made by flow injection tandem mass spectrometry, and with specific internal standards (*36, 37*). Data were acquired using a Waters AcquityTM UPLC system equipped with a triple quadrupole detector and a data system controlled by MassLynx 4.1 OS (Waters, Milford, MA). Organic acids were quantified using Trace Ultra GC coupled to ISQ MS operating under Xcalibur 2.2 (Thermo Fisher Scientific, Austin, TX) (*38*).

AcylCoAs were extracted, purified, and analyzed by flow injection using positive electrospray ionization on a Xevo TQ-S triple quadrupole mass spectrometer (Waters, Milford, MA) (*39*). Heptadecanoyl CoA was employed as an internal standard (*40*).

***Western blot analysis of IGF-1 pathway components in liver* -** Liver proteins were isolated, quantified, separated by electrophoresis (4-20% gradient SDS-polyacrylamide gels) and subjected to Western blotting (*72*). The same amount of total protein was analyzed from each liver sample. The following primary antibodies, all generated in rabbits except for anti-Akt(pan), were purchased from Cell Signaling Technology: anti-phospho-AMPKα(Thr172) [catalog number 2531], anti-Akt(pan) [catalog number 2920], anti-phospho-Akt(Ser473) [catalog number 4060], anti-Jak2 [catalog number 3230], anti-phospho-Jak2(Tyr1007/1008) [catalog number 3776], anti-mTOR [catalog number 2983], anti-phospho-mTOR(Ser2448) [catalog number 5536], anti-Stat 5 [catalog number 9363], and anti-phospho-Stat 5(Tyr694) [catalog number 9351]. Primary antibodies were incubated with Western blots overnight at 4 °C in a solution of Tris-buffered saline containing 0.1% Tween-20 (TBST) plus 1% (vol/vol) non-fat milk, followed by addition of secondary antibodies against rabbit or mouse immunoglobulins and a 1 h incubation at room temperature in TBST/1% nonfat milk. Protein bands were detected by chemiluminescence (Western Lightning® Plus-ECL, PerkinElmer) using the LI COR Odyssey® FC imaging system, and quantified by densitometry. The amount of phosphorylated protein was normalized to the total amount of the protein or to GAPDH.

***Micro-computed tomography (μCT) of bone* -** Femurs were harvested from mice at the time of euthanasia and soft tissue was removed. Bones were fixed for 24 hours in 70% ethanol and stored at 4 °C prior to scanning. Micro-computed tomography was performed using a μCT 40 desktop cone-beam instrument (ScanCO Medical, Brüttisellen, Switzerland). For cortical bone analysis, 100-200 slices were taken for each sample in the transverse plane with a 6 μm voxel size (high resolution); slices began at the midpoint of the femur and extended distally. For trabecular scans, 600 to 700 slices were taken beginning at the proximal end of the growth plate and moving along the femur until no further trabeculae were observed. Boundaries of, and thresholds for bone were drawn manually using μCT 40 software. Volumetric parameters (bone volume/tissue volume, bone mineral density and cortical thickness) were calculated using custom scripts.

***IGF-1 ELISA* -** IGF-1 levels were measured in mouse serum samples using the R&D Systems DuoSet ELISA kit according to the manufacturer's instructions. Samples were diluted 1:100 in Reagent Diluent and assayed in duplicate. Optical density was quantified (BioTek Synergy 2 plate reader) and the resulting data were analyzed with GraphPad Prism software (version 7.00 for Mac).

Screening 16 plant-derived complementary food ingredients in gnotobiotic mice (fig. S4)
We generated 48 diets by supplementing the Mirpur-18 base diet with 16 different plant-based ingredients at three different concentrations (**table S13A**). We also prepared diets in which three of the 16 complementary food ingredients (chickpea, peanut and soybean) were incorporated as 'flours' to compare the effects of raw versus processed forms (the levels of each of these flours were matched to the protein content of the corresponding unprocessed forms). The ingredients for each diet were cooked, homogenized, extruded as pellets, dried, sterilized, and sterility was confirmed as described above.

Five-week-old germ-free male C57BL/6J mice were gavaged with a consortium of 20 cultured, sequenced bacterial strains consisting of: (i) seven weaning-phase age-discriminatory strains from healthy Mirpur donors, (ii) five strains from a 24-month-old SAM donor, four of which are prominent in the first 8-11 months of postnatal life, (iii) two milk-adapted strains from a 6-month-old healthy Mirpur donor, (iv) three strains prevalent in the Bangladeshi children with

post-SAM MAM (*Clostridium amygdalinum, Eggerthella lenta, Lactobacillus gasseri*), and (v) three weaning-phase 'growth-discriminatory' strains recovered from the fecal microbiota of Malawian children (*Clostridium symbiosum*, *Ruminococcus gnavus, Clostridium nexile*) (*4*). *In silico* metabolic reconstructions of the predicted requirements of these cultured strains for amino acids and B-vitamins, plus their capacity to utilize mono- and disaccharides are presented in **table S6D-G**.

Colonized mice (n = 24; singly-housed in cages containing paper houses for environmental enrichment) were subjected to an 8-week diet oscillation. To minimize the effects of hysteresis, each mouse was fed a different diet every week, and no mouse was given the two forms of an ingredient (i.e., raw and flour forms) in consecutive weeks. Replication was achieved by presenting each diet four times to different mice.

The relative abundances of bacterial community members were determined by COPRO-Seq analysis of DNA isolated from fecal samples collected at the end of each week of the diet oscillation protocol [Illumina Nextera DNA Library Prep Kit; Illumina NextSeq instrument; 75 nt unidirectional reads, $2.2x10^6 \pm 3.3x10^5$ reads/sample (mean ± SD), n=192 samples; **table S13C**]. Eighteen strains were detected in all animals at all time points surveyed; one of the three post-SAM MAM strains and one of the SAM-derived strains failed to colonize recipient mice (*L. gasseri* and *S. constellatus*, respectively; abundance <0.001%).

Effects of complementary food ingredients in mice harboring a post-SAM MAM microbiota
Fifteen different microbial communities from 12 different participants in the SAM trial, collected during and/or after treatment, were introduced into separate groups of 5-week-old germ-free mice (n=4/donor microbiota; dually-housed). Half of the animals in each recipient group were given Mirpur-18 without supplementation, while the other half were given a complementary food formulation composed of peanut, chickpea, banana, tilapia, and milk powder (PCBT diet). Ten days later all animals in the two groups were switched to Mirpur-18 supplemented with peanut, chickpea, banana, and tilapia [Mirpur(PCBT)] and maintained on that diet for 10 days (see **table S14A** for the composition of these diets). The goal was to identify those fecal microbiota samples that contained the greatest number of transmissible weaning-phase age-discriminatory bacterial taxa, and that when transplanted into mice, exhibited increases in the relative abundances of these targeted organisms with supplementation of the Mirpur-18 diet. Based on the results from this screen (**table S14B**), we selected a sample obtained from a donor (PS.064) at the S7 time point with post-SAM MAM for a follow-up gnotobiotic mouse study. A 350 mg aliquot of this frozen fecal sample was brought into an anaerobic Coy chamber, vortexed in PBS with glass beads, filtered, and the clarified sample was aliquoted into glass vials prior to storage at -80 °C as described above.

In a follow-on study, mice received an oral gavage of 100 μL sterile 1M sodium bicarbonate followed by 100 μL of the clarified human fecal sample. Animals were given unsupplemented Mirpur-18 diet, or Mirpur-18 supplemented with peanut flour [Mirpur(P)], or Mirpur-18 supplemented with peanut flour, chickpea flour, soy flour and banana [Mirpur(PCSB)] *ad libitum*. The supplemented diets were matched for total protein content (**table S15A**). Age- and sex-matched germ-free C57BL/6J mice fed the same diets served as controls (n = 5 mice/treatment group; group-housed).

Characterizing the transplanted fecal microbiome from a donor with post-SAM MAM in recipient gnotobiotic mice as a function of diet treatment - DNA was extracted from the cecal contents of each mouse in each diet treatment group, quantified (Qubit) and normalized to a concentration of 0.5 ng/μL. Genomic libraries were prepared from each cecal DNA sample (n = 5/treatment group) using the Illumina Nextera XT kit in a reaction volume of 2.5 μL. Paired-end 150 nucleotide datasets were generated for each library by multiplex sequencing with an Illumina

NextSeq instrument. Reads were processed, assembled, annotated, and the representation of mcSEED subsystems/pathway modules was determined as described above.

Methods used for isolation of RNA from cecal contents, processing for microbial RNA-Seq [Illumina NextSeq instrument; unidirectional 75 nt reads; $6.79 \pm 3.35 \times 10^6$ reads/sample (mean ± SD); 14 samples], and analysis of the resulting datasets are described in Hibberd *et al.* (*68*). Data were analyzed at the community level and at the strain-level for *F. prausnitzii* JG_BgPS064. Differential expression was defined using DESeq2 (*69*). A total of 6,390 genes were found to be differentially expressed in at least one pairwise comparison of the three diets. These genes were subjected to enrichment analysis over the 58 mcSEED subsystems/pathway modules. Of the differentially expressed genes with best-scoring BLAST hits (filtered to include only those spanning at least 90% of the query amino acid sequence) within 2313 annotated mcSEED genomes representing the human gut microbiome, 1099 genes (17.7%) were attributed to the analyzed subsystems/pathway modules. mcSEED-annotated gene lists were used to generate gene sets in R and subsequently employed for pathway enrichment analysis with the GAGE R package (v2.26.1) (*71*). P-values were adjusted to control false discovery rate (Benjamini-Hochberg method).

Histochemical and immunohistochemical analysis – Immediately after euthanasia, the entire length of the small intestine was removed from each animal and evenly divided into proximal (SI-1), middle (SI-2), and distal (SI-3) segments. Each of these small intestinal segments was further subdivided into thirds yielding three subsegments. The most proximal third sub-segment was placed in Carnoy's fixative. The middle third sub-segment was perfused with and embedded in Optimal Cutting Temperature (OCT) compound (Tissue-Tek) and then snap frozen in a methanol-dry ice bath. The distal third sub-segment was snap frozen in liquid nitrogen. Frozen samples were stored at -80°C.

The proximal third of each subsegment was transferred from Carnoy's fixative into 70% ethanol and embedded in paraffin. Five micron-thick sections were prepared and stained with hematoxylin and eosin. OCT embedded blocks of the middle third sub-segments obtained from SI-1, SI-2, and SI-3 were sectioned at 5 μm thickness onto charged, uncoated glass slides (Superfrost Plus) in a cryostat at -20 °C. Following cryosectioning, slides were stained for 15 minutes at room temperature with Safranin O and Alcian Blue pH 2.5 (Abcam) to identify nuclei and mucosa-associated bacteria, acidic mucopolysaccharides, and glycoproteins. Slides were then dehydrated with graded alcohols (Richard-Allan Scientific), rinsed with xylenes, and stored at room temperature in an airtight container with desiccant for 12 to 16 hours. Fifty crypts in a hematoxylin and eosin-stained SI-2 subsegment were analyzed per mouse per treatment group (n=5 animals/group). Villus-to-crypt ratios were calculated by measuring villus and crypt lengths in the 10 best-oriented villus-crypt units per hematoxylin- and eosin-stained SI-2 section per mouse per treatment group. Goblet cell and Paneth cell numbers were scored (n=10 crypts per hematoxylin- and eosin-stained SI-2 section/animal). Submucosal lymphoid aggregates were counted and measured in sections prepared from SI-1 and SI-2. Sections were also stained with CD3 (Abcam, catalog number ab5690), CD20 (Thermo Fisher, catalog number PA5-16701), and IgA (Abcam, catalog number ab97235). A biotin-conjugated, goat anti-rabbit antibody (Jackson ImmunoResearch, catalog number 111-065-003, diluted 1:800 in PBS/0.1% Tween 20) was applied, followed by incubation with horseradish peroxidase-conjugated streptavidin (Jackson ImmunoResearch, catalog number 016-030-084, 1:1200) and detection with betazoid 3, 3' Diaminobenzidine (Biocare Medical). Nuclei were visualized with a hematoxylin counterstain (Leica).

Laser capture microdissection (LCM) - Infrared laser capture microdissection of the small intestinal epithelium in a 20X field of view of a well-oriented section, prepared from the OCT-embedded subsegment, was performed by using the Arcturus Pix Cell IIe system with Arcturus

CapSure Macro Caps (Applied Biosystems). RNA extraction was performed immediately after LCM using the Arcturus PicoPure RNA extraction kit (Applied Biosystems) and treatment with Baseline-ZERO DNase (Epicentre). RNA quality was checked with an Agilent Bioanalyzer 2100 using RNA 6000 Pico Chips (Agilent).

All Alcian Blue-stained, mucosal-associated material present in two 20X fields of view of sections prepared from OCT-embedded SI-1, S1-2, and SI-3 sub-segments were subjected to LCM and DNA was isolated [Arcturus PicoPure DNA extraction kit (Applied Biosystems) with a 16-h incubation in proteinase K (1 μg/μL, ThermoFischer; 65 °C)]. To quantify community configuration along the length of the small intestine as a function of diet**,** V4-16S rDNA amplicons were generated from these mucosal DNA samples and sequenced. The resulting OTU table was filtered to include only OTUs with ≥0.1% relative abundance in at least two samples, and then rarefied to 2,000 reads/sample.

RNA isolated from LCM mucosa was used to characterize the effects of diet on jejunal gene expression. cDNA was synthesized from 10 ng of total RNA using the 'SMARTer Ultra Low Input RNA for Illumina Sequencing-HV' kit (Clontech). Successful cDNA synthesis was verified using a Bioanalyzer 2100 and High Sensitivity DNA Chips (Agilent). The products were sheared to 200-500 bp with a Covaris AFA system. Libraries were constructed by following the Clontech 'adapted Nextera (Illumina) DNA sample preparation protocol for use with 'SMARTer ultralow DNA kit for Illumina sequencing'. A total of 21 jejunal mucosal samples were sequenced [Illumina NextSeq instrument; NextSeq Series High-Output Kit; 75 nucleotide paired-end reads; $19.8 \pm 5.1 \times 10^6$ reads/sample (mean ± SD); for PS.064.S7-colonized mice, n=4 samples from animals consuming the unsupplemented Mirpur-18 diet, 4 samples from those fed Mirpur(P), and 5 samples from mice treated with Mirpur(PCSB); n = 3, 2 and 3 from the corresponding groups of germ-free animals].

Reads were aligned to the *Mus musculus* GRCm38.p6 genome assembly with STAR version 2.5.3a. Gene count data were derived from the number of uniquely aligned reads by featureCounts from Subread version 1.4.6-p5 (*48*). Sequencing performance was evaluated using RSeQC version 2.6.2. Gene counts were imported into the R/Bioconductor package edgeR and normalized (weighted trimmed mean of M-values), then filtered to remove genes with less than one count per million from further analyses. The R/Bioconductor package limma was used to perform differential expression analysis, and results were filtered by FDR adjusted p-values ≤0.05. Gene set enrichment analysis was performed and results visualized in R (packages GAGE and Pathview).

Isolation, sequencing, and genome annotation of *F. prausnitzii* strain JG_BgPS064

A cecal sample that had been obtained from a gnotobiotic mouse colonized with the PS.064.S7 donor community and stored at -80 °C was brought into an anaerobic Coy chamber, diluted to a concentration of 0.35 g/5 mL in Wilkins Chalgren anaerobic broth (Oxoid, Ltd.), and the slurry was vortexed 3 times at 30 second intervals. Serial dilutions to 10^{-8} were made in Wilkins Chalgren broth, and aliquots (100 μL each) were plated on Wilkins Chalgren agar plates or YHBHI+A plates [YHBHI (*73*) plus 1 mL/L acetic acid], with or without antibiotics to which *F. prausnitzii* is frequently resistant [sulfamethoxazole (25 mg/L) and trimethoprim (1.25 mg/L)]. Plates were incubated at 37 °C for 5 days; 32 single colonies per media type (128 colonies total) were picked and plated in duplicate. Selection for Extremely Oxygen Sensitive (EOS) bacteria was performed (*74*). Five single colonies were picked from plates that had remained in the anaerobic chamber but whose corresponding oxygen-exposed plate did not exhibit any growth. Each colony was added to 15 μL of lysis buffer (TE containing 0.1% Triton-X100), incubated at 95 °C for 15 min, and the solution centrifuged for 10 minutes (3,100 x g at room temperature). A 1 μL aliquot of the supernatant was added to a 20 μL reaction mixture containing 10 μL High-

Fidelity PCR Master Mix with HF Buffer (Phusion), 1 μL of a 10 μM solution of primer Fprau02, 1 μL of a 10 μM solution of primer Fprau07 (*75*) and 7 μL of nuclease-free H_2O. DNA was amplified (initial denaturation for 2.5 minutes at 98 °C, followed by 30 cycles of 98 °C for 10 seconds, 67 °C for 30 seconds and 72 °C for 30 seconds, followed by extension for 5 minutes at 72 °C). An isolate with a positive amplicon was confirmed to be *F. prausnitzii* by performing PCR with primers 8F and 1391R and sequencing of the resulting full-length 16S rDNA amplicon.

Genomic libraries were prepared from four replicate cultures of the colony-purified *F. prausnitzii* isolate using the DNA extraction method and the scaled-down Illumina Nextera XT kit described above. The resulting libraries were sequenced using an Illumina MiniSeq instrument (paired-end 150 nt reads). Nextera adapter sequences were trimmed (cutadapt). The isolate genome was assembled using SPAdes (*60*), initially annotated using Prokka (*47*) and then subjected to *in silico* metabolic reconstructions.

GNOTOBIOTIC PIGLET STUDIES

Experiments involving gnotobiotic piglets were performed under the supervision of a veterinarian using protocols approved by the Washington University Animal Studies Committee.

Preparation of diets – MDCF(PCSB) and MDCF(CS) were produced using ingredients described in **table S18A**. Diets were packed in vacuum-sealed plastic bags (2 kg double-bagged aliquots), sterilized by gamma-irradiation (20-50 kGy) and stored at –20 °C.

Re-deriving piglets as germ-free – The protocol used for generating germ-free piglets was based on our previous publication (*27*) with several modifications. A pregnant domestic sow (mixture of Landrace and Yorkshire genetic backgrounds) that had been artificially inseminated (Duroc breed domestic boar), was delivered one day prior to the date of farrow (i.e., on day 113 of gestation). The sow was sedated with ketamine (20 mg/kg, administrated intramuscularly) and anesthetized with isofluorane (2-3%, delivered by mask). The paralumbar abdominal area was disinfected with povidone-iodine. A local incisional block was achieved using 60-80 mL of 2% lidocaine (subcutaneous injection). Each horn of the bicornate uterus was opened and each piglet was removed from its amniochorionic sac while it was still located in the opened uterine horn. The umbilical cord was tied off and each piglet was passed immediately, prior to its first breath, into and through a sterile tank filled with 2% chlorhexidine (10 second procedure) to prevent contamination with residual viable microbes that might be present on the sow's skin. The tank was connected to a sterile, flexible film 'nursery' isolator so that the piglets could be directly passed into this temporary housing unit. After the Caesarean section, the sow was euthanized according to American Veterinary Medical Association (AVMA) guidelines.

Piglets were revived in the isolator and kept on a heated pad until the remaining piglets in the litter were delivered. Within 24 h, all piglets were transferred from nursery isolators to larger gnotobiotic isolator tubs (Class Biologically Clean Ltd., Madison, WI). Before colonization on postnatal day 4 (see below), the germ-free status of piglets was confirmed by aerobic and anaerobic culture of rectal swabs in LYBHI medium (*73*) before colonization on postnatal day 4. Piglets were group-housed (4 piglets per isolator, with equivalent size range between groups, complying with USDA animal housing regulations). Isolators were maintained at 95-100 °F for the first 7-10 postnatal days, and gradually decreased to 85-90 °F as the thermoregulatory capacity of the animals improved.

Feeding protocol - Piglets were initially bottle-fed with an irradiated sow's milk replacement (Soweena Litter Life, Merrick; catalog number C30287N) that was prepared in 120 g vacuum-sealed sterilized packets (gamma-irradiated with >20 kGy) and reconstituted as a liquid solution in the gnotobiotic isolator (120 g/ L autoclaved water). Piglets were fed at 3-hour intervals for the first 3 postnatal days, at 4-hour intervals from postnatal days 4 to 10, and at 6-hour intervals from postnatal day 10 to the end of the experiment. Introduction of solid foods commenced at postnatal

day 4 and weaning was accomplished by day 14. Each gnotobiotic isolator was equipped with five stainless steel bowls. During the first three days after birth, all five bowls were filled with Soweena. From days 4 to 7, at each feeding, one bowl was filled with an MDCF prototype while the remaining four bowls were filled with Soweena. On day 8, one bowl of milk was replaced with a bowl of water. On day 9, another bowl of milk was replaced with water (i.e., each isolator at each feed contained 2 bowls of water, 2 bowls of Soweena and 1 bowl of MDCF). On day 10, each feed consisted of placement of one bowl of Soweena, two bowls of water, and two bowls of MDCF into the isolator. From day 11 to day 13, only one bowl was provided with Soweena, and the amount of milk added was reduced by one half each day during this period. On day 14, the last bowl of milk was replaced with a bowl of water, thereby completing the weaning process. Health status was evaluated every three to four hours throughout the day and night during weaning. After weaning, three bowls of fresh sterilized water and two bowls of fresh MDCF were introduced into each isolator every 6 hours to enable *ad libitum* feeding. MDCF consumption was monitored by noting the amount of input food required to fill each bowl during a 24-hour period. Piglets were weighed daily using a sling (catalog number 887600; Premier Inc., Charlotte, NC). Environmental enrichment was provided within the isolators including plastic balls for 'rooting' activity and rubber hoses and stainless steel toys for chewing and manipulating. The behavior and health status of the piglets were monitored every day throughout the experiment to ensure their well-being.

Colonizing piglets – Bacterial strains were cultured under anaerobic conditions in pre-reduced MegaMedium (*55*, *57*). An equivalent mixture of each age-/growth-discriminatory strain was prepared by adjusting the volumes of each culture based on optical density (600 nm) readings. An equal volume of pre-reduced PBS containing 30% glycerol was added to the mixture and aliquots were frozen and stored at -80 °C until use. Each piglet received an intragastric gavage (Kendall Kangaroo^{TM} 2.7 mm diameter feeding tube; catalog number 8888260406; Covidien, Minneapolis, MN) of 11 mL of a solution containing a mixture of the bacterial consortium and Soweena (1:10 v/v).

Euthanasia and biospecimen collection - Piglets were fasted for 6 hours, removed from their gnotobiotic isolator, sedated with ketamine (20 mg/kg, administered intramuscularly) and anesthetized with isofluorane (2%, delivered by mask). Euthanasia was performed on experimental day 31 following AVMA guidelines. Blood was collected from the heart after the piglets were anesthetized but prior to administration of pentobarbital. Serum was recovered from clotted blood samples after centrifugation (4000 x g, 10 minutes, 4 °C). Luminal contents were harvested from the distal 5% of the small intestine (ileum), cecum, and distal 10 cm of the colon and flash frozen in liquid nitrogen. Samples of the biceps femoris and liver were placed in liquid nitrogen and stored at -80 °C. The left femur was also obtained at the time of euthanasia; after removing soft tissue and muscle, the bone was wrapped in sterile PBS-soaked gauze and stored at -20 °C.

Micro-computed tomography - Femoral bone was analyzed with a VivaCT 40 instrument (settings 70kVp/114 µA with 300 ms of integration time). The voxel dimension for the scan was 25 μm^3. The epiphyseal plate was used as a 0% reference point. Slices [2274 ± 22 per femur (mean ± SD)] obtained between 40 to 50% of the length of the bone were used for cortical bone analysis (*76*). Images were analyzed using a custom MatLab script based on the 3-D structural measuring method (*77*).

LC-MS/MS-based serum proteomics – The protein concentration of each serum sample was quantified [bicinchoninic acid (BCA) assay, Pierce]. An aliquot containing 500 µg of protein was diluted to 5 µg/µL with 100 mM ammonium bicarbonate (ABC) buffer to a total volume of 100 µL. Samples were further diluted with 100 µL ABC buffer containing 8% sodium deoxycholate (SDC) plus 10 mM dithiothreitol (DTT), pH 8.0, and incubated at 90 °C for 5 minutes. Cysteines

were alkylated/blocked with 15 mM iodoacetamide followed by incubation at room temperature for 20 minutes in the dark. Samples were then loaded onto a 10 kDa MWCO spin filter (Vivaspin500; Sartorius) and centrifuged at 10,000 x g for 20 minutes to concentrate proteins atop the filter. Concentrated proteins were washed with 400 µL ABC buffer, the filter was centrifuged, and proteins were resuspended in 200 µL of ABC buffer containing 10 µg of sequencing-grade trypsin (Sigma Aldrich). Proteolytic digestion atop the filter membrane was allowed to proceed for 4 hours at 37 °C followed by a second application of trypsin (10 µg in 200 µL ABC buffer; overnight incubation). Sample filters were transferred to new 2 mL microfuge tubes and centrifuged at 10,000 x g for 20 minutes to collect tryptic peptides in the flow-though. Peptide samples were acidified with 0.5% formic acid and the resulting sodium deoxycholate precipitate was removed by ethyl acetate extraction (*78*). The peptide-containing aqueous phase was concentrated in a SpeedVac and peptide concentrations were measured by BCA assay.

Peptide samples were analyzed by automated 2D LC-MS/MS using a Vanquish UHPLC with autosampler plumbed directly in-line with a Q Exactive Plus mass spectrometer (Thermo Scientific) outfitted with a triphasic back column [RP-SCX-RP; reversed-phase (5 µm Kinetex C18) and strong-cation exchange (5 µm Luna SCX) chromatographic resins, Phenomenex] coupled to an in-house pulled nanospray emitter packed with 30 cm Kinetex C18 resin. For each sample, peptides (5 µg) were auto-loaded, desalted, separated and analyzed across two successive salt cuts of ammonium acetate (50 and 500 mM), each followed by a 105-minute organic gradient (*79*). Eluting peptides were measured and sequenced by data-dependent acquisition on the Q Exactive instrument.

MS/MS spectra were searched with MyriMatch v.2.2 (*80*) against the *Sus scrofa* proteome (derived from genome assembly 11.1, GCA_000003025.6, January 2017) concatenated with common protein contaminants. Reversed-sequence entries were also provided to estimate FDR. Peptide-spectrum matches (PSM) were required to be fully tryptic with any number of missed cleavages; a static carbamidomethylation of cysteines (+57.0214 Da) and variable modifications of oxidation (+15.9949 Da) on methionine. PSMs were filtered using IDPicker v.3.0 (*81*) with an experiment-wide FDR controlled at < 1% at the peptide-level. Peptide intensities were assessed by chromatographic area-under-the-curve (label-free quantification option in IDPicker). To remove cases of extreme sequence redundancy, the *Sus scrofa* proteome was clustered at 90% sequence identity (UCLUST) (*82*), and peptide intensities were summed to their respective protein groups/seeds to estimate overall protein abundance. Protein abundance distributions were then log-transformed, normalized across samples (LOESS and mean-centered), and missing values imputed to simulate the mass spectrometer's limit of detection.

SUPPLEMENTARY RESULTS

The plasma proteomes of children with SAM prior to and after treatment with conventional therapeutic foods

Analysis of the proteomic dataset revealed significant correlations between plasma proteins and anthropometric indices (see **table S3A** for p-values). WHZ scores were positively correlated with circulating levels of the soluble proteolytic cleavage product of the membrane-bound growth hormone receptor [GHR, also known as growth hormone binding protein (GHBP), r = 0.6]. Approximately 50% of GH is bound to GHBP, which serves to prolong its half-life and modulate its biological activity (*83*). GHBP is increased in obese adults and reduced after weight loss (*84*). In the children treated for SAM, plasma GHBP was also positively correlated with the adipokine leptin (Spearman r=0.6), consistent with the notion that increased fat mass, driven by nutritional recovery, leads to changes in leptin and GH signaling. WHZ scores were also positively

correlated with downstream GH-responsive biomarkers, including lumican, extracellular matrix protein 1 (ECM1) and fibronectin (*85*).

A number of plasma proteins exhibited strong negative correlations with WHZ scores, including angiotensinogen (AGT; Spearman r = -0.70), a key component of the renin-angiotensin system (RAS) that regulates blood pressure and other aspects of cardio-metabolic function. Malnutrition has been reported to induce a pro-inflammatory state with increased expression of RAS components, analogous to responses observed in mouse models of diet-induced obesity (*86*). There was also an inverse correlation between plasma levels of C-reactive protein (CRP), an acute phase reactant and biomarker of systemic inflammation, and WHZ scores (Spearman r = -0.56).

Plasma proteins with significant correlations with plasma NEFA, ketones, lactate, glucose, triglycerides, branched-chain amino acids and C3 acylcarnitine are listed in **table S3B.** They include growth differentiation factor 15/macrophage inhibitory cytokine -1 (GDF15/MIC-1), which was significantly correlated with NEFA (r=0.78) and ketones (r=0.58), and negatively correlated with WAZ (r = -0.69; **table S3A**). This TGF-β superfamily member is implicated in anorexia and muscle wasting associated with cancer (*87*), and with chronic heart failure in children (*88*).

Circulating IGFs (IGF-1 and IGF-2) are complexed with binding proteins (IGFBPs), primarily IGFBP-3. Binding to IGFBPs affects the half-life of IGFs and their interactions with extracellular matrix components and cell surface receptors (*89*). The IGFBPs have unique functions and are regulated in distinct ways. Unlike IGFBP-3, IGFBP-1 and IGFBP-2 are suppressed by GH and are implicated in adaptive changes in glucose and lipid metabolism (*90*). Pappalysin-1 (pregnancy-associated plasma protein-A, PAPP-A) is a metalloprotease that selectively cleaves IGFBP-2, -4, and -5, resulting in release of sequestered IGF, thereby promoting its ability to bind to its receptor (*91*). In mice, overexpression of PAPP-A in osteoblasts results in a marked increase in the rate of bone formation (*92*), while overexpression in muscle increases skeletal muscle weight and fiber area (*93*). Stanniocalcin-1 (STC1) is a potent physiological inhibitor of IGFBP proteolysis by PAPP-A (*94*). Transgenic mice engineered to overexpress STC1 exhibit severely reduced growth (*95*).

In children treated for SAM, levels of IGFBP-1 and IGFBP-2 were positively correlated with levels of NEFA (r = 0.74 and 0.68, respectively) and ketones (r = 0.49 and 0.60) while IGFBP-3 exhibited an inverse relationship with both analytes (r = -0.62 and -0.52), and IGFBP-4 with ketones (r = -0.56) [**table S3B**]. IGFBP-4, another regulatory component of the GH-IGF axis positively correlated with WHZ, is highly expressed in adipocytes and is a proposed regulator of adipose tissue development and maintenance (*96*). PAPP-A was positively correlated with levels of branched-chain amino acids (valine r = 0.59, leucine/isoleucine r = 0.50). STC1 was strongly positively correlated with NEFA (r = 0.70) and negatively correlated with ponderal growth (WAZ; r = -0.48). In summary, these results reveal that elevated plasma levels of IGFBP-1 and IGFBP-2 are associated with the acutely malnourished state, whereas IGFBP-3 and IGFBP-4 are associated with the metabolic normalization and ponderal growth that characterize the recovery phase of treatment. The observed changes in PAPP-A, together with reciprocal changes in its physiological inhibitor, STC1, may serve to regulate IGF-1 bioavailability, thereby affecting a range of anabolic processes (*97*).

Correlations between abundances of age-discriminatory taxa and plasma proteins – We calculated Spearman's rank correlations between (i) the abundance of OTUs identified at enrollment (time point S1 in **Fig. 1A**), at discharge (S5) and 6 months following discharge (S11), and (ii) levels of plasma proteins at the corresponding time points (see **table S3C** for all OTU-plasma protein correlations >0.5 or <-0.5). Integrating the plasma proteomics dataset with changes in the relative abundances of OTUs allowed us to identify statistically significant

correlations between age- and growth-discriminatory bacterial taxa and mediators of host biological state/functions (**table S3C**). For example, *Faecalibacterium prausnitzii* (OTU 514940), the taxon with the highest feature importance score in the sparse RF-derived model of microbiota maturation in healthy members of the Mirpur birth cohort (*2*), exhibited strong positive correlations with a number of proteins involved in or regulated by GH signaling, including GHR, lumican, fibronectin, and ECM1 (**table S3C**). Multiple age-discriminatory OTUs had significant negative correlations with GDF15, including *F. prausnitzii, Clostridiales* sp.*, Dorea longicatena, Dorea formicigenerans, Blautia* sp., *Eubacterium desmolans,* and two members of Ruminococcaceae (*Ruminococcaceae* sp. and *R. torques*). *F. prausnitzii* and a number of other age-discriminatory strains were also significantly negatively correlated with plasma CRP levels (**table S3C**).

Bifidobacterium longum is a dominant member of the microbiota of breastfed infants; its presence is associated with numerous beneficial effects on the gut barrier and immune function (*98*). *B. longum* (OTU 559527) has the third highest feature importance in the sparse Bangladeshi RF-derived model and is a key component of the 15-member network of co-varying bacterial taxa ('ecogroup') described in (*31*). It is also responsive to MDCF formulations containing the four lead complementary food ingredients tested in gnotobiotic mice and piglets as well as in children with MAM (**fig. S7B** in this report and **Fig. 3** in (*31*)).

B. longum OTU 559527 was significantly correlated with 114 plasma proteins—the greatest number of significant correlations among the age-discriminatory OTUs (**table S3C**). These proteins are involved in a wide range of biological processes. The two most strongly correlated proteins, legumain (an asparaginyl endopeptidase) and matrix metalloproteinase-2 (MMP-2/gelatinase A) which is proteolytically cleaved and activated by legumain, are involved in remodeling the extracellular matrix. Among its other functions, MMP2 has been shown to cleave the chemokine CCL7 (MCP-3), converting it from a leukocyte chemoattractant to an antagonist, reducing cell infiltration, and dampening inflammation (*99*). Plasma levels of three cadherins (2, 3 and 6) that function as calcium-dependent cell adhesion molecules were positively correlated with *B. longum* abundance.

B. longum was also correlated with plasma levels of WNT1-inducible-signaling pathway protein 3 (WISP-3), a member of the CCN family of secreted proteins that regulate cell proliferation/survival, migration and adhesion, and differentiation in connective tissues. WISP-3 is secreted by chondrocytes where it can act in an autocrine fashion to induce collagen and aggrecan production and promote expression of superoxide dismutase (*100*). WISP-3 contains an IGFBP-like motif and has been demonstrated to modulate IGF-1 signaling in breast cancer (*101*).

B. longum was positively correlated with plasma levels of CDON (cell adhesion molecule-related/down-regulated by oncogenes). CDON and BOC (Brother of CDON) promote Hedgehog signaling through calcium-dependent interactions with Hedgehog ligands as co-receptors on the surface of target cells (*102*). There is considerable cross-talk between the Hedgehog pathway and Notch, WNT, EGF, FGF, TGF-beta and BMP signaling cascades. A number of these pathways are prominently represented by proteins that show significant correlations with the abundance of *B. longum* **(table S3C**), including positive correlations with jagged-2 (JAG2), a Notch ligand involved in hematopoiesis, and BMP6, which is involved in growth of bone and cartilage (*103*). Another Notch ligand, delta-like protein 4 (DLL4), exhibits a strong negative correlation with *B. longum*. Inflammation has been reported to upregulate DLL4 in endothelial cells. In conjunction with IL-6 (which is also negatively correlated with *B. longum*), DLL4 promotes differentiation of blood monocytes into proinflammatory M1 macrophages (*104*). Blockade of DLL4 produces a marked reduction in inflammatory T cell responses and associated tissue damage (*105*).

In the SAM study, plasma levels of TNFSF15/TL1A (tumor necrosis factor ligand superfamily member 15) were inversely correlated with *B. longum* abundance. TNFSF15/TL1A

is a member of the TNF superfamily that binds to death domain receptor 3 (DR3, TNFRSF25), activates NF-κB, and co-stimulates IFN-γ production in T cells (*106*). TNFSF15/TL1A and DR3 expression are increased in T cells and macrophages in the gut mucosa of patients with inflammatory bowel disease (*107*).

Biomarkers of systemic inflammation are a hallmark of children with undernutrition and growth faltering (*108*). *F. prausnitzii* (OTU 514940, 514523, 370287), *D. formicigenerans* (1076587), a weaning-phase *Bifidobacterium* sp. (484304), and *Ruminococcus gnavus* (360015) were all negatively correlated with C-reactive protein (CRP). Other acute phase proteins were also negatively correlated with the abundance of *F. prausnitzii* OTUs, including serum amyloid A-1 protein (SAA1) and complement C2. These opsonins target microbes for clearance and aid in the recruitment of immune cells to sites of infection. The negative correlation between these proteins and *F. prausnitzii*, *D. formicigenerans*, *R. gnavus* and the OTU ranked second in feature importance (1078587; *Clostridiales* sp.) in the sparse RF-derived model of microbiota maturation suggests that (i) a deficiency of these weaning-phase taxa may be conducive to developing or sustaining a state of local and systemic inflammation in children with SAM, and/or (ii) such a state reduces their fitness. A causal role for *F. prausnitzii* in suppressing gut inflammation is supported by the finding that it produces anti-inflammatory compounds that have protective effects in mouse models of DNBS- and DSS-induced colitis through inhibition of the NF-κB pathway (*109, 110*).

MMP12 is a macrophage-specific metalloelastase whose expression was strongly correlated with the abundance of *F. prausnitzii* and several other age-discriminatory taxa including *Clostridiales* sp*., D. formicigenerans, Blautia* sp. and *R. torques*. MMP12 binding to the IκBα promoter is essential for transcriptional up-regulation of IκBα, which is required for IFNα secretion by leukocytes and antiviral immunity. Outside of the cell, MMP12 cleavage also forms a feedback loop to down-regulate IFNα by degrading it, thereby limiting systemic effects of prolonged IFNα elevation (*111*). A similar negative feedback role has been described for macrophage MMP12 in the proteolysis and inactivation of pro-inflammatory CXC and CC cytokines released by LPS stimulation of polymorphonuclear leukocytes (*112*).

Plasma levels of heat shock proteins Hsp90aa1 and Hsp90ab1 were strongly negatively correlated with *F. prausnitzii* levels in the gut microbiota. The observed relationship between *F. prausnitzii* and Hsp90 in plasma suggest that there is an extracellular or secreted form of Hsp90. There is a growing appreciation of the role of extracellular heat shock proteins as 'danger' signals that stimulate innate and adaptive immune responses (*113-115*).

Microbial RNA-Seq analysis of the effects of the initial MDCF prototype versus Milk-Suji/ Khichuri-Halwa (MS/KH) in gnotobiotic mice colonized with the defined community

To further characterize the responses of the 14-member consortium of age-discriminatory bacterial strains to the initial MDCF prototype, 5-week-old germ-free C57Bl/6J mice (n = 6 animals/treatment group; 3 cages of dually-housed mice/group) were placed on MDCF or MS/KH and three days later gavaged with the 14-member consortium. All mice were monotonously fed their designated diets for an additional 40 days. There were no significant differences in microbial community biomass [2.9±0.8 μg DNA/g cecal contents (MDCF) versus 2.7±0.3 μg/g (MS/KH); p=0.48, Mann-Whitney test]. COPRO-Seq analysis disclosed that as in the previous experiment shown in **Fig. 3**, the weaning-phase age-discriminatory strains *R. torques, R. obeum, F. praunitzii* and *D. longicatena* exhibited the largest and most statistically significant elevations in their relative abundances in the ceca of MDCF- compared to MS/KH-fed mice (Mann-Whitney test; **table S9C**).

Microbial RNA-Seq datasets were generated from cecal contents and the results were interpreted based on KEGG and SEED-based annotations of the 40,735 predicted protein-coding

genes present in consortium members, plus *in silico* predictions of the abilities of the bacterial strains to produce, utilize and/or share nutrients (**tables S6D,E**, **table S11**).

Three weaning-phase age-discriminatory strains - *F. prausnitzii*, *R. obeum* and *R. torques* - had the greatest number of genes with statistically significant differences in their expression between the two diets [320, 308 and 184, respectively; **table S11C(i)**). Given its high feature importance scores in the Bangladeshi and other RF-derived models of microbiota development (see **table S4B,C** and (*31*)), its consistent increase in fitness with MDCF compared to MS/KH across experiments, its multiple predicted nutrient requirements (**table S6D,E**), and the large number of genes that are differentially expressed between the two diets, *F. praunitzii* represents an attractive 'model' for investigating how the MDCF prototype affects weaning-phase age-discriminatory microbial targets.

Among the *F. prausnitzii* genes with significantly higher levels of expression in the ceca of mice fed MDCF versus MS/KH were an alpha-glucosidase belonging to CAZyme glycoside hydrolase family (GH) 31 (EC:3.2.1.20; encoded by FPSSTS7063_00084), a GH 13 oligo-1,6-glucosidase (EC:3.2.1.10; FPSSTS7063_00083), a glycosyltransferase (GT) family 35 starch/glycogen phosphorylase (EC:2.4.1.1; FPSSTS7063_00079), and three linked genes in the maltose/maltodextrin transport system (FPSSTS7063_00085-87) (**table S11A**). Increased expression of *F. prausnitzii* genes encoding enzymes that hydrolyze 1,4- and 1,6-alpha-glucosidic linkages suggests that starch serves as a preferred substrate. In contrast, *R. torques* exhibits increased expression of the *agaEFG-rafA* genes involved in uptake and hydrolysis of alpha-galactosides such as raffinose (RTSSTS7063_01731-01735) (**table S11A)**; this pathway is absent from *F. prausnitzii*. These differentially expressed genes might reflect adaptations to chickpea and banana, two of the three complementary food leads represented in the initial MCDF prototype; both complementary foods are rich in raffinose and stachyose while banana is also enriched in resistant starch (*116*, *117*). In contrast, a set of 20 *F. prausnitzii* genes represented in several predicted operons involved in utilization of hexuronates (D-glucuronic and D-galacturonic acids) exhibit 2- to 23-fold lower levels of expression in mice fed the MDCF diet compared to MS/KH (**table S11A**). These latter findings are consistent with observed differences in the availability of these nutrients in the cecum (e.g., glucuronic acid is present at lower levels in germ-free mice fed MDCF versus MS/KH; **table S10A**).

Effects of diet in mice colonized with a post-SAM MAM donor microbial community

Microbial RNA-Seq analysis of the cecal community - The mcSEED categories 'Amino Acid Metabolism', 'Vitamin and Cofactor Metabolism', 'Carbohydrate Utilization', and 'Fermentation Products' and their subsystems/pathway modules were assigned ranks, calculated by dividing the total number of differentially expressed genes in a category/subsystem/pathway module by the total number of genes in that category/subsystem/pathway module. Higher rank corresponds to a greater proportion of differentially expressed genes in that category/subsystem/pathway module. The results **(Fig. 4B**, **table S15D,E**) disclosed 'Amino Acid Metabolism' as the category with highest rank. Eighteen of the 20 subsystems/pathway modules belonging to this category, including all of the biosynthetic subsystems/pathway modules, were upregulated in mice consuming Mirpur(PCSB) or Mirpur(P) compared to Mirpur-18; the most upregulated subsystem/pathway module was 'isoleucine, leucine, valine biosynthesis' (60 genes significantly upregulated, and 4 significantly downregulated with Mirpur(PCSB) versus Mirpur-18; P-values based on gene set enrichment analysis). This subsystem/pathway module was also the most upregulated on Mirpur(P) compared to Mirpur-18 (27 genes compared to zero downregulated).

Metabolic features of the cecal community - Targeted mass spectrometry of cecal contents disclosed that levels of all 15 free amino acids measured were significantly higher in colonized

mice consuming the unsupplemented compared to supplemented Mirpur-18 diets. These differences were not observed in their germ-free counterparts (see **fig. S5A** and **table S15B**; the latter also includes data on the levels of products of microbial metabolism of aromatic amino acids, carbohydrates, short chain fatty acids and B vitamins). The origin of these colonization-dependent effects on cecal amino acid levels is unknown; for example, higher levels of free amino acids in the unsupplemented Mirpur-18 could reflect a more active proteolysis of dietary proteins or less active salvage/degradation of amino acids by the microbial community. However, the results are consistent with the observed differential expression patterns; i.e., accumulation of amino acids typically leads to repression of bacterial regulons controlling their biosynthesis. Diet-associated differences in levels of cecal branched-chain and other amino acids in colonized animals were not accompanied by significant differences in levels of the corresponding serum amino acids (**table S16**).

The effects of MDCFs on expressed functions of a F. prausnitzii strain recovered from the transplanted post-SAM MAM microbiota - *F. prausnitzii* OTU 514940 was a prominent member of the cecal microbiota in these mice (15-17% mean relative abundance across the different diets; **Fig. 4C** and **table S15C(ii)**). We cultured and sequenced the genome of an isolate representing this OTU from the transplanted post-SAM MAM donor community. Strain JG_BgPS064 contains 2,824 predicted genes; it shares 2268 genes with the SSTS_Bg7063 isolate used for the diet oscillation screens of complementary foods in gnotobiotic mice (**table S6F**). Of the 266 genes involved in the metabolic reconstructions described for SSTS_Bg7063, there are 248 orthologs in JG_BgPS064; the 19 missing genes are predicted to be non-essential for their respective metabolic pathways because for each there is an iso-functional paralog or alternative pathway. The JG_BgPS064 strain is predicted to produce all amino acids except His and Trp (although its genome contains committed His and Trp salvage ABC transporters). In contrast to the SSTS_Bg7063 strain, this isolate possesses intact LeuC-LeuD genes involved in leucine biosynthesis and thus is likely a Leu prototroph. Metabolic reconstructions suggest JG_BgPS064 can utilize galactose and beta-galactosides, glucose and beta-glucosides, maltose and maltodextrin, fructose and fructooligosaccharides, sialic acids, N-acetylgalactosamine, hexuronic acids (glucoronate, galacturonate), lacto-N-biose (only galactose moiety), and rhamnogalacturonides (only glucoronate moiety). Additionally, this isolate possesses fermentative pathways for production of butyrate, formate, and acetate.

We mapped reads from the microbial RNA-Seq dataset onto its sequenced genome to identify the effects of diet on expression of genes belonging to various mcSEED categories and subsystems/pathway modules (**table S15F**). Among the 271 differentially expressed genes, the largest number belong to the mcSEED category 'Amino Acid Metabolism' (35 genes), followed by 'Carbohydrate Utilization' (18 genes). All 35 differentially regulated genes involved in amino acid metabolism exhibited increased expression in the supplemented compared to unsupplemented Mirpur-18 diet treatment groups, with the most significantly upregulated subsystem/pathway module being 'isoleucine, leucine, valine biosynthesis' and 'glutamate biosynthesis'. No significant differences in expression of these pathways were observed between the two supplemented diets (**table S15F**).

Mass spectrometry of cecal contents from germ-free and colonized mice revealed microbiota- and diet-dependent effects on butyrate and acetate levels (significantly greater in colonized mice consuming the supplemented diets; **fig. S5B** and **table S15B**). Cecal butyrate was also positively correlated with the relative abundance of *F. prausnitzii* (Pearson $r = 0.68$, $p=0.0074$). Acetate production has been linked to resistance to certain enteropathogens (*118*), while butyrate is an important contributor to gut mucosal barrier function (*119*) (See the maintext for a discussion of how RNA-Seq analysis of laser capture microdissected small intestinal (jejunal) mucosa provided

evidence for microbiota- and diet-dependent increases expression of genes involved in barrier function).

Effects on gut cell populations - The different diets produced no statistically significant differences in the number of small intestinal goblet cells or Paneth cells, or crypt depth to villus height ratios between mice colonized with the post-SAM MAM donor microbiota (Student's t-test). However, analysis of hematoxylin- and eosin-stained sections revealed a trend toward an increase in the number and size of submucosal lymphoid aggregates in the proximal and middle thirds of the small intestine in post-SAM MAM microbiota colonized animals consuming Mirpur(PCSB) compared to the other treatment groups (**fig. S6A**). Immunostaining disclosed that these aggregates are B-cell dominant, with T-cell zones and associated rare IgA-positive plasma cells (**fig. S6B**). The number and size of these submucosal lymphoid aggregates in Mirpur(PCSB) treated mice harboring the post-SAM MAM microbiota was not significantly different from the number and size of aggregates in conventionally-raised mice harboring a native mouse microbiota fed a standard mouse chow (Kruskal-Wallis test with Dunn's correction for multiple comparisons; **fig. S6A**).

Microbial RNA-Seq and mass spectrometry of cecal contents from gnotobiotic piglets
Comparative microbial RNA-Seq of cecal contents harvested from piglets consuming MDCF(CS) or MDCF(PCSB) diets identified 2,021 differentially expressed genes with a complex distribution over 12 strains (**table S18C,D**); 117 of these genes, from eight strains, mapped to mcSEED categories and associated subsystems/pathway modules.

Table S19A compares amino acid, mono- and disaccharide, organic acid, and short chain fatty acid levels in the ceca of piglets as a function of diet. Of the 24 carbohydrates measured, only fructose exhibited a significant difference between the two groups [higher in MDCF(PCSB)-treated animals; P = 0.02, unpaired t-test]. MDCF(PCSB) consumption was associated with lower cecal lactate and pyruvate levels (P = 0.06 and 0.002, respectively), in concert with marked increases in the late TCA cycle intermediates malate and fumarate (P = 0.0002 and 0.005, respectively), but not early intermediates (citrate, succinate, and α-ketoglutarate). These findings are consistent with a decrease in glycolytic metabolism of glucose and an increase in oxidative metabolism of glucose and other fuels (**table S19A**).

Quantitative proteomic and metabolomic analyses of serum from gnotobiotic piglets
Because the aptamers used for quantitative proteomics analysis of human plasma samples do not have reported specificities for the corresponding porcine protein orthologs, we used mass spectrometry to compare the serum proteomes of piglets. Blood was obtained from animals after the 6-hour fast, just prior to euthanasia. We did not attempt to deplete serum samples of abundant proteins prior to two-dimensional LC-MS/MS to avoid introducing biases in our analysis. Thirty-eight of the 398 detected proteins exhibited significant differences in their abundances between the two diet treatment groups (**table S20**).

Serum levels of EFEMP1 (fibulin-like extracellular matrix protein 1) in piglets consuming MDCF(PCSB) were 4.6-fold higher than in their MDCF(CS) fed counterparts (**Fig. 5E, table S20**). Genome-wide association studies have identified EFEMP1 as significantly associated with height in children (*121*). Mice with engineered deficiency of Efemp1 exhibit significant reductions in body mass and bone density (*122*).

Three other serum proteins that were significantly increased in MDCF(PCSB)-treated piglets are orthologs of human proteins significantly correlated with anthropometric and/or metabolic features in the SAM study: serpin family A member 5 (SERPINA5), complement factor I (CFI) and fetuin-B (FETUB) (**Fig. 5E, table S20**). In children recovering from SAM, levels of SERPINA5 were positively correlated with plasma C3-acylcarnitine, a marker of branched-chain

amino acid oxidation (Spearman r = 0.52) **(table S3B)**. While circulating serum levels of amino acids in piglets were comparable in the two treatment groups (with the exception of Trp and Met which were increased in the MDCF(PCSB) group; **table S19B**), serum C3-acylcarnitine concentrations were significantly higher in the faster growing MDCF(PCSB)-treated animals than in those consuming MDCF(CS) (**Fig. 5F)**. In the human study, plasma levels of fetuin-B, a member of the cystatin family of cysteine protease inhibitors produced by the liver, and CFI were positively correlated with ponderal growth [fetuin-B, r = 0.59 (WAZ) and 0.54 (WHZ); CFI, r = 0.44 (WAZ); **table S3A**]. Fetuin-B has been linked to glucose homeostasis (*123*) and fatty acid utilization (*124*). In addition to its correlation with anthropometric measures of growth, fetuin-B was also positively correlated with the relative abundance of *R. gnavus* (OTU 360015) and *F. prausnitzii* (OTU 514940) in fecal samples collected during the course of the SAM study [r = 0.46 and 0.66, respectively (**table S3C**). Note that MDCF(PCSB) significantly augments the representation of *R. gnavus*, a growth-discriminatory bacterial species **(Fig. 5D)**].

As in humans, the pig genome encodes seven IGFBP orthologs and an ALS (acid-labile subunit). ALS forms a ternary complex with IGF-1 and IGFBPs, prolonging the half-life of IGF-1, and in a rat model plays a role in growth promotion (*120*). IGF-1 was below the limits of detection in our LC-MS/MS analysis of non-depleted sera, and no significant differences were noted in the one IGFBP that was identified (IGFBP-2). However, levels of ALS in animals fed MDCF(PCSB) were 2.3-fold higher than in those consuming MDCF(CS) (**table S20**).

SUPPLEMENTARY FIGURES

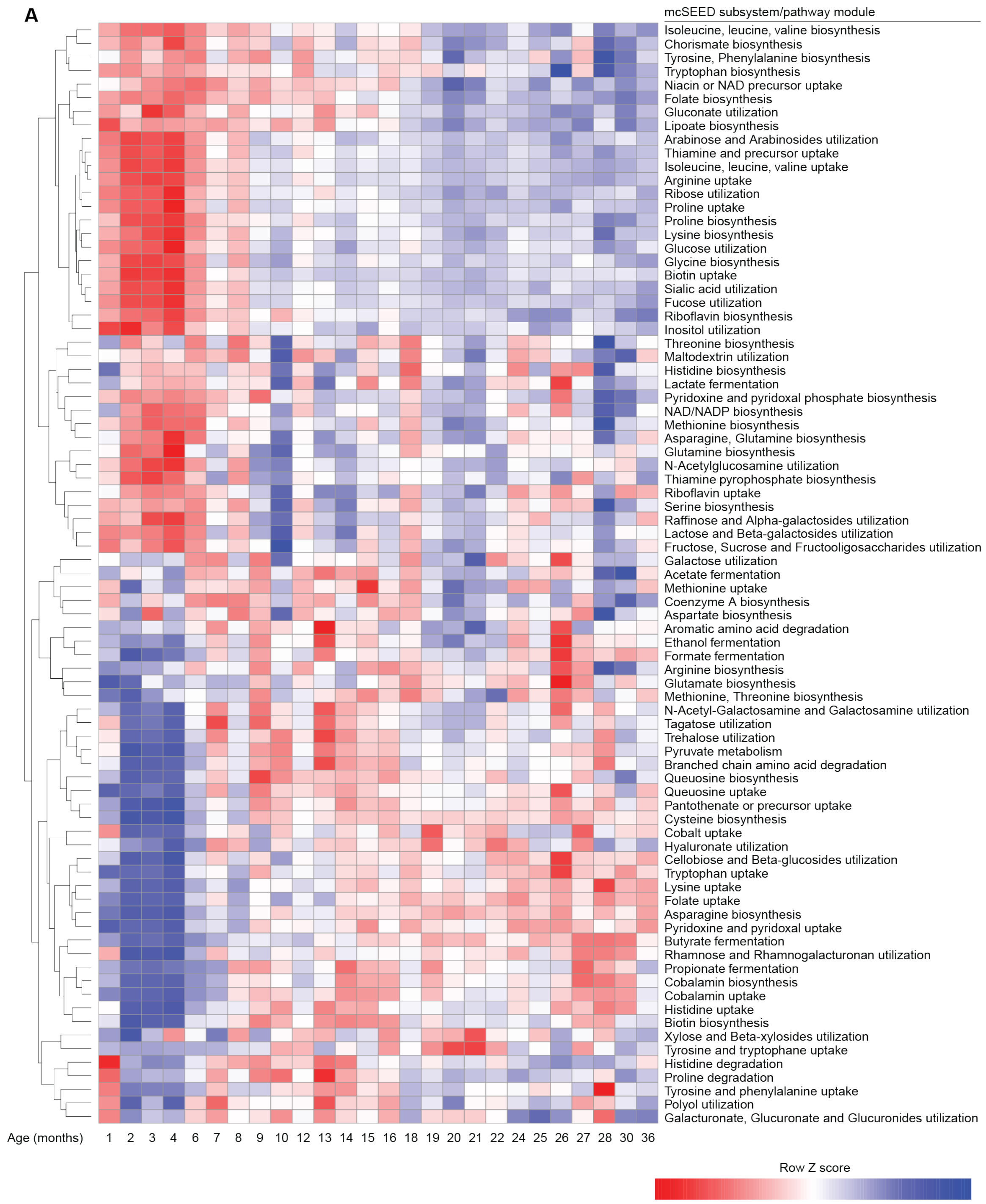

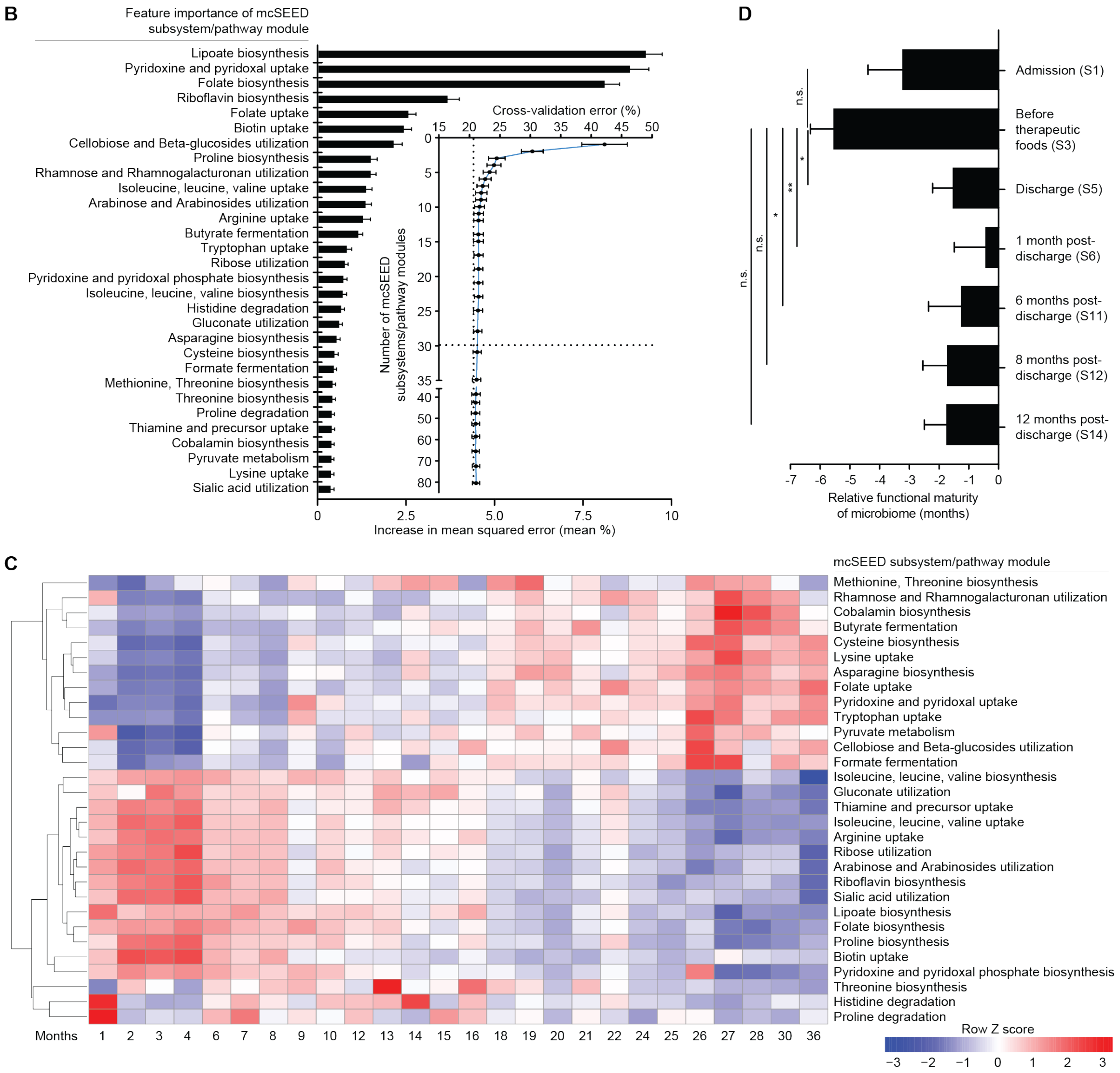
B
Feature importance of mcSEED subsystem/pathway module
Lipoate biosynthesis
Pyridoxine and pyridoxal uptake
Folate biosynthesis
Riboflavin biosynthesis
Folate uptake
Biotin uptake
Cellobiose and Beta-glucosides utilization
Proline biosynthesis
Rhamnose and Rhamnogalacturonan utilization
Isoleucine, leucine, valine uptake
Arabinose and Arabinosides utilization
Arginine uptake
Butyrate fermentation
Tryptophan uptake
Ribose utilization
Pyridoxine and pyridoxal phosphate biosynthesis
Isoleucine, leucine, valine biosynthesis
Histidine degradation
Gluconate utilization
Asparagine biosynthesis
Cysteine biosynthesis
Formate fermentation
Methionine, Threonine biosynthesis
Threonine biosynthesis
Proline degradation
Thiamine and precursor uptake
Cobalamin biosynthesis
Pyruvate metabolism
Lysine uptake
Sialic acid utilization
0
2.5
5.0
7.5
10
Increase in mean squared error (mean %)
Cross-validation error (%)
15 20 25 30 35 40 45 50
Number of mcSEED subsystems/pathway modules
0 5 10 15 20 25 30 35 40 50 60 70 80
D
Admission (S1)
Before therapeutic foods (S3)
Discharge (S5)
1 month post-discharge (S6)
6 months post-discharge (S11)
8 months post-discharge (S12)
12 months post-discharge (S14)
n.s.
*
**
*
n.s.
n.s.
-7 -6 -5 -4 -3 -2 -1 0
Relative functional maturity of microbiome (months)
C
mcSEED subsystem/pathway module
Methionine, Threonine biosynthesis
Rhamnose and Rhamnogalacturonan utilization
Cobalamin biosynthesis
Butyrate fermentation
Cysteine biosynthesis
Lysine uptake
Asparagine biosynthesis
Folate uptake
Pyridoxine and pyridoxal uptake
Tryptophan uptake
Pyruvate metabolism
Cellobiose and Beta-glucosides utilization
Formate fermentation
Isoleucine, leucine, valine biosynthesis
Gluconate utilization
Thiamine and precursor uptake
Isoleucine, leucine, valine uptake
Arginine uptake
Ribose utilization
Arabinose and Arabinosides utilization
Riboflavin biosynthesis
Sialic acid utilization
Lipoate biosynthesis
Folate biosynthesis
Proline biosynthesis
Biotin uptake
Pyridoxine and pyridoxal phosphate biosynthesis
Threonine biosynthesis
Histidine degradation
Proline degradation
Months 1 2 3 4 6 7 8 9 10 12 13 14 15 16 18 19 20 21 22 24 25 26 27 28 30 36
Row Z score
−3 −2 −1 0 1 2 3

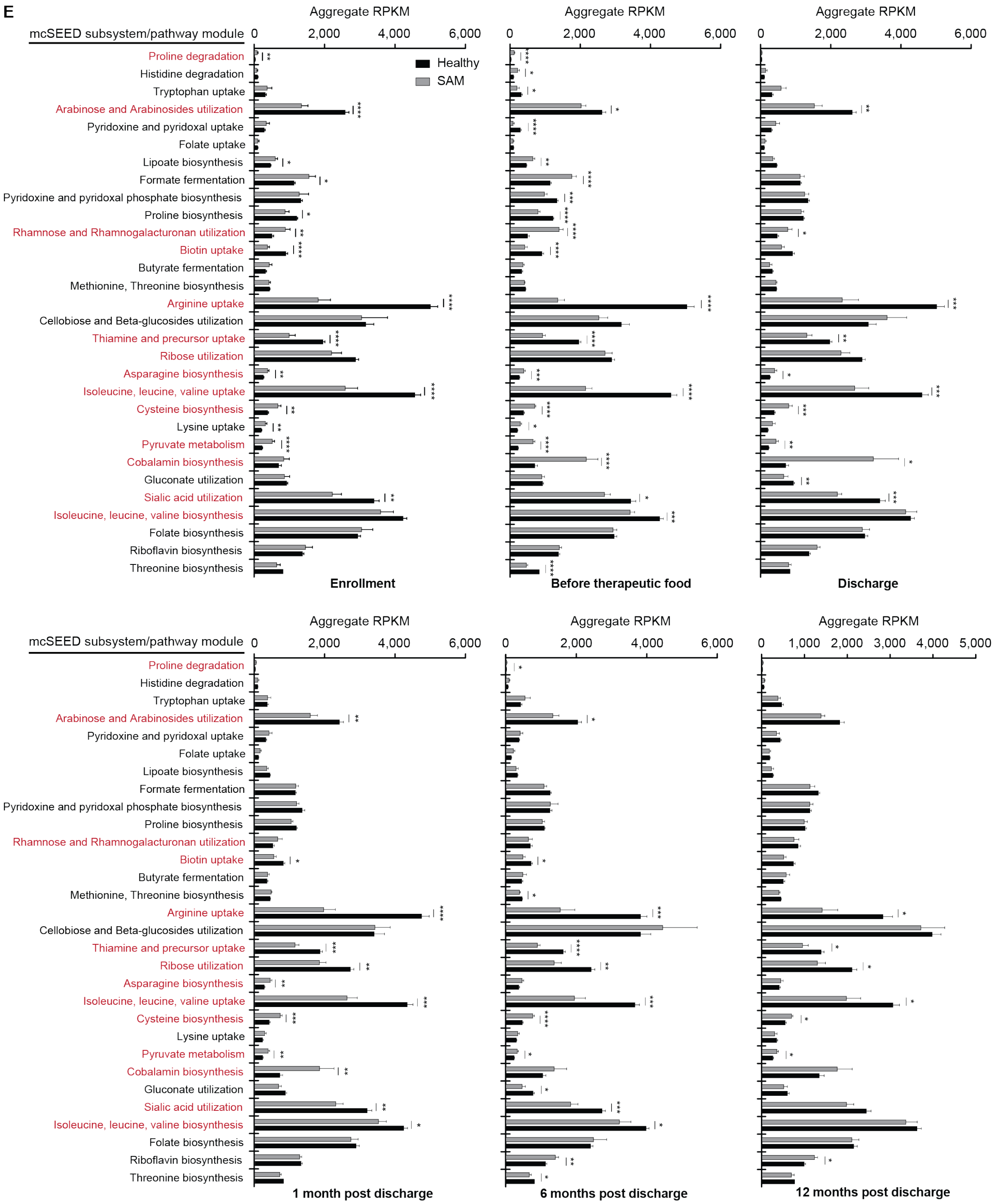

fig. S1. Comparison of the fecal microbiomes of children with healthy growth phenotypes and those treated for SAM. (**A**) Changes in representation of mcSEED subsystems/pathway modules in the fecal microbiomes of healthy Mirpur children (n = 30) sampled during the first three postnatal years. (**B**) Ranked feature importance of the age-discriminatory mcSEED subsystems/pathway modules comprising the RF-derived model of gut microbiome development in Mirpur infants/children with healthy growth phenotypes. The mcSEED subsystem/pathway module 'lipoate biosynthesis' had the highest feature importance score; lipoate/lipoic acid is an

essential cofactor of dehydrogenase enzymes including branched-chain ketoacid dehydrogenase, a regulator of branched-chain amino acid catabolism. The representation of subsystems/ metabolic pathway modules involved in amino acid metabolism (including branched-chain amino acids and tryptophan), B vitamin metabolism, carbohydrate metabolism and fermentation are important contributors to the accuracy of the model. (**C**) Heatmap of the changes in representation of the top 30 most age-discriminatory mcSEED subsystems/pathway modules in the fecal microbiomes of the healthy Mirpur children. (**D**) Relative functional maturity of the microbiomes of children hospitalized with SAM at enrollment, just prior to treatment, at discharge, and at 1-, 6-, 8- and 12-months post-discharge, calculated using the RF-derived model. ns, not significant; ***,** P<0.05; (one-way ANOVA, Tukey's multiple comparisons test). Note that the statistically significant improvement in relative functional maturity at 1-month post-discharge compared to that at enrollment is similar to the improvement observed in MAZ (**Fig. 1B**). Moreover, there is a significant positive correlation between MAZ and relative functional microbiome maturity (r = 0.55, P<0.0001). However, this improvement was not sustained; there is no statistically significant difference in this parameter between initiation of treatment and 8- or 12-months post-discharge (one-way ANOVA followed by Tukey's multiple comparisons test; adjusted P-values = 0.077 and 0.083, respectively). (**E**) Differences in the representation of mcSEED subsystems/pathway modules in the fecal microbiomes of children prior to, during and after treatment for SAM compared to healthy children. Abundances of the 30 most age-discriminatory mcSEED subsystems/pathway modules in 15 individuals treated for SAM compared to age-matched healthy individuals. Subsystems/pathway modules whose representation is significantly different between healthy and SAM children for at least three of the six time points are highlighted in red. Mean values ± SD are shown. *, P<0.05; **, P<0.01; ***, P<0.001; ****, P<0.0001 (Mann-Whitney test with FDR correction).

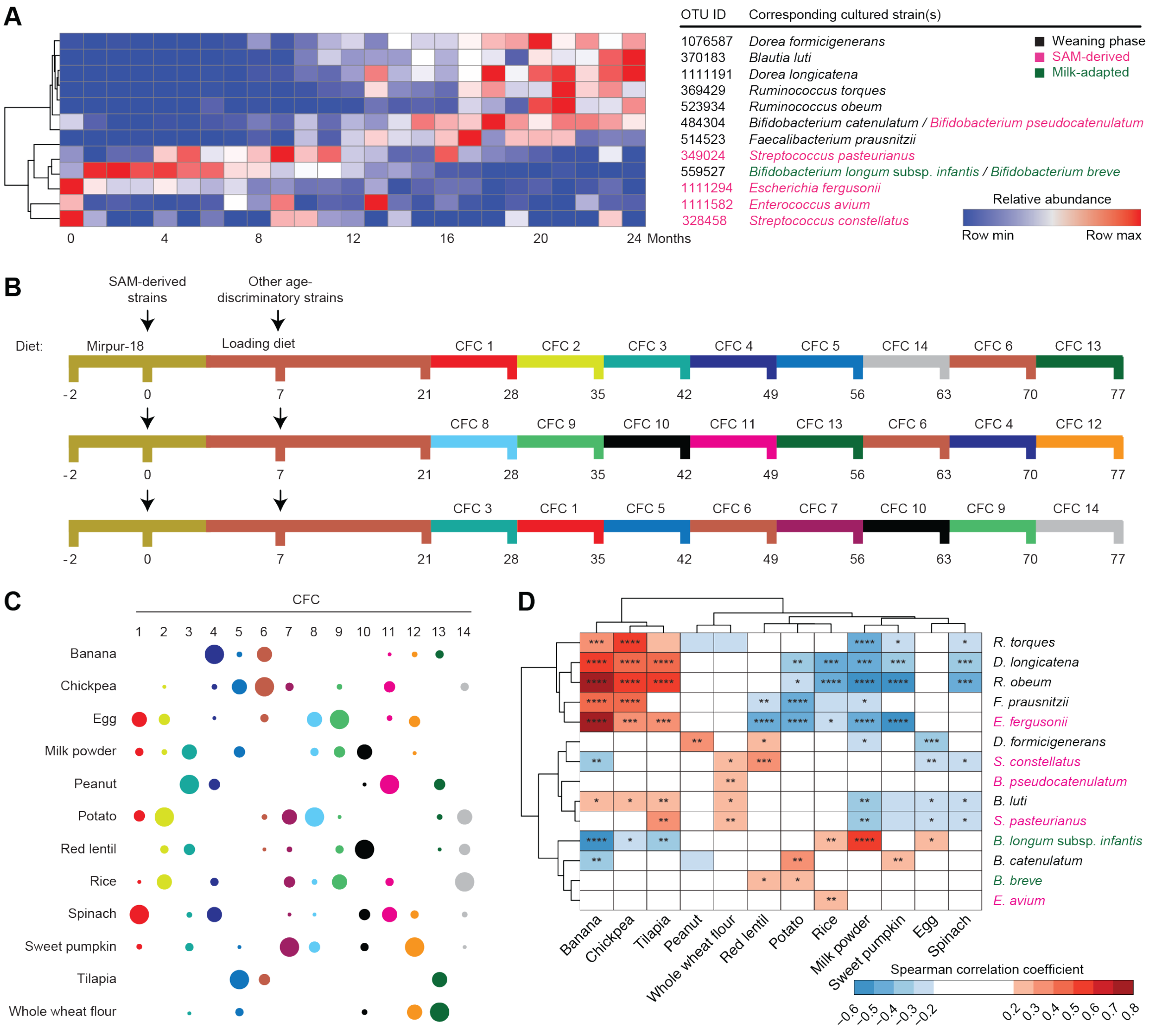

fig. S2. Diet oscillation study to identify complementary foods that selectively increase the relative abundances of weaning-phase age-discriminatory bacterial strains in gnotobiotic mice. (**A**) Changes in the abundances of OTUs in the developing microbiota of healthy members of the Mirpur birth cohort that correspond to cultured strains used to colonize gnotobiotic mice. Rows are arranged based on unsupervised hierarchical clustering of the strains' temporal abundance profiles. Strains in red font were obtained from a 24-month-old donor with SAM. *B. pseudocatenulatum* and *B. catenulatum* have the same V4-16S rDNA sequences as *B. breve* and *B. longum*. *B. longum* subsp. *infantis* was distinguished from *B. longum* subsp. *longum* based on its five genomic loci encoding numerous carbohydrate active enzymes (*13*). (**B,C**) Experimental design for diet oscillation study. Fourteen unique complementary food combinations (CFCs) were designed by random sampling of 12 ingredients (panel B). The composition of these CFCs and their order of administration were based on the following considerations. *First,* every CFC contained six different complementary food ingredients at six different levels, one of which was dominant (see panel C; the size of the circle in the bubble plot indicates the relative level of the ingredient). In addition to these six ingredients, each CFC also contained bovine milk powder and soybean oil to reflect the fact that children in Mirpur with MAM or SAM are typically treated with therapeutic foods that contain these ingredients. The Spearman correlation coefficient

between the amounts of any two ingredients across all diets was minimized so that the abundances of targeted taxa could be clearly related to a given ingredient (**table S8A**). Each selected formulation was prepared in ways that reflected common culinary practices in Mirpur, processed as a homogeneous blend, extruded into pellets, and sterilized by irradiation. *Second,* each group of mice received a different weekly sequence of the different diet formulations in order to identify ingredient-microbe interactions that are robust to order of diet presentation (i.e., to avoid hysteresis effects). *Third,* no group of mice received a formulation dominated by a particular complementary food ingredient more than once during the course of the experiment, although a given ingredient was represented in multiple formulations at different concentrations. *Fourth,* while no two groups of mice were fed diets dominated by the same complementary food ingredient within the same week, replication was achieved by administering it as the dominant ingredient to different groups at least twice over the course of an experiment (n = 8-12 mice/diet). Shotgun sequencing (COPRO-Seq) of total DNA isolated from fecal samples collected on the last day of each 1-week diet block was used to determine the relative abundance of each community member (see **table S9A**). Fourteen of the 16 cultured isolates successfully colonized all animals. *Streptococcus gordonii* and *Streptococcus salivarius* were the exceptions: they were below the limit of detection in fecal samples obtained at the 9 time points surveyed per mouse; their corresponding OTUs have feature importance scores of 30 and 23, respectively, in the sparse 30 OTU Bangladeshi RF-derived model of normal gut community development. (**D**) Hierarchical clustering of Spearman's rank correlation coefficients between the relative abundance of each bacterial strain in the fecal microbiota of recipient mice and levels of the indicated complementary food ingredient. *, P<0.05; **, P<0.01; ***, P<0.001; ****, P<0.0001 (Benjamini-Hochberg-corrected P-values).

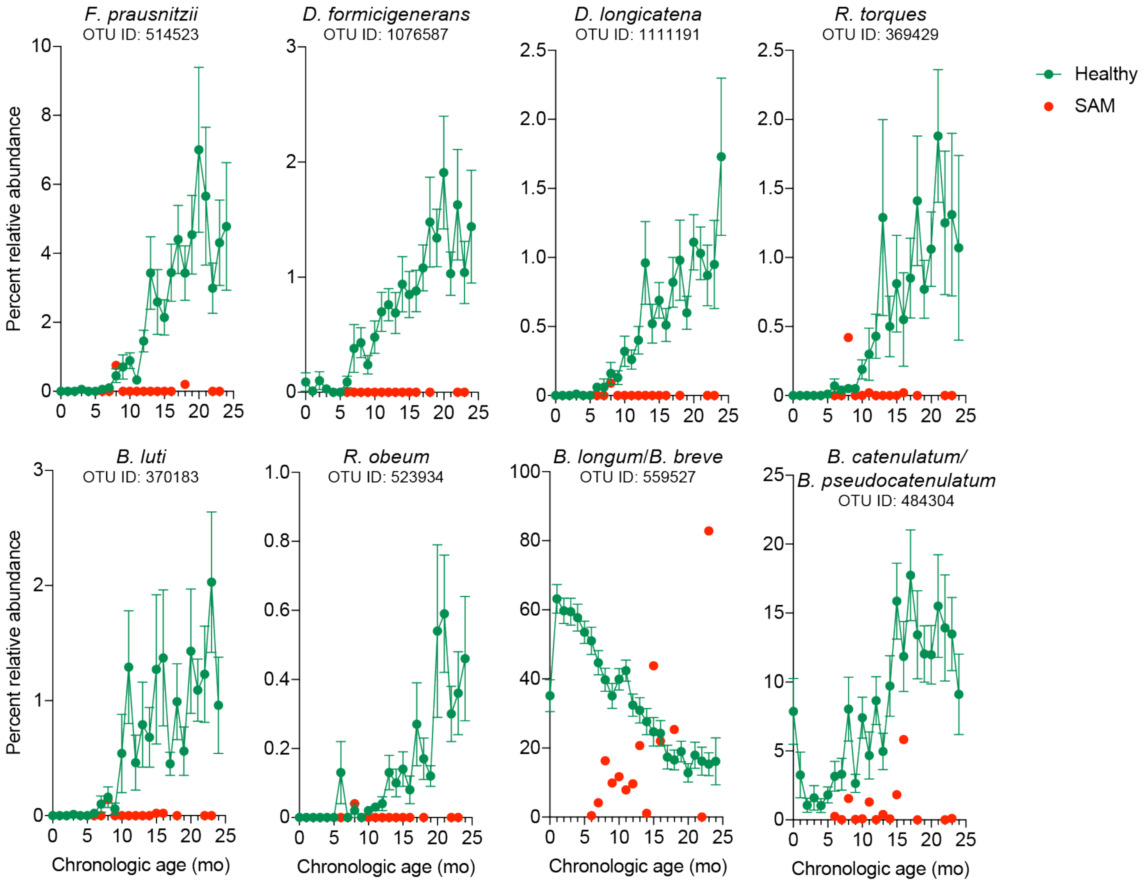

fig. S3. Relative abundances of OTUs corresponding to cultured age-discriminatory strains in the fecal microbiota of children living in Mirpur. Abundances of the indicated taxa were determined by V4-16S rDNA sequencing of fecal samples collected monthly from healthy children in the birth cohort (mean values ± SD are shown) and from children with SAM prior to treatment. Note that 29 of these 38 children were enrolled in the present study while 9 were from a prior SAM cohort described in (*2*). Neither *B. breve* and *B. longum,* nor *B. pseudocatenulatum* and *B. catenulatum* can be distinguished from one another based on their V4-16S rDNA sequences.

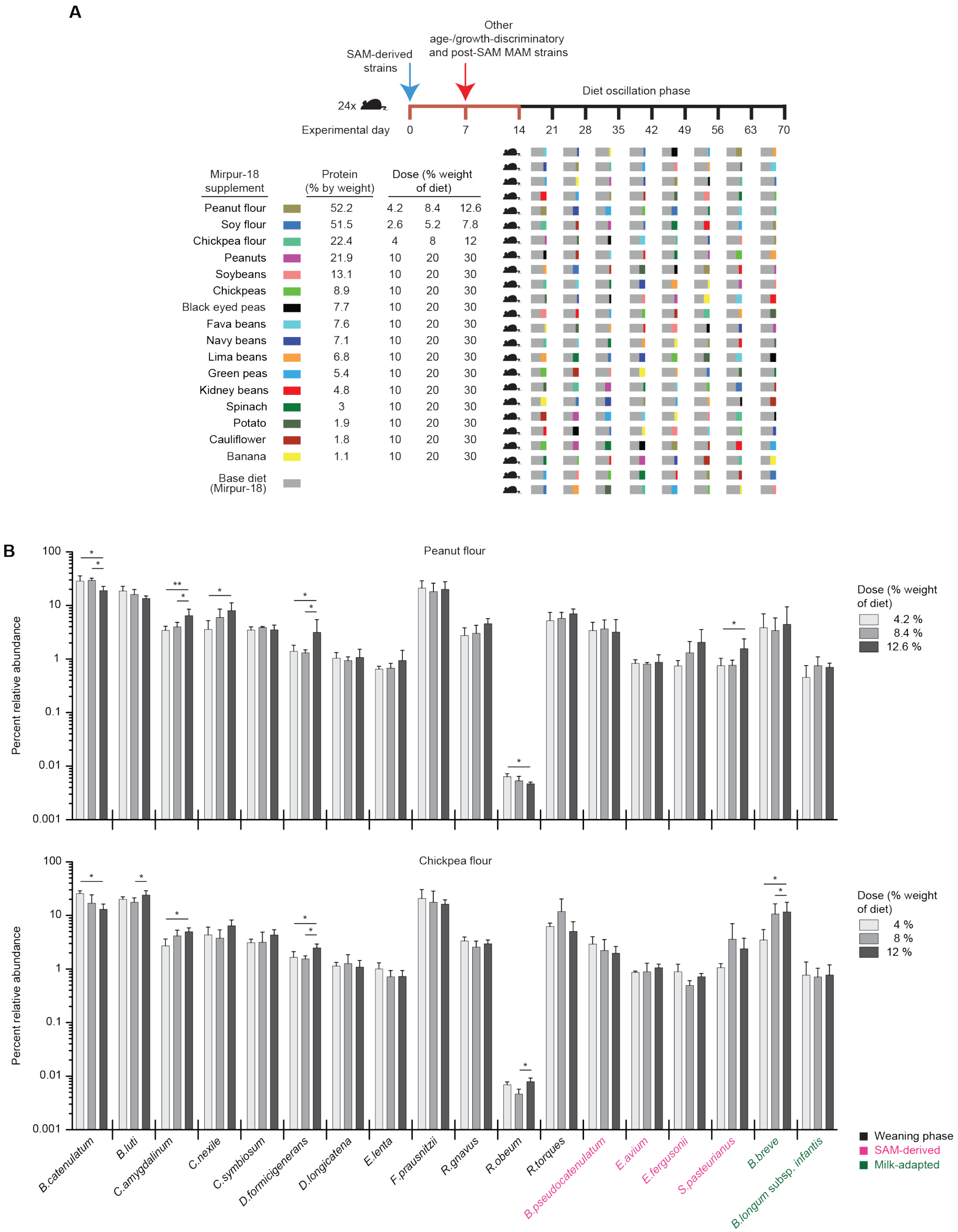

fig. S4. Effects of supplementing the Mirpur-18 diet with 16 plant-derived complementary food ingredients in mice colonized with an 18-member consortium of age-/growth-discriminatory bacterial strains. (**A**) Experimental design. The diagram portrays the three different doses of each complementary food ingredient (colored according to the key) added to

the base Mirpur-18 diet (grey) and the different order of presentation of the different diets to different mice. Each diet was given monotonously for 1 week. (**B)** Relative abundances of strains (log scale) in the fecal microbiota of mice fed three different doses of peanut flour and chickpea flour (see **table S13C**). Mean values ± SD are shown. *, P<0.05; **, P<0.01 (Kruskal-Wallis test followed by post-hoc Dunn's test; Benjamini-Hochberg-corrected P-values; n = 4 mice/dose/diet).

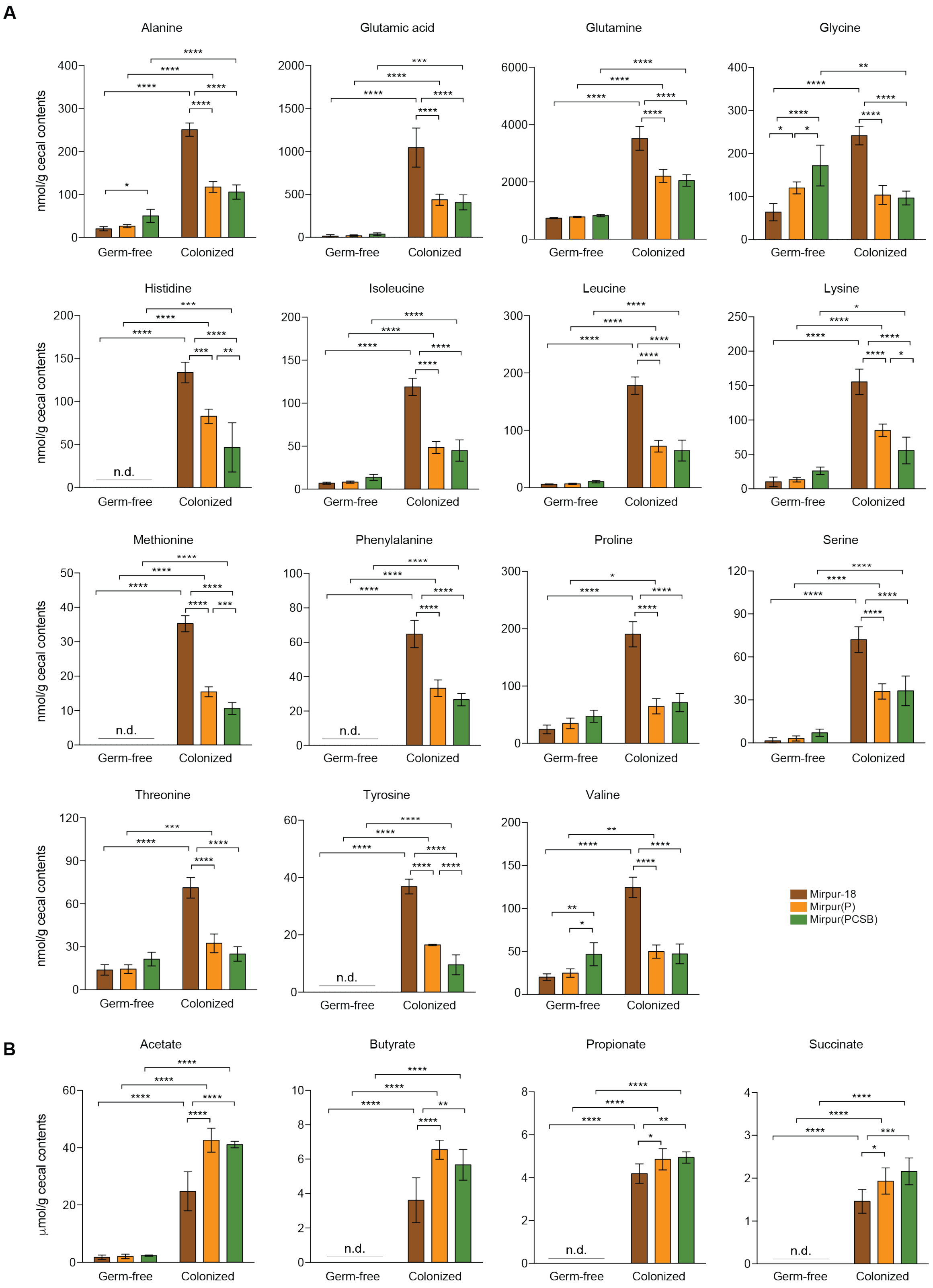
A
Alanine
Glutamic acid
Glutamine
Glycine
Histidine
Isoleucine
Leucine
Lysine
Methionine
Phenylalanine
Proline
Serine
Threonine
Tyrosine
Valine
nmol/g cecal contents
Germ-free
Colonized
n.d.
Mirpur-18
Mirpur(P)
Mirpur(PCSB)
B
Acetate
Butyrate
Propionate
Succinate
μmol/g cecal contents

fig. S5. Targeted mass spectrometry of cecal contents harvested from gnotobiotic mice colonized with a post-SAM MAM donor microbiota and from germ-free controls. Levels of amino acids (**A**) and short chain fatty acids (**B**) in animals fed unsupplemented or supplemented Mirpur-18 diets (see **table S15B**). n.d., not detected. *, P<0.05; **, P<0.01, ***, P<0.001, ****, P<0.0001 (two-way ANOVA followed by Tukey's multiple comparisons test).

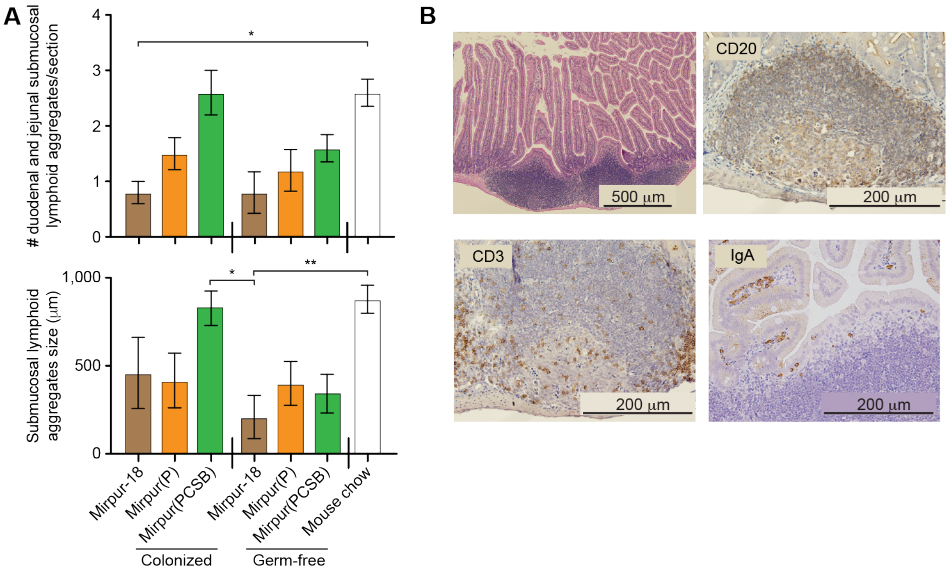

fig. S6. Diet- and colonization-dependent increases in submucosal lymphoid aggregates in the small intestines of gnotobiotic mice. Mice colonized with the post-SAM MAM donor microbiota and control germ-free animals were fed unsupplemented or supplemented Mirpur-18 diets. (**A**) Lymphoid aggregates present in hematoxylin- and eosin-stained sections from the proximal third of small intestinal segments SI-1 and SI-2 (see **Fig. 4D**) were counted for all mice in all six treatment groups (n = 5 animals/group). 'Size' represents the largest cross-sectional diameter for each aggregate in each section scored from each small intestinal segment from each animal. Mean values ± SD are shown. (**B**) Histo- and immunohistochemical characterization of a representative jejunal lymphoid aggregate from a representative colonized mouse fed the Mirpur(PCSB) diet. The upper left portion of the subpanel notes specificity of the antibodies used for staining the section. *, P<0.05; **, P<0.01 (Kruskal-Wallis test with Dunn's correction for multiple comparisons).

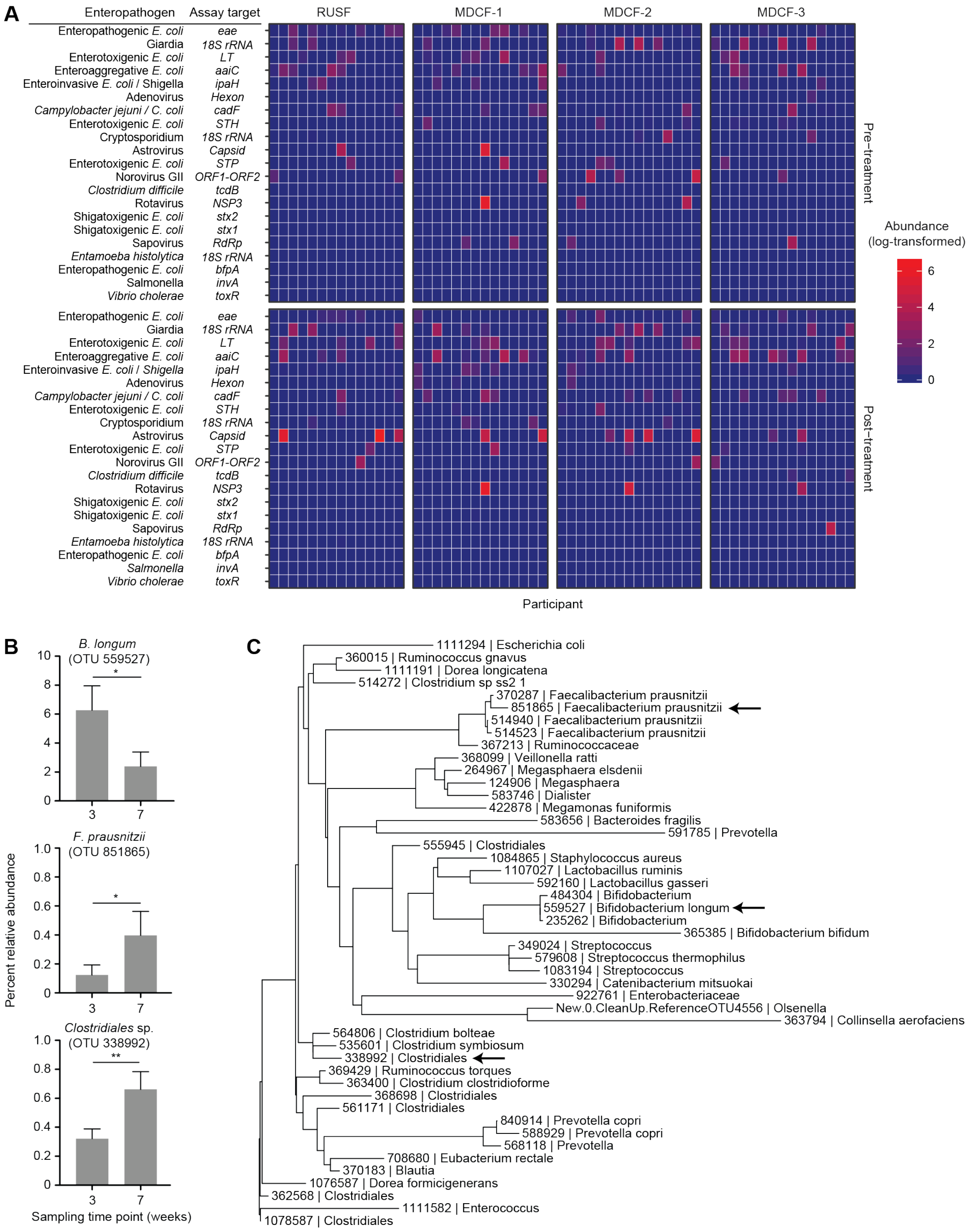

fig. S7. Analysis of the fecal microbiota of children in the MDCF trial. (**A**) Quantification of enteropathogen burden in children before and after treatment. Results are expressed as log-transformed pg genomic DNA (bacteria and parasites), copy number (RNA viruses), and mass per cell lysate mass (Adenovirus). (**B**) Bacterial OTUs with significant changes in their percent relative abundances in the fecal microbiota of children in the MDCF-2 treatment arm. Mean

values ± SEM are shown. *P<0.05; **, P<0.01 (paired t-test). (**C**) Phylogenetic tree that includes the OTUs shown in panel B (arrows).

SUPPLEMENTARY TABLES

table S1. The effects of three therapeutic foods in 54 children with SAM subjected to a 12-month follow-up. (A) Composition of therapeutic foods used. **(B)** Clinical metadata. The eight individuals highlighted in boldface contributed serial plasma samples for quantitative aptamer-based proteomic analyses.

table S2. Targeted mass spectrometry-based metabolic profiling of plasma samples obtained from children before and at two time points after treatment for SAM.

table S3. Correlations between plasma protein levels, host features and bacterial OTUs in the fecal microbiota of participants in the SAM study. Significant Spearman correlations ($r>0.5$ or $r<-0.5$) between plasma protein levels and **(A)** anthropometry, **(B)** selected plasma metabolites (see **Fig. 2**) and (**C**) bacterial taxa.

table S4. Random Forests (RF)-derived sparse 2-year model of gut microbiota development in healthy members of a Bangladeshi (Mirpur) birth cohort. (A) Data used to generate RF-derived model **(B)** Top 60 OTUs with the highest feature importance in the RF-derived model. **(C)** Sparse 30 OTU RF-derived model generated from healthy members of the Mirpur birth cohort (n=25 individuals; 539 fecal samples). OTUs are ranked in descending order of their importance to the accuracy of the model. The x-axis plots the increase in mean-squared error when abundance values from each OTU are randomly permuted. The inset shows the cross-validation curves that result from reducing the number of 97% ID OTUs used for model training.

table S5. Age-discriminatory bacterial OTU abundance, microbiota maturity (MAZ), mcSEED subsystem/pathway module representation, and relative functional maturity of fecal microbiomes of Bangladeshi children with healthy growth phenotypes versus those with SAM prior to and following treatment. (A) Percent relative abundances of age-discriminatory taxa (from left to right in order of feature importance in the sparse RF-derived model of normal microbiota maturation) in samples from children with SAM. Calculated MAZ scores are provided. **(B)** Summary of shotgun sequencing dataset. **(C)** Aggregate representation of mcSEED subsystems/pathway modules (ordered from left to right by feature importance in the sparse RF-derived model of normal microbiome maturation) in each fecal DNA sample, expressed as reads per kilobase per million reads mapped (RPKM). Relative functional microbiome maturity is shown for children in the SAM study and the 20 healthy children in the test set for the sparse RF-derived model.

table S6. Bacterial strains introduced into gnotobiotic animals. (A) Bacterial strains highlighted in grey were cultured from the fecal microbiota of Bangladeshi donors and used for the diet oscillation study shown in **fig. S2** and to characterize the initial MDCF prototype (**Fig. 3**). All 20 listed strains were gavaged into germ-free mice to test the effects of supplementing Mirpur-18 with 16 plant-derived complementary food ingredients (**fig. S4**). **(B)** The 14-member consortium introduced into gnotobiotic piglets. **(C)** Genome characteristics of bacterial strains cultured from the fecal microbiota of Bangladeshi donors. **(D)** Summary of *in silico* predictions of the ability of the 14 age-discriminatory strains, introduced into mice in the experiments described in **fig. S2** and **Fig. 3,** to produce amino acids, utilize mono- and disaccharides, and generate short chain fatty acids. Results are summarized in the form of a binary phenotype matrix. In the case of amino acids, "1" denotes a predicted prototroph and "0" an auxotroph as inferred from *in silico* reconstruction of the respective pathway (subsystem/pathway module) in

the analyzed genomes. In the case of carbohydrates, '1' and '0' refer to a strain's predicted ability or inability, respectively, to utilize the indicated mono- or disaccharide while '1*' indicates that the catabolic pathway is present but no specific transporter was identified. Binary phenotypes for fermentation end products are related to a strain's predicted ability to produce the indicated compound. Amino acids are shown using standard 3-letter code. Abbreviations for carbohydrates: (Ara)n, Arabinosides; (GlcA)n, Glucuronides; (Mal)n, Maltodextrin; (Xyl)n, Xylosides; Aga, Alpha-galactosides; Ara, Arabinose; Bga, Beta-galactosides; Bgl, Beta-glucosides; Chb, Chitobiose; ddGlcA, unsaturated glucuronate; FOS, Fructooligosaccharides; Fru, Fructose; Fuc, Fucose; Gal, Galactose; GalA, Galacturonate; GalN, Galactosamine; GalNAc, N-Acetylgalactosamine; Glc, Glucose; GlcA, Glucuronate; GlcNAc, N-Acetylglucosamine; Gnt, Gluconate; Hyl, Hyaluronate; Ino, Inositol; Lac, Lactose; Lnb, Galacto-N-biose; ManNAc, N-Acetylmannosamine; Mel, Melibiose; Mtl, Mannitol; NANa, Sialic acids; Raf, Raffinose; Rha, Rhamnose; Rhi, Rhamnogalacturonides; Rib, Ribose; Scr, Sucrose; Srt, Sorbitol; Tag, Tagatose; Tre, Trehalose; Xlt, Xylitol; and Xyl, Xylose. **(E)** Details of metabolic reconstructions. **(i)** Carbohydrate metabolic pathways. **(ii)** Fermentation pathways. **(iii)** Amino acid metabolic pathways. **(iv)** Central carbon metabolic pathways. **(F)** Comparison of the genomes and metabolic reconstructions of the different *F. prausnitzii* strains used in these experiments. **(G)** Metabolic reconstructions for bacterial strains introduced into gnotobiotic piglets.

table S7. 16S rDNA dataset and associated metadata for 38 children from Mirpur with SAM sampled prior to treatment (see fig. S3)

table S8. Composition of diets administered to gnotobiotic mice in the initial *in vivo* screen of CFCs (see fig. S2). (A) Pairwise testing for collinearity between food ingredients in CFCs. **(B)** Ingredient composition of CFCs. **(C)** Nutrient content of CFC diets calculated from the USDA database. **(D)** Components of additional diets. **(E)** Direct nutritional analysis of diets.

table S9. COPRO-Seq datasets from experiments involving gnotobiotic mice colonized with the defined consortium of age-discriminatory strains. (A) *In vivo* screen involving diet oscillation with various CFCs. Percent relative abundances of strains in fecal microbiota (see **fig. S2**). **(B)** Percent relative abundances of strains in the cecal microbiota of mice monotonously fed the initial MDCF prototype versus Milk Suji/Khichuri-Halwa (MS/KH) (see **Fig. 3**). **(C)** Percent relative abundances of strains in the cecal microbial community of mice monotonously fed the MDCF prototype versus MS/KH in a repeat experiment (see *Supplementary Results*).

table S10. Diet- and colonization-dependent effects on levels of cecal metabolites in mice colonized with the defined consortium of age-discriminatory strains and monotonously fed the initial MDCF prototype versus Milk Suji/Khichuri-Halwa (MS/KH) (see **Fig. 3). (A)** Carbohydrates. **(B)** Short-chain fatty acids. **(C)** Amino acids. **(D)** Aromatic amino acid metabolites.

table S11. Differentially expressed genes in cecal contents of gnotobiotic mice colonized with the 14-member community and monotonously fed the initial MDCF prototype or Milk Suji/Khichuri-Halwa (MS/KH). (A) Genes in mcSEED-based metabolic reconstructions and their corresponding DESeq2-normalized expression data. **(B)** Genes not encompassed by the mcSEED subsystems/pathway modules and their corresponding DESeq2-normalized expression. **(C)** Gene set enrichment analysis of KEGG pathways whose expression changes significantly as a function of diet treatment.

table S12. Diet- and colonization-dependent effects on levels of host metabolites (see Fig. 3). (A) Amino acids in serum. **(B)** Amino acids in liver. **(C)** Amino acids in gastrocnemius muscle. **(D)** Acylcarnitines in serum. **(E)** Acylcarnitines in liver. **(F)** Acylcarnitines in gastrocnemius muscle. **(G)** AcylCoAs in liver. **(H)** Organic acids in liver. **(I)** Conventional metabolites in serum.

table S13. Testing 16 plant-derived complementary food ingredients in gnotobiotic mice colonized with an 18-member consortium of age- and growth-discriminatory bacterial taxa. (A) Composition of diets used in the screen. **(B)** Protein quality of the 16 plant-derived CF ingredients, and the number of weaning-phase age-discriminatory taxa whose abundances were promoted by each ingredient. (**C**) COPRO-Seq analysis of the effects of complementary food ingredients on the percent relative abundances of consortium members.

table S14. A screen of the effects of complementary food ingredients on age-discriminatory strains present in 15 intact uncultured microbiota samples from children enrolled in the SAM study. (A) Composition of diets used to screen the microbiota after transplantation into gnotobiotic mice. **(B)** V4-16S rDNA-based analysis of the representation of 30 age- and 3 growth-discriminatory taxa in groups of gnotobiotic mice harboring one of 15 different transplanted microbiota samples tested, as a function of diet treatment, including the number of age-discriminatory taxa detected and overall transplantation efficiency.

table S15. Microbial responses in gnotobiotic mice colonized with a representative post-SAM MAM donor microbiota and fed the Mirpur-18 diet with or without complementary food supplementation. (A) Composition of the three diets. **(B)** Targeted MS analysis of amino acids, aromatic amino acid metabolites, mono- and disaccharides, short-chain fatty acids, and B vitamins in cecal contents recovered from germ-free and colonized mice as a function of their diet treatment. **(C)** V4-16S rDNA analyses of (i) the biogeography of community structure associated with mucosa harvested by laser capture microdissection (LCM) from different small intestinal segments, and (ii) fecal microbiota (collected at 21 days post-gavage, dpg), and cecal microbiota (dpg 25), as a function of diet treatment. Age-/growth-discriminatory taxa are shown in boldface. **(D)** Abundance (RPKM) of the 30 most age-discriminatory mcSEED subsystems/pathway modules represented in the cecal microbiomes of mice subjected to the three different diet treatments. **(E)** Microbial RNA-Seq analysis of genes in mcSEED subsystems/pathway modules that exhibit significant diet-associated differences in their expression in the cecal microbiomes of colonized gnotobiotic mice subjected to the different diet treatments. **(F)** Subsystems/pathway modules and associated genes in the *F. prausnitzii* JG_BgPS064 genome that exhibit significant differences in their expression in the cecal microbiomes of colonized gnotobiotic mice subjected to the different diet treatments.

table S16. Targeted MS analysis of amino acids in the sera of germ-free mice and mice colonized with a post-SAM MAM donor microbiota as a function of their diet treatment.

table S17. RNA-Seq analysis of laser capture microdissected small intestinal mucosa, harvested from gnotobiotic mice, as a function of diet and colonization status. (A) Differentially expressed pathways in different diet contexts in mice colonized with the post-SAM MAM donor microbiota and in germ-free controls. **(B)** All differentially expressed genes in one or more group comparisons.

table S18. The effects of two MDCF formulations on the structure and expressed functions of a 14-member consortium of age-/growth-discriminatory bacterial strains in gnotobiotic

piglets. (A) Composition of MDCF(PCSB) and MDCF(CS) compared to other diets. **(B)** COPRO-Seq analysis of community structure along the length of the gut. **(C)** Microbial RNA-Seq analysis of the cecal meta-transcriptome.

table S19. The effects of two MDCF formulations on the metabolic features of gnotobiotic piglets colonized with the 14-member consortium of age-/growth-discriminatory bacterial strains. (A) Levels of amino acids, carbohydrates, organic acids and short-chain fatty acids in the cecal contents of piglets treated with the two MDCF prototypes. **(B)** Levels of conventional metabolites, amino acids and tryptophan and tryptophan-related metabolites in serum.

table S20. Mass spectrometry-based proteomic analysis of sera obtained from piglets treated with the two MDCF prototypes.

table S21. Clinical data from the MDCF trial in children with MAM. (A) Organoleptic acceptability of the four diets tested**. (B)** MDCF/RUSF consumption during the trial. **(C)** Dietary diversity at baseline and at the end of the study. **(D)** Incidence of morbidities during the trial. **(E)** Anthropometry. **(F)** Enteropathogen load in children with primary MAM studied in the MDCF trial, healthy controls, and those with SAM. Summary statistics are shown by MDCF trial treatment arm or by group (healthy or SAM).

table S22. Metadata associated with fecal and plasma samples collected from children in the MDCF trial, healthy controls and children with SAM.

table S23. Plasma proteome analysis of healthy versus SAM children, and the response of children with primary MAM to MDCFs. (A) Plasma proteins with significant differences in their abundances in healthy children compared to those with SAM. The top 50 proteins whose levels were significantly higher in healthy individuals are highlighted in green. The top 50 proteins significantly higher in individuals with SAM are highlighted in red. **(B)** Plasma proteins with significant fold-changes in abundance from beginning to end of treatment in the four arms of the MDCF trial. **(C)** Gene Ontology Pathways whose representation in the plasma proteome of children in the MDCF-2 treatment arm changes significantly (ranked in order of statistical significance of enrichment as determined by SetRank).

References and Notes

1. T. Ahmed, M. Mahfuz, M. M. Islam, D. Mondal, M. I. Hossain, A. S. Ahmed, F. Tofail, S. A. Gaffar, R. Haque, R. L. Guerrant, W. A. Petri, The MAL-ED cohort study in Mirpur, Bangladesh. *Clin. Infect. Dis.* **59**, S280–S286 (2014). doi:10.1093/cid/ciu458 Medline

2. S. Subramanian, S. Huq, T. Yatsunenko, R. Haque, M. Mahfuz, M. A. Alam, A. Benezra, J. DeStefano, M. F. Meier, B. D. Muegge, M. J. Barratt, L. G. VanArendonk, Q. Zhang, M. A. Province, W. A. Petri Jr., T. Ahmed, J. I. Gordon, Persistent gut microbiota immaturity in malnourished Bangladeshi children. *Nature* **510**, 417–421 (2014). doi:10.1038/nature13421 Medline

3. World Health Organization Department of Nutrition for Health and Development, WHO child growth standards. Length/height-for-age, weight-for-age, weight-for-length, weight-for-height and body mass index-for-age: methods and development (2000); www.who.int/childgrowth/en

4. L. V. Blanton, M. R. Charbonneau, T. Salih, M. J. Barratt, S. Venkatesh, O. Ilkaveya, S. Subramanian, M. J. Manary, I. Trehan, J. M. Jorgensen, Y. M. Fan, B. Henrissat, S. A. Leyn, D. A. Rodionov, A. L. Osterman, K. M. Maleta, C. B. Newgard, P. Ashorn, K. G. Dewey, J. I. Gordon, Gut bacteria that prevent growth impairments transmitted by microbiota from malnourished children. *Science* **351**, aad3311 (2016). doi:10.1126/science.aad3311 Medline

5. World Health Organization (WHO), *Infant and Young Child feeding*; fact sheet no. 342, 1–5 (WHO, 2016).

6. L. Manikam, A. Robinson, J. Y. Kuah, H. J. Vaidya, E. C. Alexander, G. W. Miller, K. K. Singh, V. Dawe, S. Ahmed, R. Lingam, M. Lakhanpaul, A systematic review of complementary feeding practices in South Asian infants and young children: The Bangladesh perspective. *BMC Nutr*. **3**, 56 (2017). doi:10.1186/s40795-017-0176-9

7. H. Sandige, M. J. Ndekha, A. Briend, P. Ashorn, M. J. Manary, Home-based treatment of malnourished Malawian children with locally produced or imported ready-to-use food. *J. Pediatr. Gastroenterol. Nutr.* **39**, 141–146 (2004). doi:10.1097/00005176-200408000-00003 Medline

8. L. Gold, J. J. Walker, S. K. Wilcox, S. Williams, Advances in human proteomics at high scale with the SOMAscan proteomics platform. *Nat. Biotechnol.* **29**, 543–549 (2012). Medline

9. B. Lollo, F. Steele, L. Gold, Beyond antibodies: New affinity reagents to unlock the proteome. *Proteomics* **14**, 638–644 (2014). doi:10.1002/pmic.201300187 Medline

10. J. Candia, F. Cheung, Y. Kotliarov, G. Fantoni, B. Sellers, T. Griesman, J. Huang, S. Stuccio, A. Zingone, B. M. Ryan, J. S. Tsang, A. Biancotto, Assessment of variability in the SOMAscan assay. *Sci. Rep.* **7**, 14248 (2017). doi:10.1038/s41598-017-14755-5 Medline

11. S. Bartz, A. Mody, C. Hornik, J. Bain, M. Muehlbauer, T. Kiyimba, E. Kiboneka, R. Stevens, J. Bartlett, J. V. St Peter, C. B. Newgard, M. Freemark, Severe acute malnutrition in childhood: Hormonal and metabolic status at presentation, response to treatment, and predictors of mortality. *J. Clin. Endocrinol. Metab.* **99**, 2128–2137 (2014). doi:10.1210/jc.2013-4018 Medline

12. R. Overbeek, R. Olson, G. D. Pusch, G. J. Olsen, J. J. Davis, T. Disz, R. A. Edwards, S. Gerdes, B. Parrello, M. Shukla, V. Vonstein, A. R. Wattam, F. Xia, R. Stevens, The SEED and the Rapid Annotation of microbial genomes using Subsystems Technology (RAST). *Nucleic Acids Res.* **42**, D206–D214 (2014). doi:10.1093/nar/gkt1226 Medline

13. D. A. Sela, J. Chapman, A. Adeuya, J. H. Kim, F. Chen, T. R. Whitehead, A. Lapidus, D. S. Rokhsar, C. B. Lebrilla, J. B. German, N. P. Price, P. M. Richardson, D. A. Mills, The genome sequence of *Bifidobacterium longum* subsp. *infantis* reveals adaptations for milk utilization within the infant microbiome. *Proc. Natl. Acad. Sci. U.S.A.* **105**, 18964–18969 (2008). doi:10.1073/pnas.0809584105 Medline

~~14. A. S. Raman *et al.*, A sparse covarying unit that describes healthy and impaired human gut microbiota development. *Science* **365**, eaau4735 (2019).~~

~~15~~**14**. T. Ahmed, M. Mahfuz, S. Ireen, A. M. Ahmed, S. Rahman, M. M. Islam, N. Alam, M. I. Hossain, S. M. Rahman, M. M. Ali, F. P. Choudhury, A. Cravioto, Nutrition of children and women in Bangladesh: Trends and directions for the future. *J. Health Popul. Nutr.* **30**, 1–11 (2012). doi:10.3329/jhpn.v30i1.11268 Medline

~~16~~**15**. L. L. Iannotti, C. K. Lutter, C. P. Stewart, C. A. Gallegos Riofrío, C. Malo, G. Reinhart, A. Palacios, C. Karp, M. Chapnick, K. Cox, W. F. Waters, Eggs in early complementary feeding and child growth: A randomized controlled trial. *Pediatrics* **140**, e20163459 (2017). doi:10.1542/peds.2016-3459 Medline

~~17~~**16**. W. R. Russell, S. H. Duncan, L. Scobbie, G. Duncan, L. Cantlay, A. G. Calder, S. E. Anderson, H. J. Flint, Major phenylpropanoid-derived metabolites in the human gut can arise from microbial fermentation of protein. *Mol. Nutr. Food Res.* **57**, 523–535 (2013). doi:10.1002/mnfr.201200594 Medline

~~18~~**17**. D. Krause, H.-S. Suh, L. Tarassishin, Q. L. Cui, B. A. Durafourt, N. Choi, A. Bauman, M. Cosenza-Nashat, J. P. Antel, M.-L. Zhao, S. C. Lee, The tryptophan metabolite 3-hydroxyanthranilic acid plays anti-inflammatory and neuroprotective roles during inflammation: Role of hemeoxygenase-1. *Am. J. Pathol.* **179**, 1360–1372 (2011). doi:10.1016/j.ajpath.2011.05.048 Medline

~~19~~**18**. L. Cervantes-Barragan, J. N. Chai, M. D. Tianero, B. Di Luccia, P. P. Ahern, J. Merriman, V. S. Cortez, M. G. Caparon, M. S. Donia, S. Gilfillan, M. Cella, J. I. Gordon, C.-S. Hsieh, M. Colonna, *Lactobacillus reuteri* induces gut intraepithelial $CD4^+CD8\alpha\alpha^+$ T cells. *Science* **357**, 806–810 (2017). doi:10.1126/science.aah5825 Medline

~~20~~**19**. G. Das, M. M. Augustine, J. Das, K. Bottomly, P. Ray, A. Ray, An important regulatory role for $CD4^+CD8\ \alpha\ \alpha$ T cells in the intestinal epithelial layer in the prevention of inflammatory bowel disease. *Proc. Natl. Acad. Sci. U.S.A.* **100**, 5324–5329 (2003). doi:10.1073/pnas.0831037100 Medline

~~21~~**20**. H. Cheroutre, M. M. Husain, CD4 CTL: Living up to the challenge. *Semin. Immunol.* **25**, 273–281 (2013). doi:10.1016/j.smim.2013.10.022 Medline

~~22~~**21**. T. Sujino, M. London, D. P. Hoytema van Konijnenburg, T. Rendon, T. Buch, H. M. Silva, J. J. Lafaille, B. S. Reis, D. Mucida, Tissue adaptation of regulatory and intraepithelial $CD4^+$ T cells controls gut inflammation. *Science* **352**, 1581–1586 (2016). doi:10.1126/science.aaf3892 Medline

~~23~~22. R. A. Saxton, D. M. Sabatini, mTOR signaling in growth, metabolism, and disease. *Cell* **169**, 361–371 (2017). doi:10.1016/j.cell.2017.03.035 Medline

~~24~~23. J. Yan, J. W. Herzog, K. Tsang, C. A. Brennan, M. A. Bower, W. S. Garrett, B. R. Sartor, A. O. Aliprantis, J. F. Charles, Gut microbiota induce IGF-1 and promote bone formation and growth. *Proc. Natl. Acad. Sci. U.S.A.* **113**, E7554–E7563 (2016). doi:10.1073/pnas.1607235113 Medline

~~25~~24. M. Schwarzer, K. Makki, G. Storelli, I. Machuca-Gayet, D. Srutkova, P. Hermanova, M. E. Martino, S. Balmand, T. Hudcovic, A. Heddi, J. Rieusset, H. Kozakova, H. Vidal, F. Leulier, *Lactobacillus plantarum* strain maintains growth of infant mice during chronic undernutrition. *Science* **351**, 854–857 (2016). doi:10.1126/science.aad8588 Medline

~~26~~25. D. J. Millward, Amino acid scoring patterns for protein quality assessment. *Br. J. Nutr.* **108** (Suppl 2), S31–S43 (2012). doi:10.1017/S0007114512002462 Medline

~~27~~26. P. L. Altman, D. S. Dittmer, *Growth, Including Reproduction and Morphological Development* (Federation of American Societies for Experimental Biology, 1962).

~~28~~27. M. R. Charbonneau, D. O'Donnell, L. V. Blanton, S. M. Totten, J. C. C. Davis, M. J. Barratt, J. Cheng, J. Guruge, M. Talcott, J. R. Bain, M. J. Muehlbauer, O. Ilkayeva, C. Wu, T. Struckmeyer, D. Barile, C. Mangani, J. Jorgensen, Y. M. Fan, K. Maleta, K. G. Dewey, P. Ashorn, C. B. Newgard, C. Lebrilla, D. A. Mills, J. I. Gordon, Sialylated milk oligosaccharides promote microbiota-dependent growth in models of infant undernutrition. *Cell* **164**, 859–871 (2016). doi:10.1016/j.cell.2016.01.024 Medline

~~29~~28. U.S. Department of Agriculture (USDA), "Commercial Item Description: Ready-to-Use Therapeutic Food (RUTF)" A-A-20363B (USDA, 2012).

~~30~~29. I. Antonow-Schlorke, M. Schwab, L. A. Cox, C. Li, K. Stuchlik, O. W. Witte, P. W. Nathanielsz, T. J. McDonald, Vulnerability of the fetal primate brain to moderate reduction in maternal global nutrient availability. *Proc. Natl. Acad. Sci. U.S.A.* **108**, 3011–3016 (2011). doi:10.1073/pnas.1009838108 Medline

~~31~~30. K. Taniguchi, M. Karin, NF-κB, inflammation, immunity and cancer: Coming of age. *Nat. Rev. Immunol.* **18**, 309–324 (2018). doi:10.1038/nri.2017.142 Medline

31. A. S. Raman *et al.*, A sparse covarying unit that describes healthy and impaired human gut microbiota development. *Science* 365, eaau4735 (2019).

32. M. E. Ritchie, B. Phipson, D. Wu, Y. Hu, C. W. Law, W. Shi, G. K. Smyth, limma powers differential expression analyses for RNA-sequencing and microarray studies. *Nucleic Acids Res.* 43, e47 (2015). doi:10.1093/nar/gkv007 Medline

33. C. Simillion, R. Liechti, H. E. L. Lischer, V. Ioannidis, R. Bruggmann, Avoiding the pitfalls of gene set enrichment analysis with SetRank. *BMC Bioinformatics* 18, 151 (2017). doi:10.1186/s12859-017-1571-6 Medline

~~32~~34. T. Ahmed, M. Ali, M. M. Ullah, I. A. Choudhury, M. E. Haque, M. A. Salam, G. H. Rabbani, R. M. Suskind, G. J. Fuchs, Mortality in severely malnourished children with diarrhoea and use of a standardised management protocol. *Lancet* **353**, 1919–1922 (1999). doi:10.1016/S0140-6736(98)07499-6 Medline

~~33~~35. N. Choudhury, T. Ahmed, M. I. Hossain, M. M. Islam, S. A. Sarker, M. Zeilani, J. D. Clemens, Ready-to-Use Therapeutic food made from locally available food ingredients is well accepted by children having severe acute malnutrition in Bangladesh. *Food Nutr. Bull.* **39**, 116–126 (2018). doi:10.1177/0379572117743929 Medline

~~34~~36. C. B. Newgard, J. An, J. R. Bain, M. J. Muehlbauer, R. D. Stevens, L. F. Lien, A. M. Haqq, S. H. Shah, M. Arlotto, C. A. Slentz, J. Rochon, D. Gallup, O. Ilkayeva, B. R. Wenner, W. S. Yancy Jr., H. Eisenson, G. Musante, R. S. Surwit, D. S. Millington, M. D. Butler, L. P. Svetkey, A branched-chain amino acid-related metabolic signature that differentiates obese and lean humans and contributes to insulin resistance. *Cell Metab.* **9**, 311–326 (2009). doi:10.1016/j.cmet.2009.02.002 Medline

~~35~~37. C. T. Ferrara, P. Wang, E. C. Neto, R. D. Stevens, J. R. Bain, B. R. Wenner, O. R. Ilkayeva, M. P. Keller, D. A. Blasiole, C. Kendziorski, B. S. Yandell, C. B. Newgard, A. D. Attie, Genetic networks of liver metabolism revealed by integration of metabolic and transcriptional profiling. *PLOS Genet.* **4**, e1000034 (2008). doi:10.1371/journal.pgen.1000034 Medline

~~36~~38. M. V. Jensen, J. W. Joseph, O. Ilkayeva, S. Burgess, D. Lu, S. M. Ronnebaum, M. Odegaard, T. C. Becker, A. D. Sherry, C. B. Newgard, Compensatory responses to pyruvate carboxylase suppression in islet β-cells. Preservation of glucose-stimulated insulin secretion. *J. Biol. Chem.* **281**, 22342–22351 (2006). doi:10.1074/jbc.M604350200 Medline

~~37~~39. C. Magnes, F. M. Sinner, W. Regittnig, T. R. Pieber, LC/MS/MS method for quantitative determination of long-chain fatty acyl-CoAs. *Anal. Chem.* **77**, 2889–2894 (2005). doi:10.1021/ac048314i Medline

~~38~~40. P. J. White, A. L. Lapworth, J. An, L. Wang, R. W. McGarrah, R. D. Stevens, O. Ilkayeva, T. George, M. J. Muehlbauer, J. R. Bain, J. K. Trimmer, M. J. Brosnan, T. P. Rolph, C. B. Newgard, Branched-chain amino acid restriction in Zucker-fatty rats improves muscle insulin sensitivity by enhancing efficiency of fatty acid oxidation and acyl-glycine export. *Mol. Metab.* **5**, 538–551 (2016). doi:10.1016/j.molmet.2016.04.006 Medline

~~39. M. E. Ritchie, B. Phipson, D. Wu, Y. Hu, C. W. Law, W. Shi, G. K. Smyth, limma powers differential expression analyses for RNA-sequencing and microarray studies. *Nucleic Acids Res.* **43**, e47 (2015). doi:10.1093/nar/gkv007 Medline~~

~~40. C. Simillion, R. Liechti, H. E. L. Lischer, V. Ioannidis, R. Bruggmann, Avoiding the pitfalls of gene set enrichment analysis with SetRank. *BMC Bioinformatics* **18**, 151 (2017). doi:10.1186/s12859-017-1571-6 Medline~~

41. J. G. Caporaso, J. Kuczynski, J. Stombaugh, K. Bittinger, F. D. Bushman, E. K. Costello, N. Fierer, A. G. Peña, J. K. Goodrich, J. I. Gordon, G. A. Huttley, S. T. Kelley, D. Knights, J. E. Koenig, R. E. Ley, C. A. Lozupone, D. McDonald, B. D. Muegge, M. Pirrung, J. Reeder, J. R. Sevinsky, P. J. Turnbaugh, W. A. Walters, J. Widmann, T. Yatsunenko, J. Zaneveld, R. Knight, QIIME allows analysis of high-throughput community sequencing data. *Nat. Methods* **7**, 335–336 (2010). doi:10.1038/nmeth.f.303 Medline

42. J. D. Planer, Y. Peng, A. L. Kau, L. V. Blanton, I. M. Ndao, P. I. Tarr, B. B. Warner, J. I. Gordon, Development of the gut microbiota and mucosal IgA responses in twins and gnotobiotic mice. *Nature* **534**, 263–266 (2016). doi:10.1038/nature17940 Medline

43. M. Baym, S. Kryazhimskiy, T. D. Lieberman, H. Chung, M. M. Desai, R. Kishony, Inexpensive multiplexed library preparation for megabase-sized genomes. *PLOS ONE* **10**, e0128036 (2015). doi:10.1371/journal.pone.0128036 Medline

44. N. A. Joshi, J. N. Fass, Sickle: A sliding-window, adaptive, quality-based trimming tool for FastQ files, version 1.33 [software]; available at https://github.com/najoshi/sickle

45. M. Martin, Cutadapt removes adapter sequences from high-throughput sequencing reads. *EMBnet* **17**, 10–12 (2011). doi:10.14806/ej.17.1.200

46. Y. Peng, H. C. M. Leung, S. M. Yiu, F. Y. L. Chin, IDBA-UD: A de novo assembler for single-cell and metagenomic sequencing data with highly uneven depth. *Bioinformatics* **28**, 1420–1428 (2012). doi:10.1093/bioinformatics/bts174 Medline

47. T. Seemann, Prokka: Rapid prokaryotic genome annotation. *Bioinformatics* **30**, 2068–2069 (2014). doi:10.1093/bioinformatics/btu153 Medline

48. Y. Liao, G. K. Smyth, W. Shi, featureCounts: An efficient general purpose program for assigning sequence reads to genomic features. *Bioinformatics* **30**, 923–930 (2014). doi:10.1093/bioinformatics/btt656 Medline

49. R Core Team, R: A language and environment for statistical computing R. (Foundation for Statistical Computing, 2017; available at www.R-project.org.

50. M. Steinegger, J. Söding, MMseqs2 enables sensitive protein sequence searching for the analysis of massive data sets. *Nat. Biotechnol.* **35**, 1026–1028 (2017). doi:10.1038/nbt.3988 Medline

51. B. Buchfink, C. Xie, D. H. Huson, Fast and sensitive protein alignment using DIAMOND. *Nat. Methods* **12**, 59–60 (2015). doi:10.1038/nmeth.3176 Medline

52. J. Liu, J. Gratz, C. Amour, G. Kibiki, S. Becker, L. Janaki, J. J. Verweij, M. Taniuchi, S. U. Sobuz, R. Haque, D. M. Haverstick, E. R. Houpt, A laboratory-developed TaqMan Array Card for simultaneous detection of 19 enteropathogens. *J. Clin. Microbiol.* **51**, 472–480 (2013). doi:10.1128/JCM.02658-12 Medline

53. MAL-ED Network Investigators, The Malnutrition and Enteric Disease Study (MAL-ED): Understanding the Consequences for Child Health and Development. *Clin. Infect. Dis.* **59**, S193–S206 (2014).

54. M. Kosek, R. L. Guerrant, G. Kang, Z. Bhutta, P. P. Yori, J. Gratz, M. Gottlieb, D. Lang, G. Lee, R. Haque, C. J. Mason, T. Ahmed, A. Lima, W. A. Petri, E. Houpt, M. P. Olortegui, J. C. Seidman, E. Mduma, A. Samie, S. Babji; MAL-ED Network Investigators, Assessment of environmental enteropathy in the MAL-ED cohort study: Theoretical and analytic framework. *Clin. Infect. Dis.* **59**, S239–S247 (2014). doi:10.1093/cid/ciu457 Medline

55. A. L. Goodman, G. Kallstrom, J. J. Faith, A. Reyes, A. Moore, G. Dantas, J. I. Gordon, Extensive personal human gut microbiota culture collections characterized and manipulated in gnotobiotic mice. *Proc. Natl. Acad. Sci. U.S.A.* **108**, 6252–6257 (2011). doi:10.1073/pnas.1102938108 Medline

56. N. Dey, V. E. Wagner, L. V. Blanton, J. Cheng, L. Fontana, R. Haque, T. Ahmed, J. I. Gordon, Regulators of gut motility revealed by a gnotobiotic model of diet-microbiome

interactions related to travel. *Cell* **163**, 95–107 (2015). doi:10.1016/j.cell.2015.08.059 Medline

57. M. Wu, N. P. McNulty, D. A. Rodionov, M. S. Khoroshkin, N. W. Griffin, J. Cheng, P. Latreille, R. A. Kerstetter, N. Terrapon, B. Henrissat, A. L. Osterman, J. I. Gordon, Genetic determinants of *in vivo* fitness and diet responsiveness in multiple human gut *Bacteroides*. *Science* **350**, aac5992 (2015). doi:10.1126/science.aac5992 Medline

58. S. Turner, K. M. Pryer, V. P. W. Miao, J. D. Palmer, Investigating deep phylogenetic relationships among cyanobacteria and plastids by small subunit rRNA sequence analysis. *J. Eukaryot. Microbiol.* **46**, 327–338 (1999). doi:10.1111/j.1550-7408.1999.tb04612.x Medline

59. S. Kurtz, A. Phillippy, A. L. Delcher, M. Smoot, M. Shumway, C. Antonescu, S. L. Salzberg, Versatile and open software for comparing large genomes. *Genome Biol.* **5**, R12 (2004). doi:10.1186/gb-2004-5-2-r12 Medline

60. A. Bankevich, S. Nurk, D. Antipov, A. A. Gurevich, M. Dvorkin, A. S. Kulikov, V. M. Lesin, S. I. Nikolenko, S. Pham, A. D. Prjibelski, A. V. Pyshkin, A. V. Sirotkin, N. Vyahhi, G. Tesler, M. A. Alekseyev, P. A. Pevzner, SPAdes: A new genome assembly algorithm and its applications to single-cell sequencing. *J. Comput. Biol.* **19**, 455–477 (2012). doi:10.1089/cmb.2012.0021 Medline

61. M. Kanehisa, S. Goto, KEGG: Kyoto Encyclopedia of Genes and Genomes. *Nucleic Acids Res.* **28**, 27–30 (2000). doi:10.1093/nar/28.1.27 Medline

62. R. Overbeek, T. Begley, R. M. Butler, J. V. Choudhuri, H. Y. Chuang, M. Cohoon, V. de Crécy-Lagard, N. Diaz, T. Disz, R. Edwards, M. Fonstein, E. D. Frank, S. Gerdes, E. M. Glass, A. Goesmann, A. Hanson, D. Iwata-Reuyl, R. Jensen, N. Jamshidi, L. Krause, M. Kubal, N. Larsen, B. Linke, A. C. McHardy, F. Meyer, H. Neuweger, G. Olsen, R. Olson, A. Osterman, V. Portnoy, G. D. Pusch, D. A. Rodionov, C. Rückert, J. Steiner, R. Stevens, I. Thiele, O. Vassieva, Y. Ye, O. Zagnitko, V. Vonstein, The subsystems approach to genome annotation and its use in the project to annotate 1000 genomes. *Nucleic Acids Res.* **33**, 5691–5702 (2005). doi:10.1093/nar/gki866 Medline

63. R. K. Aziz, D. Bartels, A. A. Best, M. DeJongh, T. Disz, R. A. Edwards, K. Formsma, S. Gerdes, E. M. Glass, M. Kubal, F. Meyer, G. J. Olsen, R. Olson, A. L. Osterman, R. A. Overbeek, L. K. McNeil, D. Paarmann, T. Paczian, B. Parrello, G. D. Pusch, C. Reich, R. Stevens, O. Vassieva, V. Vonstein, A. Wilke, O. Zagnitko, The RAST Server: Rapid annotations using subsystems technology. *BMC Genomics* **9**, 75 (2008). doi:10.1186/1471-2164-9-75 Medline

64. M. Rajilić-Stojanović, W. M. de Vos, The first 1000 cultured species of the human gastrointestinal microbiota. *FEMS Microbiol. Rev.* **38**, 996–1047 (2014). doi:10.1111/1574-6976.12075 Medline

65. P. S. Novichkov, A. E. Kazakov, D. A. Ravcheev, S. A. Leyn, G. Y. Kovaleva, R. A. Sutormin, M. D. Kazanov, W. Riehl, A. P. Arkin, I. Dubchak, D. A. Rodionov, RegPrecise 3.0—A resource for genome-scale exploration of transcriptional regulation in bacteria. *BMC Genomics* **14**, 745 (2013). doi:10.1186/1471-2164-14-745 Medline

66. C. Abreu-Goodger, E. Merino, RibEx: A web server for locating riboswitches and other conserved bacterial regulatory elements. *Nucleic Acids Res.* **33**, W690-2 (2005). doi:10.1093/nar/gki445 Medline

67. N. P. McNulty, M. Wu, A. R. Erickson, C. Pan, B. K. Erickson, E. C. Martens, N. A. Pudlo, B. D. Muegge, B. Henrissat, R. L. Hettich, J. I. Gordon, Effects of diet on resource utilization by a model human gut microbiota containing Bacteroides cellulosilyticus WH2, a symbiont with an extensive glycobiome. *PLOS Biol.* **11**, e1001637 (2013). doi:10.1371/journal.pbio.1001637 Medline

68. M. C. Hibberd, M. Wu, D. A. Rodionov, X. Li, J. Cheng, N. W. Griffin, M. J. Barratt, R. J. Giannone, R. L. Hettich, A. L. Osterman, J. I. Gordon, The effects of micronutrient deficiencies on bacterial species from the human gut microbiota. *Sci. Transl. Med.* **9**, eaal4069 (2017). doi:10.1126/scitranslmed.aal4069 Medline

69. M. I. Love, W. Huber, S. Anders, Moderated estimation of fold change and dispersion for RNA-seq data with DESeq2. *Genome Biol.* **15**, 550 (2014). doi:10.1186/s13059-014-0550-8 Medline

70. G. Yu, L.-G. Wang, Y. Han, Q.-Y. He, clusterProfiler: An R package for comparing biological themes among gene clusters. *OMICS* **16**, 284–287 (2012). doi:10.1089/omi.2011.0118 Medline

71. W. Luo, M. S. Friedman, K. Shedden, K. D. Hankenson, P. J. Woolf, GAGE: Generally applicable gene set enrichment for pathway analysis. *BMC Bioinformatics* **10**, 161 (2009). doi:10.1186/1471-2105-10-161 Medline

72. X. Wei, H. Song, L. Yin, M. G. Rizzo, R. Sidhu, D. F. Covey, D. S. Ory, C. F. Semenkovich, Fatty acid synthesis configures the plasma membrane for inflammation in diabetes. *Nature* **539**, 294–298 (2016). doi:10.1038/nature20117 Medline

73. T. Clavel, G. Henderson, W. Engst, J. Doré, M. Blaut, Phylogeny of human intestinal bacteria that activate the dietary lignan secoisolariciresinol diglucoside. *FEMS Microbiol. Ecol.* **55**, 471–478 (2006). doi:10.1111/j.1574-6941.2005.00057.x Medline

74. R. Martín, S. Miquel, L. Benevides, C. Bridonneau, V. Robert, S. Hudault, F. Chain, O. Berteau, V. Azevedo, J. M. Chatel, H. Sokol, L. G. Bermúdez-Humarán, M. Thomas, P. Langella, Functional characterization of novel *Faecalibacterium prausnitzii* strains isolated from healthy volunteers: A step forward in the use of *F. prausnitzii* as a next-generation probiotic isolation of novel extremely oxygen. *Front. Microbiol.* **8**, 1226 (2017). doi:10.3389/fmicb.2017.01226 Medline

75. H. Sokol, B. Pigneur, L. Watterlot, O. Lakhdari, L. G. Bermúdez-Humarán, J.-J. Gratadoux, S. Blugeon, C. Bridonneau, J.-P. Furet, G. Corthier, C. Grangette, N. Vasquez, P. Pochart, G. Trugnan, G. Thomas, H. M. Blottière, J. Doré, P. Marteau, P. Seksik, P. Langella, *Faecalibacterium prausnitzii* is an anti-inflammatory commensal bacterium identified by gut microbiota analysis of Crohn disease patients. *Proc. Natl. Acad. Sci. U.S.A.* **105**, 16731–16736 (2008). doi:10.1073/pnas.0804812105 Medline

76. P. Vassilyadi, S. V. Harding, T. J. Hazell, H. A. Weiler, L. J. Wykes, Colitis, independent of macronutrient intake, compromises bone structure and strength in growing piglets. *Pediatr. Res.* **80**, 753–758 (2016). doi:10.1038/pr.2016.135 Medline

77. T. Hildebrand, P. Rüegsegger, Quantification of bone microarchitecture with the structure model index. *Comput. Methods Biomech. Biomed. Engin.* **1**, 15–23 (1997). doi:10.1080/01495739708936692 Medline

78. T. Masuda, M. Tomita, Y. Ishihama, Phase transfer surfactant-aided trypsin digestion for membrane proteome analysis. *J. Proteome Res.* **7**, 731–740 (2008). doi:10.1021/pr700658q Medline

79. S. M. Clarkson, R. J. Giannone, D. M. Kridelbaugh, J. G. Elkins, A. M. Guss, J. K. Michener, Construction and optimization of a heterologous pathway for protocatechuate catabolism in *Escherichia coli* enables bioconversion of model aromatic compounds. *Appl. Environ. Microbiol.* **83**, e01313–e01317 (2017). doi:10.1128/AEM.01313-17 Medline

80. D. L. Tabb, C. G. Fernando, M. C. Chambers, MyriMatch: Highly accurate tandem mass spectral peptide identification by multivariate hypergeometric analysis. *J. Proteome Res.* **6**, 654–661 (2007). doi:10.1021/pr0604054 Medline

81. Z. Q. Ma, S. Dasari, M. C. Chambers, M. D. Litton, S. M. Sobecki, L. J. Zimmerman, P. J. Halvey, B. Schilling, P. M. Drake, B. W. Gibson, D. L. Tabb, IDPicker 2.0: Improved protein assembly with high discrimination peptide identification filtering. *J. Proteome Res.* **8**, 3872–3881 (2009). doi:10.1021/pr900360j Medline

82. R. C. Edgar, Search and clustering orders of magnitude faster than BLAST. *Bioinformatics* **26**, 2460–2461 (2010). doi:10.1093/bioinformatics/btq461 Medline

83. G. Baumann, Growth hormone binding protein. The soluble growth hormone receptor. *Minerva Endocrinol.* **27**, 265–276 (2002). Medline

84. M. H. Rasmussen, K. K. Y. Ho, L. Kjems, J. Hilsted, Serum growth hormone-binding protein in obesity: Effect of a short-term, very low calorie diet and diet-induced weight loss. *J. Clin. Endocrinol. Metab.* **81**, 1519–1524 (1996). Medline

85. S.-H. Tan, A. Lee, D. Pascovici, N. Care, V. Birzniece, K. Ho, M. P. Molloy, A. Khan, Plasma biomarker proteins for detection of human growth hormone administration in athletes. *Sci. Rep.* **7**, 10039 (2017). doi:10.1038/s41598-017-09968-7 Medline

86. T. A. Pinheiro, A. S. Barcala-Jorge, J. M. O. Andrade, T. A. Pinheiro, E. C. N. Ferreira, T. S. Crespo, G. C. Batista-Jorge, C. A. Vieira, D. F. Lelis, A. F. Paraíso, U. B. Pinheiro, M. Bertagnolli, C. J. B. Albuquerque, A. L. S. Guimarães, A. M. B. de Paula, A. P. Caldeira, S. H. S. Santos, Obesity and malnutrition similarly alter the renin-angiotensin system and inflammation in mice and human adipose. *J. Nutr. Biochem.* **48**, 74–82 (2017). doi:10.1016/j.jnutbio.2017.06.008 Medline

87. L. Lerner, T. G. Hayes, N. Tao, B. Krieger, B. Feng, Z. Wu, R. Nicoletti, M. I. Chiu, J. Gyuris, J. M. Garcia, Plasma growth differentiation factor 15 is associated with weight loss and mortality in cancer patients. *J. Cachexia Sarcopenia Muscle* **6**, 317–324 (2015). doi:10.1002/jcsm.12033 Medline

88. T. Wang, J. Liu, C. McDonald, K. Lupino, X. Zhai, B. J. Wilkins, H. Hakonarson, L. Pei, GDF15 is a heart-derived hormone that regulates body growth. *EMBO Mol. Med.* **9**, 1150–1164 (2017). doi:10.15252/emmm.201707604 Medline

89. J. B. Allard, C. Duan, IGF-Binding Proteins: Why do they exist and why are there so many? *Front. Endocrinol.* **9**, 117 (2018). doi:10.3389/fendo.2018.00117 Medline

90. A. Hoeflich, V. C. Russo, Physiology and pathophysiology of IGFBP-1 and IGFBP-2 - consensus and dissent on metabolic control and malignant potential. *Best Pract. Res. Clin. Endocrinol. Metab.* **29**, 685–700 (2015). doi:10.1016/j.beem.2015.07.002 Medline

91. C. Gyrup, C. Oxvig, Quantitative analysis of insulin-like growth factor-modulated proteolysis of insulin-like growth factor binding protein-4 and -5 by pregnancy-associated plasma protein-A. *Biochemistry* **46**, 1972–1980 (2007). doi:10.1021/bi062229i Medline

92. X. Qin, J. E. Wergedal, M. Rehage, K. Tran, J. Newton, P. Lam, D. J. Baylink, S. Mohan, Pregnancy-associated plasma protein-A increases osteoblast proliferation in vitro and bone formation in vivo. *Endocrinology* **147**, 5653–5661 (2006). doi:10.1210/en.2006-1055 Medline

93. M. Rehage, S. Mohan, J. E. Wergedal, B. Bonafede, K. Tran, D. Hou, D. Phang, A. Kumar, X. Qin, Transgenic overexpression of pregnancy-associated plasma protein-A increases the somatic growth and skeletal muscle mass in mice. *Endocrinology* **148**, 6176–6185 (2007). doi:10.1210/en.2007-0274 Medline

94. S. Kløverpris, J. H. Mikkelsen, J. H. Pedersen, M. R. Jepsen, L. S. Laursen, S. V. Petersen, C. Oxvig, Stanniocalcin-1 potently inhibits the proteolytic activity of the metalloproteinase pregnancy-associated plasma protein-A. *J. Biol. Chem.* **290**, 21915–21924 (2015). doi:10.1074/jbc.M115.650143 Medline

95. R. Varghese, A. D. Gagliardi, P. E. Bialek, S.-P. Yee, G. F. Wagner, G. E. Dimattia, Overexpression of human stanniocalcin affects growth and reproduction in transgenic mice. *Endocrinology* **143**, 868–876 (2002). doi:10.1210/endo.143.3.8671 Medline

96. M. F. Gude, R. Hjortebjerg, C. Oxvig, A. A. Thyø, N. E. Magnusson, M. Bjerre, S. B. Pedersen, J. Frystyk, PAPP-A, IGFBP-4 and IGF-II are secreted by human adipose tissue cultures in a depot-specific manner. *Eur. J. Endocrinol.* **175**, 509–519 (2016). doi:10.1530/EJE-16-0569 Medline

97. C. Oxvig, The role of PAPP-A in the IGF system: Location, location, location. *J. Cell Commun. Signal.* **9**, 177–187 (2015). doi:10.1007/s12079-015-0259-9 Medline

98. M. A. Underwood, J. B. German, C. B. Lebrilla, D. A. Mills, *Bifidobacterium longum* subspecies *infantis*: Champion colonizer of the infant gut. *Pediatr. Res.* **77**, 229–235 (2015). doi:10.1038/pr.2014.156 Medline

99. G. A. McQuibban, J. H. Gong, E. M. Tam, C. A. McCulloch, I. Clark-Lewis, C. M. Overall, Inflammation dampened by gelatinase A cleavage of monocyte chemoattractant protein-3. *Science* **289**, 1202–1206 (2000). doi:10.1126/science.289.5482.1202 Medline

100. L. Davis, Y. Chen, M. Sen, WISP-3 functions as a ligand and promotes superoxide dismutase activity. *Biochem. Biophys. Res. Commun.* **342**, 259–265 (2006). doi:10.1016/j.bbrc.2006.01.132 Medline

101. C. G. Kleer, Y. Zhang, Q. Pan, G. Gallagher, M. Wu, Z.-F. Wu, S. D. Merajver, WISP3 and RhoC guanosine triphosphatase cooperate in the development of inflammatory breast cancer. *Breast Cancer Res.* **6**, R110–R115 (2004). doi:10.1186/bcr755 Medline

102. J. Y. Song, A. M. Holtz, J. M. Pinskey, B. L. Allen, Distinct structural requirements for CDON and BOC in the promotion of Hedgehog signaling. *Dev. Biol.* **402**, 239–252 (2015). doi:10.1016/j.ydbio.2015.03.015 Medline

103. F. Kugimiya, S. Ohba, K. Nakamura, H. Kawaguchi, U. I. Chung, Physiological role of bone morphogenetic proteins in osteogenesis. *J. Bone Miner. Metab.* **24**, 95–99 (2006). doi:10.1007/s00774-005-0653-0 Medline

104. A. Pabois, S. Pagie, N. Gérard, C. Laboisse, S. Pattier, P. Hulin, S. Nedellec, C. Toquet, B. Charreau, Notch signaling mediates crosstalk between endothelial cells and macrophages via Dll4 and IL6 in cardiac microvascular inflammation. *Biochem. Pharmacol.* **104**, 95–107 (2016). doi:10.1016/j.bcp.2016.01.016 Medline

105. L. Meng, Z. Bai, S. He, K. Mochizuki, Y. Liu, J. Purushe, H. Sun, J. Wang, H. Yagita, S. Mineishi, H. Fung, G. A. Yanik, R. Caricchio, X. Fan, L. M. Crisalli, E. O. Hexner, R. Reshef, Y. Zhang, Y. Zhang, The Notch ligand DLL4 defines a capability of human dendritic cells in regulating Th1 and Th17 differentiation. *J. Immunol.* **196**, 1070–1080 (2016). doi:10.4049/jimmunol.1501310 Medline

106. K. A. Papadakis, J. L. Prehn, C. Landers, Q. Han, X. Luo, S. C. Cha, P. Wei, S. R. Targan, TL1A synergizes with IL-12 and IL-18 to enhance IFN-γ production in human T cells and NK cells. *J. Immunol.* **172**, 7002–7007 (2004). doi:10.4049/jimmunol.172.11.7002 Medline

107. J. L. Prehn, S. Mehdizadeh, C. J. Landers, X. Luo, S. C. Cha, P. Wei, S. R. Targan, Potential role for TL1A, the new TNF-family member and potent costimulator of IFN-γ, in mucosal inflammation. *Clin. Immunol.* **112**, 66–77 (2004). doi:10.1016/j.clim.2004.02.007 Medline

108. A. J. Prendergast, S. Rukobo, B. Chasekwa, K. Mutasa, R. Ntozini, M. N. N. Mbuya, A. Jones, L. H. Moulton, R. J. Stoltzfus, J. H. Humphrey, Stunting is characterized by chronic inflammation in Zimbabwean infants. *PLOS ONE* **9**, e86928 (2014). doi:10.1371/journal.pone.0086928 Medline

109. S. Miquel, M. Leclerc, R. Martin, F. Chain, M. Lenoir, S. Raguideau, S. Hudault, C. Bridonneau, T. Northen, B. Bowen, L. G. Bermúdez-Humarán, H. Sokol, M. Thomas, P. Langella, Identification of metabolic signatures linked to anti-inflammatory effects of *Faecalibacterium prausnitzii*. *mBio* **6**, e00300–e00315 (2015). doi:10.1128/mBio.00300-15 Medline

110. N. M. Breyner, C. Michon, C. S. de Sousa, P. B. Vilas Boas, F. Chain, V. A. Azevedo, P. Langella, J. M. Chatel, Microbial anti-inflammatory molecule (MAM) from *Faecalibacterium prausnitzii* shows a protective effect on DNBS and DSS-induced colitis model in mice through inhibition of NF-κB pathway. *Front. Microbiol.* **8**, 114 (2017). doi:10.3389/fmicb.2017.00114 Medline

111. D. J. Marchant, C. L. Bellac, T. J. Moraes, S. J. Wadsworth, A. Dufour, G. S. Butler, L. M. Bilawchuk, R. G. Hendry, A. G. Robertson, C. T. Cheung, J. Ng, L. Ang, Z. Luo, K.

Heilbron, M. J. Norris, W. Duan, T. Bucyk, A. Karpov, L. Devel, D. Georgiadis, R. G. Hegele, H. Luo, D. J. Granville, V. Dive, B. M. McManus, C. M. Overall, A new transcriptional role for matrix metalloproteinase-12 in antiviral immunity. *Nat. Med.* **20**, 493–502 (2014). doi:10.1038/nm.3508 Medline

112. R. A. Dean, J. H. Cox, C. L. Bellac, A. Doucet, A. E. Starr, C. M. Overall, Macrophage-specific metalloelastase (MMP-12) truncates and inactivates ELR+ CXC chemokines and generates CCL2, -7, -8, and -13 antagonists: Potential role of the macrophage in terminating polymorphonuclear leukocyte influx. *Blood* **112**, 3455–3464 (2008). doi:10.1182/blood-2007-12-129080 Medline

113. S. Basu, R. J. Binder, R. Suto, K. M. Anderson, P. K. Srivastava, Necrotic but not apoptotic cell death releases heat shock proteins, which deliver a partial maturation signal to dendritic cells and activate the NF-κB pathway. *Int. Immunol.* **12**, 1539–1546 (2000). doi:10.1093/intimm/12.11.1539 Medline

114. A. Murshid, J. Gong, S. K. Calderwood, Heat shock protein 90 mediates efficient antigen cross presentation through the scavenger receptor expressed by endothelial cells-I. *J. Immunol.* **185**, 2903–2917 (2010). doi:10.4049/jimmunol.0903635 Medline

115. Y. Tamura, A. Yoneda, N. Takei, K. Sawada, Spatiotemporal regulation of Hsp90–ligand complex leads to immune activation. *Front. Immunol.* **7**, 201 (2016). doi:10.3389/fimmu.2016.00201 Medline

116. B. M. Yapo, Pineapple and banana pectins comprise fewer homogalacturonan building blocks with a smaller degree of polymerization as compared with yellow passion fruit and lemon pectins: Implication for gelling properties. *Biomacromolecules* **10**, 717–721 (2009). doi:10.1021/bm801490e Medline

117. W. M. U. Fernando, J. E. Hill, G. A. Zello, R. T. Tyler, W. J. Dahl, A. G. Van Kessel, Diets supplemented with chickpea or its main oligosaccharide component raffinose modify faecal microbial composition in healthy adults. *Benef. Microbes* **1**, 197–207 (2010). doi:10.3920/BM2009.0027 Medline

118. S. Fukuda, H. Toh, K. Hase, K. Oshima, Y. Nakanishi, K. Yoshimura, T. Tobe, J. M. Clarke, D. L. Topping, T. Suzuki, T. D. Taylor, K. Itoh, J. Kikuchi, H. Morita, M. Hattori, H. Ohno, Bifidobacteria can protect from enteropathogenic infection through production of acetate. *Nature* **469**, 543–547 (2011). doi:10.1038/nature09646 Medline

119. C. J. Kelly, L. Zheng, E. L. Campbell, B. Saeedi, C. C. Scholz, A. J. Bayless, K. E. Wilson, L. E. Glover, D. J. Kominsky, A. Magnuson, T. L. Weir, S. F. Ehrentraut, C. Pickel, K. A. Kuhn, J. M. Lanis, V. Nguyen, C. T. Taylor, S. P. Colgan, Crosstalk between microbiota-derived short-chain fatty acids and intestinal epithelial HIF augments tissue barrier function. *Cell Host Microbe* **17**, 662–671 (2015). doi:10.1016/j.chom.2015.03.005 Medline

120. P. J. Fielder, D. L. Mortensen, P. Mallet, B. Carlsson, R. C. Baxter, R. G. Clark, Differential long-term effects of insulin-like growth factor-I (IGF-I) growth hormone (GH), and IGF-I plus GH on body growth and IGF binding proteins in hypophysectomized rats. *Endocrinology* **137**, 1913–1920 (1996). doi:10.1210/endo.137.5.8612531 Medline

121. J. Zhao, M. Li, J. P. Bradfield, H. Zhang, F. D. Mentch, K. Wang, P. M. Sleiman, C. E. Kim, J. T. Glessner, C. Hou, B. J. Keating, K. A. Thomas, M. L. Garris, S. Deliard, E. C. Frackelton, F. G. Otieno, R. M. Chiavacci, R. I. Berkowitz, H. Hakonarson, S. F. A. Grant, The role of height-associated loci identified in genome wide association studies in the determination of pediatric stature. *BMC Med. Genet.* **11**, 96 (2010). doi:10.1186/1471-2350-11-96 Medline

122. P. J. McLaughlin, B. Bakall, J. Choi, Z. Liu, T. Sasaki, E. C. Davis, A. D. Marmorstein, L. Y. Marmorstein, Lack of fibulin-3 causes early aging and herniation, but not macular degeneration in mice. *Hum. Mol. Genet.* **16**, 3059–3070 (2007). doi:10.1093/hmg/ddm264 Medline

123. R. C. Meex, A. J. Hoy, A. Morris, R. D. Brown, J. C. Y. Lo, M. Burke, R. J. A. Goode, B. A. Kingwell, M. J. Kraakman, M. A. Febbraio, J. W. Greve, S. S. Rensen, M. P. Molloy, G. I. Lancaster, C. R. Bruce, M. J. Watt, Fetuin-B is a secreted hepatocyte factor linking steatosis to impaired glucose metabolism. *Cell Metab.* **22**, 1078–1089 (2015). doi:10.1016/j.cmet.2015.09.023 Medline

124. J. W. Choi, H. Liu, R. Mukherjee, J. W. Yun, Downregulation of fetuin-B and zinc-α2-glycoprotein is linked to impaired fatty acid metabolism in liver cells. *Cell. Physiol. Biochem.* **30**, 295–306 (2012). doi:10.1159/000339065 Medline
